# Gene-set distance analysis (GSDA): a powerful tool for gene-set association analysis

**DOI:** 10.1186/s12859-021-04110-x

**Published:** 2021-04-21

**Authors:** Xueyuan Cao, Stan Pounds

**Affiliations:** 1 Department of Acute and Tertiary Care, University of Tennessee Health Science Center, Memphis, 38163 USA; 2 Department of Biostatistics, St Jude Children’s Research Hospital, Memphis, 38105 USA

**Keywords:** Gene profiling, Gene set, Distance correlation

## Abstract

**Background:**

Identifying sets of related genes (gene sets) that are empirically associated with a treatment or phenotype often yields valuable biological insights. Several methods effectively identify gene sets in which individual genes have simple monotonic relationships with categorical, quantitative, or censored event-time variables. Some distance-based methods, such as distance correlations, may detect complex non-monotone associations of a gene-set with a quantitative variable that elude other methods. However, the distance correlations have yet to be generalized to associate gene-sets with categorical and censored event-time endpoints. Also, there is a need to determine which genes empirically drive the significance of an association of a gene set with an endpoint.

**Results:**

We develop gene-set distance analysis (GSDA) by generalizing distance correlations to evaluate the association of a gene set with categorical and censored event-time variables. We also develop a backward elimination procedure to identify a subset of genes that empirically drive significant associations. In simulation studies, GSDA more effectively identified complex non-monotone gene-set associations than did six other published methods. In the analysis of a pediatric acute myeloid leukemia (AML) data set, GSDA was the only method to discover that event-free survival (EFS) was associated with the 56-gene AML pathway gene-set, narrow that result down to 5 genes, and confirm the association of those 5 genes with EFS in a separate validation cohort. These results indicate that GSDA effectively identifies and characterizes complex non-monotonic gene-set associations that are missed by other methods.

**Conclusion:**

GSDA is a powerful and flexible method to detect gene-set association with categorical, quantitative, or censored event-time variables, especially to detect complex non-monotonic gene-set associations. Available at https://CRAN.R-project.org/package=GSDA.

**Supplementary information:**

The online version contains supplementary material available at 10.1186/s12859-021-04110-x.

## Background

Biomedical researchers frequently seek to determine which gene pathways or ontologies are affected by a treatment or involved in biological processes that influence a particular phenotype or clinical outcome. Genes act cooperatively in pathways to influence biological processes and subsequently clinical outcomes. There are several public resources that annotate genes to specific pathways and other biological processes or functions. Several analysis methods that combine gene annotations with statistical analysis results to evaluate the association of sets of genes with specific biological annotations with a treatment or outcome have been used to make many scientific discoveries. Maciejewski  [1] has reviewed several of those methods.

Virtaneva et al. [2] first used the significance and function of expression (SAFE) method in a study of acute myeloid leukemia (AML) and then Barry et al.  [3, 4] more fully described SAFE as an analysis method. First, for each gene, SAFE computes a statistic measuring differential expression across two groups. SAFE then ranks individual genes according to that statistic and computes a Wilcoxon rank-sum statistic to compare the ranks of gene-set genes to those of other genes. Finally, the statistical significance (*p* value) of the individual differential expression statistics and the gene-set statistic is determined by repeating the analysis for a series of data sets in which the assignment of group labels to expression profiles has been permuted. SAFE has also been generalized to evaluate associations of gene sets with other phenotypes or endpoints, including quantitative variables and censored event-time variables, such as survival times in oncology studies.

Mootha et al. [5] first used the gene-set enrichment analysis (GSEA) method in a study of diabetes, and then Subramanian et al. [6] more fully described GSEA as an analysis method. The GSEA framework is very similar to that of SAFE, except that GSEA uses an enrichment statistic in place of the Wilcoxon rank-sum statistic in SAFE. GSEA has been generalized to evaluate associations with several types of endpoints and been widely used with much success in the biomedical research literature. Efron and Tibshirani [7] developed the gene-set association (GSA) method by modifying the form of the GSEA enrichment statistic. SAFE, GSEA, and GSA are all methods that compare gene-level association statistics of genes annotated to a gene set with those of other genes and permute the assignment of genomic data to treatment or endpoint data to determine the statistical significance of that comparison.

Goeman et al. [8] proposed the global test (GT) as a different type of gene-set association analysis method. For a given gene set, GT models the contributions of individual member genes as random effects and tests whether the variability of those random effects equals zero. In this way, GT builds upon standard theory for random effects in generalized linear models and can be used to evaluate association of gene-sets with many different treatments, phenotypes, or endpoints. Goeman and Bühlmann [9] also note that SAFE, GSEA, and GSA are *competitive testing procedures* that compare the individual gene-association statistics of genes annotated to the gene set with those of other genes, whereas GT is a *self-contained test* that is a function solely of the gene-set genes and the treatment, phenotype, or endpoint. By building upon generalized linear models, GT is a very powerful method for detection of general linear associations, but its power to detect other forms of association is not well understood.

Irizarry et al. [10] and Ackermann and Strimmer [11] suggest that the total of test statistics (TOTS) or total of squared test statistics for individual genes be used as a gene-set association statistic and its significance be determined by permutation. This approach is a self-contained method like GT but uses permutation like SAFE, GSEA, and GSA. The TOTS framework can be used in conjunction with general linear modeling or proportional hazards modeling and thus can be used to evaluate the association of a gene set with many different variable types, including categorical, quantitative, and censored event times.

Nettleton et al. [12] proposed the multiresponse permutation procedure (MRPP) to evaluate the association of a gene-set with a set of treatments or a categorical endpoint or phenotype. MRPP measures the association of a gene-set with a categorical treatment or endpoint variable by computing the sum of distances between each pair of subjects belonging to the same group. The distance is computed using the data for genes belonging to the gene set. A lesser value of this distance-based association statistic indicates the subjects belonging to the same group have very similar profiles for genes in the gene set and thus indicates a stronger association. The statistical significance (*p* value) of the distance-based association statistic is determined by permutation of the assignment of gene profile data to the categorical labels.

More recently, Cao et al. [13] developed projection onto orthogonal statistical tests (POST) as a general method to evaluate the association of a gene set. POST first computes an orthogonal decomposition (principal components) of the gene-set data, selects a set of components that characterize most of the variation of the original data, and computes a test statistic that evaluates the association of each of those components with the treatment or endpoint variable of interest. Next, a gene-set association statistic is computed as a weighted sum of the components’ squared association statistics. A bootstrap procedure is then used to compute parameter estimates for a weighted chi-square distribution approximation that is used to compute the *p* value.

Väremo et al. [14] developed the platform for integrative analysis of omic (PIANO) package that implements eleven gene-set analysis methods that operate on gene-level statistics or *p* values in one computational framework. PIANO can be used to identify gene-sets that have non-directional, mixed directional, or distinct directional associations with the endpoint or phenotype of interest. Additionally, PIANO allows users to compute consensus results for multiple methods.

Each of the methods described above has its own unique set of strengths and limitations. Among the methods mentioned above, GT is the only method that does not rely on computationally intense permutation or resampling procedures to determine statistical significance. MRPP is the only method that has good power to detect some complex associations, such as gene-sets that show equal mean expression for all genes but differential correlation among genes across categorical groups. Methods that can detect complex associations without relying on permutation or bootstrapping would have great practical value for biological research applications.

Zhu et al. [15] review and develop statistical theory and methods to use various distance correlations to measure linear, monotonic, and non-monotonic associations between two quantitative data matrices. Here, we extend the framework of Zhu et al. [15] to develop gene-set distance analysis (GSDA) as a method to evaluate the association of a gene-set with a categorical, quantitative, or censored event-time variable by adapting distance correlations to those settings. Below, we describe the development of GSDA, conceptually compare it with other methods, and evaluate its performance in simulation studies and an analysis of a publicly available pediatric acute myeloid leukemia (AML) data set.

## Methods

### The distance correlation framework

Zhu et al. [15] review and develop a distance correlation *t*-test framework to statistically evaluate the association between two quantitative data matrices. They show that distance correlations can detect non-monotonic associations with small to moderate sample sizes. These properties indicate that a generalized distance correlation *t*-test may be a statistically robust framework for many practical applications involving gene-set association testing. Below, we briefly describe this distance correlation *t*-test framework and then describe how we adapt it to be applicable in other settings.

First, we introduce some general notation to describe the distance correlation framework of Zhu et al. [15]. Let ${\varvec{X}}$ be a $n \times m$ matrix of the numeric data values of $g=1,\dots ,m$ variables for each of $i=1,\dots ,n$ individuals such that entry $x_{ig}$ has the value of numeric variable *g* for individual *i*. Let $d_x(x_i,x_j)$ be a metric of the distance between any two individuals *i* and *j*. Let ${\varvec{X}}^\star$ be the $n \times n$ matrix of distances between each pair of individuals with entries $x_{ij}^\star = d_x(x_i,x_j)$. Similarly, let ${\varvec{Y}}$ be a $n \times k$ matrix of the numeric data values of a different set of $v=1,\dots ,k$ variables for each of the same set of $i=1,\dots ,n$ individuals with entry $y_{iv}$ representing the data value of variable *v* for subject *i*. Also, let $d_y(y_i,y_j)$ measure the distance between any two individuals *i* and *j* and let ${\varvec{Y}}^\star$ be the $n \times n$ matrix of the distances $y^\star _{ij}=d_y(y_i,y_j)$ for all *i*, *j* pairs of individuals.

Next, for any $n \times$n distance matrix ${\varvec{A}}^\star$ with $n > 2$, the entries ${\tilde{a}}_{ij}$ of the *U-centered distance matrix* $\tilde{{\varvec{A}}}$ are computed as

1 $$\begin{aligned} {\tilde{a}}_{ij} = a^\star _{ij} - \frac{(a^\star _{i\cdot }+a^\star _{\cdot j})}{n-2} +\frac{a^\star _{\cdot \cdot }}{(n-1)(n-2)}. \end{aligned}$$

where $a^\star _{i\cdot } = \sum _{j=1}^n a_{ij}^\star$ is the sum over row *i* and $a^\star _{\cdot j} = \sum _{i=1}^n a^\star _{ij}$ is the sum over column *j* of the distance matrix ${\varvec{A}}^\star$. Now, for any pair of $n \times n$ U-centered distance matrices $\tilde{{\varvec{A}}}$ and $\tilde{{\varvec{B}}}$ with $n>3$, define the *inner product* as

2 $$\begin{aligned} (\tilde{{\varvec{A}}} \cdot \tilde{{\varvec{B}}}) = \frac{1}{n(n-3)}\sum _{i \ne j} {\tilde{a}}_{ij} {\tilde{b}}_{ij}. \end{aligned}$$

With these notations and definitions, the distance correlation between the data matrices ${\varvec{X}}$ and ${\varvec{Y}}$ is defined and computed as

3 $$\begin{aligned} r_d({\varvec{X}},{\varvec{Y}}) = \frac{(\tilde{{\varvec{X}}} \cdot \tilde{{\varvec{Y}})}}{\sqrt{(\tilde{{\varvec{X}}}\cdot \tilde{{\varvec{X}}}) ( \tilde{{\varvec{Y}}} \cdot \tilde{{\varvec{Y}}})}} \end{aligned}$$

where $\tilde{{\varvec{X}}}$ and $\tilde{{\varvec{Y}}}$ are the U-centered distance matrices for the data matrices ${\varvec{X}}$ and ${\varvec{Y}}$. Zhu et al. [15] elegantly show that the distance correlation t-statistic

4 $$\begin{aligned} t_d({\varvec{X}},{\varvec{Y}}) = \frac{r_d({\varvec{X}},{\varvec{Y}})}{\sqrt{(1-r_d^2({\varvec{X}},{\varvec{Y}})}}\sqrt{\frac{n(n-3)}{2}-1} \end{aligned}$$

with $n(n-3)/2-1$ degrees of freedom is a very powerful and well-controlled t-test of the null hypothesis that the two numeric data matrices have no association (i.e., the mutual information is zero) when Euclidean distance is used for $d_x(x_i,x_j)$ and $d_y(y_i,y_j)$.

The distance correlation *t*-test of Zhu et al. [15] in Eq. (4) is technically elegant. Also, its statistical power and Type I error control are rigorously shown by thorough mathematical proofs. It is a statistically rigorous test that can be widely used to evaluate the association of two numeric data matrices (such as gene expression and methylation) in practice. Below, we provide a general overview of GSDA and then propose specific adaptations of the distance correlation *t*-test to make it useful for evaluating the association of a gene-set numeric data matrix ${\varvec{X}}$ with a quantitative variable, a categorical variable, and a censored event-time variable. In each of these three distinct settings, we compute a U-centered data matrix $\tilde{{\varvec{Y}}}$ for the variable of interest in a setting-specific manner and then substitute it into Eqs. (3) and (4) to obtain a correlation and *t*-statistic to describe and test the association. We call this family of methods *gene-set distance analysis* (GSDA) because it uses a distance-testing framework to evaluate the association of a gene-set with a treatment, phenotype, or outcome variable of interest.

**Fig. 1:**
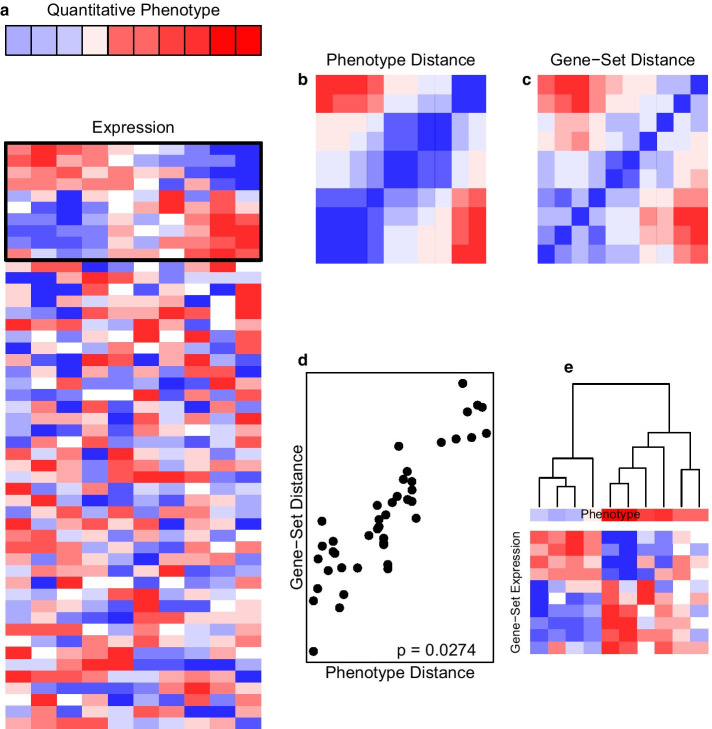
Overview of the gene-set distance analysis (GSDA) method. **a** A color bar of a quantiative phenotype aligned with a heatmap of a gene expression matrix. In both the color bar and heatmap, rows represent variables, colums represent subjects, blue represents low values, and red represents high values. A black box is drawn around the portion of the gene expression heatmap corresponding to a predefined gene-set of scientific interest. The columns are ordered by the phenotype data values. **b** A heatmap of the phenotype distance matrix with blue indicating short distance and red indicating long distance. **c** A similarly colored heatmap of the gene-set gene expression distance matrix. **d** A scatterplot of the gene-set distance and phenotype distance in which each point represents these two distances for one pair of subjects. The GSDA method computes a *p* value of 0.0274 for the association in this illustrative example. **e** Illustrates the association with a dendrogram based on gene expression distance, a color bar for the phenotype data, and a heatmap for the expression data of the gene set

### Overview of gene-set distance analysis

Figure 1 provides a general overview of gene-set distance analysis (GSDA). The initial inputs are a list of genes belonging to the gene set, gene expression matrix, and the endpoint, phenotype, or treatment data which is to be associated with the expression matrix (Fig. 1a). The expression data and phenotype/endpoint/treatment data should be collected for the same set of subjects. GSDA then computes a phenotype distance matrix (Fig. 1b) and an expression distance matrix for the genes in the gene-set (Fig. 1c). Each entry of the distance matrix gives the distance between a pair of subjects in terms of the variable for which the distance was computed. Corresponding entries of the distance matrix are then paired and the correlation of these paired distance matrix entires is evaluated with a distance correlation statistic (Fig. 1d). The statistical significance is computed using the equations of “The distance correlation framework” section above or by permutation as described in “Verifying significant results with permutation” section below. Finally, the result may be visualized with a heatmap (Fig. 1e).

### Associating a gene-set with one quantitative variable

Evaluating the association of the gene-set matrix ${\varvec{X}}$ with one quantitative variable ${\varvec{Y}}$ is a special case of the distance correlation framework described in “The distance correlation framework” section above. The quantitative variable can be represented as a data matrix ${\varvec{Y}}$ with exactly $v=1$ one column of data. For this setting, GSDA uses Euclidean distance to performs all calculations exactly as described above to obtain $r_d$ of Eq. (3) and $t_d$ of Eq. (4).

### Associating a gene-set with one categorical variable

To evaluate the association of a gene-set data matrix ${\varvec{X}}$ with one categorical variable ${\varvec{Y}}$ (one column with the category label for each subject), GSDA first computes each (*i*, *j*) entry of initial distance matrix ${\varvec{Y}}^\star$ for *Y* as

5 $$\begin{aligned} y^\star _{ij} = \mathrm{I}(y_i \ne y_j) \end{aligned}$$

where $y_i$ and $y_j$ are the values of the categorical variable for individuals *i* and *j*, respectively, and $\mathrm{I}(\cdot )$ is the indicator function that equals 1 if the enclosed statement is true and equals 0 if the enclosed statement is false. In other words, for the categorical variable *Y*, the distance between each pair of individuals in the same category is zero and the distance between any pair of individuals in different categories is 1. The initial distance matrix ${\varvec{Y}}^\star$ is then U-centered according to Eq. 1 and the resulting U-centered distance matrix $\tilde{{\varvec{Y}}}$ is substituted into Eqs. (3) and (4) to obtain the distance correlation $r_d$ and *t*-statistic $t_d$.

### Associating a gene-set with one event-time endpoint

Similarly, GSDA evaluates the association of a numeric gene-set matrix ${\varvec{X}}$ with one censored event-time endpoint by computing an initial distance matrix ${\varvec{Y}}^\star$, then U-centering it according to Eq. (1) to obtain the U-centered distance matrix $\tilde{{\varvec{Y}}}$ and finally substituting the U-centered distance matrix into Eqs. (3) and (4) to compute the distance correlation $r_d$ and $t_d$. For each individual $i=1,\dots ,n$, the censored event-time data is represented as a pair ($o_i$,$s_i$) with the observation time and event status of the individual. For each individual that has experienced the event of interest, the event status $s_i=1$ and the observation time $o_i$ is the time elapsed from baseline until the event occurred. For each individual that has not yet experienced the event of interest, the event status $s_i=0$ and the observation time $o_i$ is the time elapsed from baseline until the most recent determination of that individual’s event status. Also, let $l=1,\dots ,\acute{s}$ index the unique observation times $u_1< u_2< \cdots < u_{\acute{s}}$ for the events. Given this information, GSDA computes the initial distance matrix ${\varvec{Y}}^\star$ for the censored event-time variable as

6 $$\begin{aligned} \acute{y}_{ij} = \sum _{l=1}^{\acute{s}} \mathrm{I}(o_j> u_l \ge o_i)\mathrm{I}(s_i=1) +\mathrm{I}(o_i > u_l \ge o_j)\mathrm{I}(s_j = 1). \end{aligned}$$

For each pair of individuals *i* and *j* that have both not yet experienced an event ($s_i=s_j=0$), this distance metric is zero. For each pair of individuals such that individual *i* experiences an event ($s_i=1$) prior to the observation time of individual *j* ($o_i < o_j$), the distance metric is the number of unique event times that occur between $o_i$ and $o_j$. This initial distance matrix ${\varvec{Y}}^\star$ is then substituted into Eq. 1 to obtain the U-centered distance matrix $\tilde{{\varvec{Y}}}$. Finally, the U-centered distance matrix $\tilde{{\varvec{Y}}}$ is substituted into Eqs. (3) and (4) to obtain the distance correlation $r_d$ and its *t*-statistic $t_d$.

The distance metric of Eq. (6) is computed by categorizing subjects in each risk set as being event-free at or having an event prior to each unique event time. Similar techniques in defining and averaging over risk sets are used in classical survival analysis methods such as the log-rank test [16]. Also, this distance metric is similar to the differences of censor-adjusted ranks in the rank-based survival correlation method of Jung et al. [17] and is closely related to the C-index [18].

### Verifying significant results with permutation

The distance correlation *t*-test framework is very useful to quickly identify non-significant results and eliminate them from further consideration. The derivation of the *t*-test relies on asymptotic approximations of the null distribution for evaluating the association of two data matrices. The *t*-test approximation is accurate for most of the *p* value range in each of our simulation studies described in “Simulation studies” section below. Still, like other asymptotic approximations, the *t*-test approximation may not give accurate probabilities for the extreme tails. Thus, the GSDA package includes a permutation module to evaluate the reliability of significant distance correlation *t*-test results. The permutation procedure is very fast because it operates on the the U-centered distance matrices ${\tilde{X}}$ and ${\tilde{Y}}$. Thus, only Eqs. (3) and (4) are computed in the permutation iterations. These equations only involve simple arithmetic operations and thus are completed very quickly. We use this permutation procedure in the illustrative examples and the example application below. For applications that involve evaluating the association of many gene-sets, we recommend using the *t*-test of Eq. (4) to rapidly compute initial *p* values for all gene-sets and then use permutation to ensure that the smallest *p* values do not overstate statistical significance. The *t*-test *p* value may also be used to break ties among gene sets that have the same permutation *p* value.

### Data transformations and distance metrics

In any distance-based analysis framework, the choice of data transformations and distance metric(s) are important considerations. GSDA uses Euclidean distance for the numeric gene-set data matrix ${\varvec{X}}$. In some settings, it may be desirable to transform the data so that each gene has similar variance. This may be accomplished by *z*-score transformation (subtracting the mean and then dividing by the standard deviation) or commesuration (Nettleton et al. [12]) in which data values are centered and then divided by the sum of distances between all pairs of points. These variance-equalizing transformations will ensure that all genes contribute equally to distance calculations; this may be advisable in some applications and not advisable in others. For example, the *z*-score transformation would equalize the contributions of lowly and highly expressed genes to the distance calculations. If highly expressed genes are more biologically influential in the system under study, then variance-equalizing transformations will obscure this effect in the statistical analysis. If highly and lowly expressed genes are both biologically important, then variance-equalizing transformations may be beneficial.

Also, power and logarithmic transformations of ${\varvec{X}}$ will also profoundly impact GSDA; these transformations may be advisable in some applications but not in others. For a quantitative ${\varvec{Y}}$, GSDA also uses Euclidean distance and thus the same principles apply. For a categorical ${\varvec{Y}}$, GSDA uses the categorical distance of Eq. 5, which is impacted by combining groups. For a censored survival time variable ${\varvec{Y}}$, GSDA uses the rank-based metric that is not affected by monotone transformations of the observation times $o_i$. In this work, Euclidean distance is used for numerical variables and the distance metrics for categorical and censored event-time variables are defined above. Future research should explore the how the statistical performance of GSDA is affected by incorporating various other distance metrics (such as cosine distance) and transformations into its calculations.

### Multiple-testing adjustments

The sections above describe how GSDA evaluates the association of one gene-set with one quantitative, one categorical, or one censored event-time variable. The *t*-test of Eq. 4 computes a *p* value for the association of the gene-set with the variable of interest. Frequently in practice one wishes to evaluate the association of each of many gene-sets with the variable of interest. In these settings, GSDA may be applied as described above to evaluate the association of each gene-set with the variable of interest. This will yield many *p* values and require a multiple testing adjustment. In most settings, it will be most reasonable to use these *p* values to estimate or control the false discovery rate (FDR) developed by Benjamini and Hochberg [19]. Several FDR methods are available for this purpose, including those developed by Benjamini and Hochberg [19], Storey [20], and Pounds and Cheng [21]. Pounds [22], Cheng and Pounds [23], and Benjamini [24] have reviewed many of these methods and provided guidance on how to select the best FDR method for particular applications. We recommend users consider those works to choose the best FDR method for their particular applications.

### Comparison with other methods

Several methods have been proposed and used to evaluate the association of a gene-set with a variable of interest, including significance and function of expression (SAFE [2–4]), gene-set enrichment analysis (GSEA [5, 6]), gene-set analysis (GSA [7]), the global test (GT [8, 9]), the total of test statistics (TOTS [10, 11]), the multi-response permutation procedure (MRPP [12]), and projection onto orthogonal statistical tests (POST [13]) as briefly described in the introduction. As shown in Table 1 and described in greater detail below, these methods can be characterized in terms of various properties that have been described in the literature, including the ability to detect linear, monotone, and non-monotone associations; the ability to evaluate associations with quantitative, categorical, or event-time endpoints; being a *self-contained* or *competitive* testing procedure; reliance on statistical model fitting; and use of resampling methods (permutation or bootstrap) to determine statistical significance. GSDA is unique in that it is the only self-contained method that does not use resampling, does not rely on model fitting, can detect many different forms of association, and can evaluate associations with categorical, quantitative, and censored event-time variables. The advantages and limitations of this unique combination of properties are discussed in greater detail below.

**Table 1:** Properties of some gene-set testing methods

Property	GSDA	GS(E)A+SAFE	GT+TOTS+POST	MRPP
Linear	$\checkmark$	$\checkmark$	$\checkmark$	$\checkmark$
Non-linear	$\checkmark$			$\checkmark$
Non-monotone	$\checkmark$			$\checkmark$
Categorical	$\checkmark$	$\checkmark$	$\checkmark$	$\checkmark$
Quantitative	$\checkmark$	$\checkmark$	$\checkmark$	
Event-time	$\checkmark$	$\checkmark$	$\checkmark$	
Self-contained	$\checkmark$		$\checkmark$	$\checkmark$
Competitive		$\checkmark$		
Model-fitting		$\checkmark$	$\checkmark$	
Resampling	$\checkmark$	$\checkmark$	$\checkmark$	$\checkmark$

GS(E)A refers to GSEA and GSA

GSDA is a *self-contained* procedure. For each gene-set, the *p* value is a function of the endpoint and *only* the genes in the gene-set. For *competitive* gene-set testing methods, the *p* value of a gene-set is a function of the endpoint and *all* genes. Competitive procedures compare the associations of gene-set genes with the endpoint to the associations of other genes with the endpoint. Competitive testing procedures seek to find gene-sets for which member genes are more strongly associated with the endpoint of interest than are other genes. Goeman and Bühlmann [9] discuss the advantages and limitations of competitive and self-testing procedures in depth. Briefly, competitive testing procedures can sometimes be difficult to interpret because their results are function of all genes, not just gene-set genes. Also, competitive testing procedures can have less statistical power than self-contained procedures. In many settings, the improved statistical power of self-contained procedures can be advantageous. However, in some settings, self-contained procedures can be “overpowered” in the sense that so many gene-sets are identified as significant that it doesn’t really help the investigator to identify a few gene-sets to more thoroughly evaluate in future research.

GSDA does not require resampling methods such as permutation or bootstraping to compute *p* values. It is well-known that resampling methods dramatically increase the computational time and burden of an analysis because these methods require repeating an analysis procedure for each of many data sets generated by resampling. Bootstrapping and permutation are useful techniques to accurately evaluate significance for some analysis procedures for settings that have no known mathematical formula to accurately compute statistical significance. GSDA uses the formula derived by Zhu et al. [15] and therefore does not need to use resampling to compute *p* values. This dramatically reduces the computational burden of GSDA in practice. The *t*-test can eliminate non-significant gene-sets from consideration very quickly. The *t*-test approximation to the null distribution may not be accurate in the extreme tails in all applications. Therefore, the GSDA R package includes a module to perform a rapid permutation test to evaluate the accuracy of the most significant *p* values. We used permutation procedure in the example application below and recommend using it to follow-up significant distance correlation *t*-test *p* values.

GSDA also does not rely on fitting statistical models. This can be an advantage in some settings and a limitation in others. If the question of scientific interest requires evaluating the association of the gene-set with the variable of interest after adjusting for some other factor, then some methods that fit statistical models may be able to more rigorously and easily perform an adjusted analysis by simply including an adjustment term in the model. GT, POST and SAFE can incorporate covariate adjustments in their modeling frameworks. However, if the question does not require adjustment for another factor, then the statistical model fitting can introduce additional computational complexity that can be cumbersome. Fitting many statistical models requires numerically optimizing a likelihood or other criterion. In some data sets, the criterion may not have an optimum; for example, a monotone likelihood can occur when fitting Cox or logistic regression models to some data sets as described by Heinze and Shemper [25, 26]. The non-existance of an optimum leads to non-convergence of numerical optimization routines and can cause an analysis script to crash. In our experience in research of rare diseases with small sample sizes and relatively good outcomes, this occurs fairly often when fitting many statistical models with many different variables. The correlation and *t*-statistics of GSDA are computed by a series of simple arithmetic operations (addition, subtraction, multiplication, and division). Thus, non-convergence of model-fitting is not a concern. Division by zero is the only mathematically problematic situation that can arise with GSDA. These situations can be identified and avoided prospectively by flagging any gene-sets for which $(\tilde{{\varvec{X}}} \cdot \tilde{{\varvec{X}}}) = 0$ or $(\tilde{{\varvec{Y}}} \cdot \tilde{{\varvec{Y}}}) = 0$ and lead to division by zero in Eq. (3). These conditions can only occur in the setting that all distances are zero, which would not be a case of scientific interest in most settings anyway.

### Identifying the empirical drivers of an association

It can be difficult to provide a meaningful biological interpretation of a significant association of a gene-set with a treatment or outcome variable because the significant association may exist only for a *subset* of the gene-set. The *t*-test can be rapidly computed so it is feasible to incorporate it into a backwards elimination procedure to identify a subset of genes that are the empirical drivers of a significant association result. The procedure first computes the *t*-test *p* value for the gene-set with each gene excluded and identifies which gene to eliminate to yield the smallest *p* value. This procedure is then repeated until only one variable remains. This procedure gives a series of subsets of the original gene-set and the GSDA *p* value for each subset. The subset with the best GSDA *p* value may then be considered the empirical drivers of the significant association result and evaluated more carefully in follow-up laboratory research.

When interpreting the results of this exploratory follow-up analysis, it is important to recall that the classical interpretation of the *p* value for the selected subset may not hold because the hypothesis was not pre-specified. In this follow-up analysis, the *p* value of the best subset should be viewed simply as a subset selection criterion, not a rigorous Type I error control metric. Nevertheless, the procedure can help identify the most important genes within a gene-set. When strict Type I error control is necessary, one may embed this backwards elimination procedure within a permutation testing framework.

**Fig. 2:**
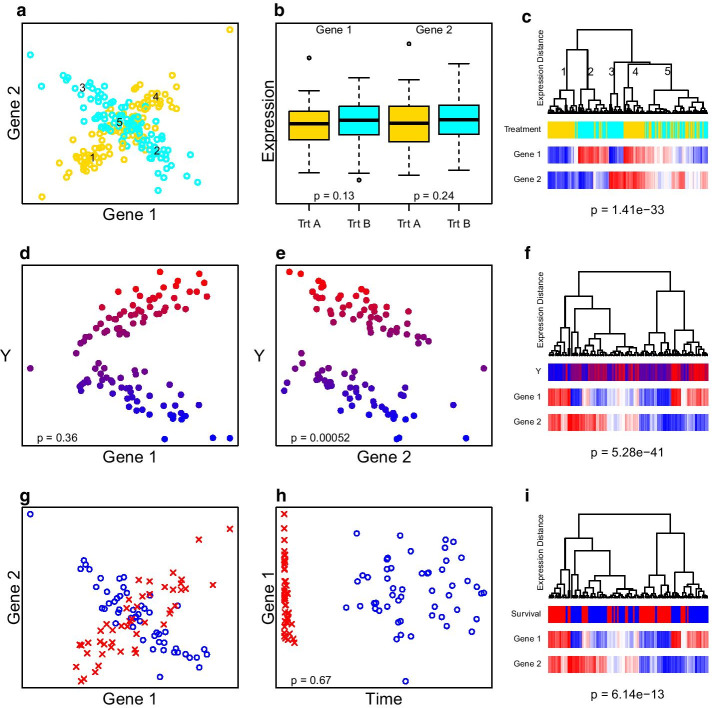
Simulated examples of associations more easily detected by GSDA than by other methods. **a** A scatterplot of the expression of two genes colored cyan or gold by the value of a categorical variable. **b** Boxplots for each of these two genes by category level with the Wilcoxon rank-sum test *p* value for each gene (*p* = 0.13 and 0.24 for genes 1 and 2, respectively). **c** A dendrogram for hierarchical clustering of these two genes on Euclidean distance on the expression data by Ward’s criteria with a color bar for the categorical variable value (GSDA *t*-test *p* = $1.41 \times 10^{-33}$, permutation $p< 10^{-6}$). Five branches of the dendrogram are numerically indexed and the means of those five branches are shown by the positions of the numeric indices in **a**. **d** A scatterplot of a numeric variable Y and the expression of gene 1 (Spearman $p = 0.36$). The numeric variable Y is also indicated by a blue–purple–red color scale. **e** A scatterplot of a numeric variable Y and the expression of gene 2 (Spearman $p = 0.00052$). **f** Dendrogram for hierarhical clustering on genes 1 and 2 by Ward’s criteria with a blue–purple–red color bar for the numeric variable Y (GSDA $p = 5.28 \times 10^{-41}$, permutation $p < 10^{-6}$). **g** A scatterplot of the expression of genes 1 and 2 with plotting character showing event status (red x = event, blue o = censored). **h** A scatterplot of gene 1 expression versus observation time with the same plotting characters showing event status (Cox regression $p = 0.87$). **i** A dendrogram for hierarchical clustering of the expression of genes 1 and 2 by Ward’s criteria with a color bar indicating event status (blue indicates event before year 4, gray indicates censor before year 4, red indicates event after year 4; GSDA *t*-test $p = 6.1 \times 10^{-13}$, permutation $p = 0.000111$)

### Illustrative examples

The primary statistical advantage of GSDA is its ability to detect complex forms of association that are not detectable by most other methods. GSDA can detect non-monotone relationships of one or more genes with a categorical treatment or trait, a quantitative trait, or a censored event-time endpoint. Figure 2 gives a few simulated examples of complex associations that many other methods have difficulty detecting as statistically significant. Each of these illustrative examples is described in greater detail below.

Figure 2a–c illustrate a setting in which two genes have differential *correlation* (but equal mean expression) across two categories. Hu et al. [27] described differential correlation of several pairs of genes between two subtypes of acute lymphoblastic leukemia. Nettleton et al. [12] also described a differential correlation of a gene-set with knock-out of myostatin in a comparison of myostatin-knockout and wild type mice. Figure 2a clearly shows that expression of the two genes are positively correlated in the category represented by gold dots and are negatively correlated in the category represented by cyan dots. Figure 2b shows that neither gene has differential median expression across the two categories according to the Wilcoxon rank sum test. Figure 2c shows that GSDA finds that this gene-set of two genes is very significantly associated with the category (GSDA *t*-test $p = 1.41 \times 10^{-33}$, permutation p $< 10^{-6}$). Five major clades of the dendrogram are numerically indexed in Fig. 2c and the mean position of these five clades is indicated in Fig. 2c. Four of the five clades correspond to groups that have low or high expression for each of the two genes ($2 \times 2 = 4$ and the fifth clade corresponds to a group with intermediate expression for both genes. The distance correlation test identifies this association.

Figure 2d–f illustrate a setting in which both genes have an association with a numeric variable *Y* that is more complex than what is represented by simple linear models. Figure 2d shows a V-shaped scatterplot for the numeric variable *Y* and the expression of gene 1. Figure 2e shows a scatterplot of the numeric variable *Y* versus the expression of gene 2 in which the points fall along two parallel lines with negative slope. From Fig. 2e, f, it is apparent that the association of *Y* and gene 1 depends on the expression of gene 2. *Y* and gene 1 are positively correlated when gene 2 is highly expressed and they are negatively correlated when gene 2 is lowly expressed. This is an example of a *liquid association* described by Ho [28] which they discovered among several triplets of genes with liquid associations in a cell-cycle experiment. In our illustrative example, Spearman’s test does not give a significant result for gene 1 (Fig. 2d, $p = 0.36$) but gets a significant result for gene 2 (Fig. 2e, $p = 0.00052$). Figure 2f shows that GSDA gives a very significant result for this two-gene gene-set ($p = 5.28 \times 10^{-41}$, permutation $p < 10^{-6}$). The color bar for the numeric phenotype *Y* in Fig. 2f shows a clear association with the clades of the dendrogram based on gene expression distance.

Figure 2g–i illustrate an association between two genes and a censored survival time endpoint that is more complex than what is represented by simple Cox regression models. Figure 2g shows a scatterplot of the expression of genes 1 and 2 and indicates the survival status by the plotting character (x = dead; o = alive). It is clear that these genes are negatively correlated in those patients who survived longer and positively correlated in those patients who died relatively quickly. This is another example of a liquid association. Figure 2h plots the expression of gene 1 versus the survival time and indicates survival status by the plotting character. A single-predictor Cox regression model does not find a significant association for gene 1 ($p = 0.67$, Fig. 2h) or gene 2 ($p = 0.83$, data not shown). GSDA finds this complex association to be statistically significant (Figure 2I, *t*-test $p = 6.1 \times 10^{-13}$, permutation $p = 0.000111$). Again, the color bar for survival outcome shows differences across the clades of the dendrogram computed by gene expression distance.

These illustrative examples show some complex forms of associations that violate the assumptions of the classical statistical methods such as the t-test, linear regression, Spearman correlation, logistic regression, Cox regression, etc that are utilized by widely used gene-set association testing methods such as SAFE, GT, and GSEA. These classical statistical methods are very powerful for detecting associations that are linear or monotone on some scale. The examples above illustrate some complex associations that have X-shaped, V-shaped, or other patterns that are not accurately modeled by a monotonic relationship. These complex associations may occur when there are unrecognized latent subgroups in the analysis. This may be the case for some complex human diseases, particularly human malignancies, in which unrecognized molecular subgroups may be present and impact the association. These non-monotonic relationships are still apparent in distance correlations and may be detected by GSDA.

## Results

### Simulation studies

We performed a series of simulation studies to evaluate the performance of the proposed GSDA method, GSEA, GSA, SAFE, GT, TOTS, and POST in simple settings involving a categorical, numeric, and survival outcome (SC, SN, and SS), complex settings involving categorical, numeric, and survival outcome (CC, CN, and CS). We also evaluated each of these settings with two different collections of gene sets. For gene set collection A, there were 100 genes (10 associated, 90 null) assigned into 60 gene sets with 8–10 genes each; 10 gene sets included at least one gene associated with the outcome and the other 50 gene sets had no gene associated with the outcome. For gene set collection B, there were 1000 genes (10 associated, 990 null) assigned into 100 gene sets with 10–100 genes each; 20 gene sets had at least one gene associated with the outcome and 80 gene sets had no gene associated with the outcome. We evaluated sample sizes of 10, 25, 50, and 100 subjects in each of two groups for categorical associations. For numeric and survival endpoints, we evaluated sample sizes of 10, 25, 50, and 100 subjects total. We evaluated the level and power of each method at the $p=0.05$ threshold.

Data were generated so that genes had no association, simple associations, or complex associations with the endpoints. For genes with no association, expression values were independently and identically distributed (iid) standard normal values. A latent numeric variable was used in the generation of data for endpoints and associated genes. Endpoint data were generated as simple functions of a latent vector of iid standard normal values and some additional random variation. For genes with a simple association with endpoints, expression values were generated by multiplying the latent variable by a coefficient and then adding iid standard normal errors. For genes with a complex association with endpoints, a latent binary vector was generated and used to reverse the signs of the coefficients for multiplication by the numeric latent variable. In the statistical literature, latent variables are unobserved variables that are used to more effectively model or simulate complex association settings. The reversal of signs by a latent binary vector in these simulations mimics the differential correlations described in “Illustrative examples” section and shown in Fig. 2a. These association patterns are similar to the complex differential expression of nucleotidyltransferase activity genes between myostatin knock-out and wildtype mice described by Nettleton et al. [12] and the liquid associations among multiple gene-triplets that Ho et al. [28] discovered in cell cycle data. Briefly, the expression correlation for a pair of genes depends on the expression of a third gene. For example, the expressions of genes A and B ar positively correlated when gene C is underexpressed and the expression of genes A and B are negatively correlated when gene C is overexpressed. The Additional file 1: simulation-structure.pdf provides a detailed narrative description of simulation with illustrative schema figures.

**Fig. 3:**
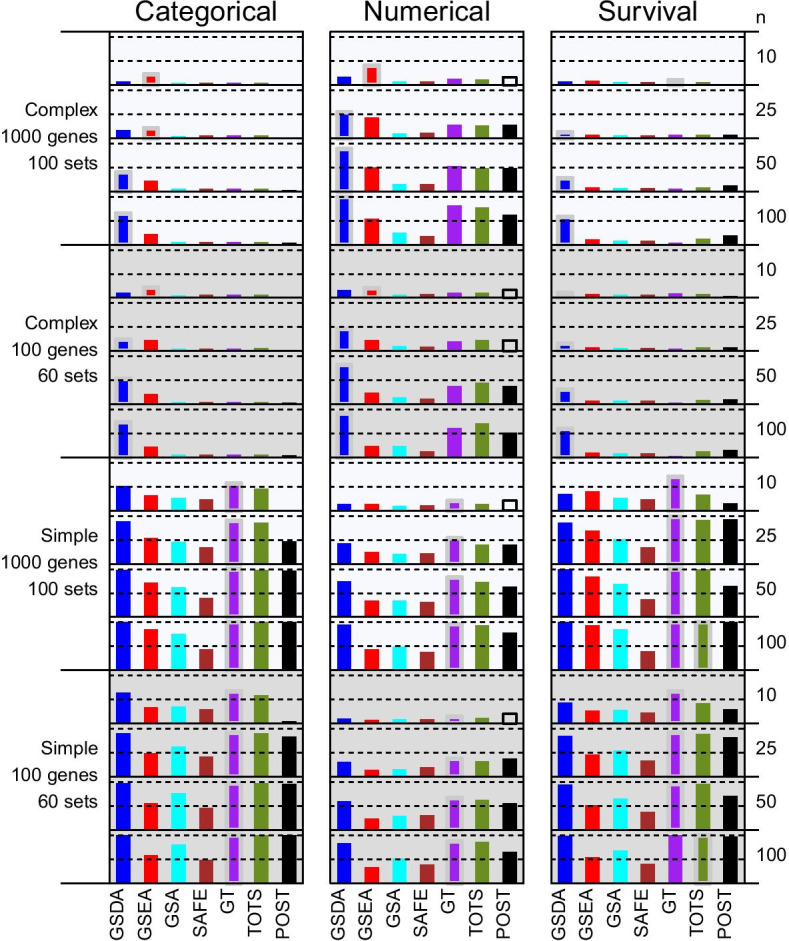
Simulation results. Each panel in the above figure shows a bar plot of the power of the seven methods in one simulation setting. The 48 settings evaluate three different enpoints as indicated by the column headers with simple or complex associations in 100 or 1000 genes (in blocks of rows with gray or azure backgrounds) as indicated by the labels on the left for sample size $n=10$, $n=20$, $n=50$, or $n=100$ as indicated by the labels on the right. Each bar represents the power of one method; the colors for the methods are indicated by the legend at the bottom. A filled bar indicates that the empirical level was less than 6% and a hollow bar indicates that the empirical level was greater than 6%. All methods were performed with a nominal level of 5%. In each panel, a gold border around a bar indicates that the method had the best power or was within 1% of the best power and kept its empirical level less than 6%. Each panel includes dashed horizontal lines at 50% and 100% power

Figure 3 shows each method’s average level over all null gene sets and average power over all associated gene sets in each simulation setting. All methods maintained Type I error controlless than 6% except that POST failed to do so in a few small sample size settings. For each setting, the method with the greatest power among those methods with average empirical level less than 6% was designated as the best performer. GT was the best performer in 23 of 24 simple association settings and one of the 24 simple association settings. GSEA was the best performer in five of 24 complex association settings; those five settings had small sample sizes (10 or 25). TOTS was the best performer in the simple survival association setting with100 genes and 60 gene-set and a sample size 100. In that setting, POST, GT and GSDA had only slightly less power. GSDA was the best performer in 18 of 24 complex association settings; those settings had sample sizes of 10 to 100. In 10 of these 18 settings, the power of GSDA exceeded that of all other methods by 20% or more. In complex survival association settings with $n=100$ subjects, the power of GSDA exceeded that of all other methods by at least 39%. Also, the power of GSDA was similar to that of GT in most of the simple association settings. Complete simulation results are provided in the Additional file 3: simulation-boxplots.pdf and Additional file 4: simulation-results.xlsx.

We also used the simulation results to more fully understand the statistical properties of the GSDA distance correlation *t*-test *p* values by examining empirical distribution function (EDF) plots of those *p* values. The Additional file 2: simulation-EDF-plots.pdf includes *p* value EDF plots for each simulation. In all cases, the GSDA *p* values of associated gene-sets show a much greater concentration near zero than do those of null gene-sets. This observation is consistent with GSDA maintaining level and increasing power with sample size in the results reported above. Additionally, consistent with the theoretical derivations of Zhu et al. [15], the GSDA *p* values for null gene sets are approximately uniform over most of the *p* value range from 0 to 1. The GSDA *p* values for null gene sets tend to be slightly stochastically greater than uniform for $p> 0.04$ and slightly stochastically less than uniform for $p< 0.04$. This suggests that the GSDA distance correlation *t*-test of Eq. (4) may tend to overstate statistical significance when it gives $p< 0.04$. Therefore, we recommend using the permutation procedure of “Verifying significant results with permutation” section to evaluate the accuracy of significant distance correlation *t*-test results as we have done in the illustrative examples of “2.11” section above and in the example application immediately below.

Additionally, we measured the computing times for each method in our simulation (Additional file 5: compute-speeds.pdf). GT was one of the two fastest methods in all 48 simulation settings. GSDA was one of the three fastest methods in all 32 settings involving a numerical or categorical endpoint. GSDA was noticeably slower in evaluating survival endpoints. Computing the survival distnace in Eq. (6) can be time consuming because it must perform $O(n^3)$ comparisons (compare data for all $n(n-1)$ pairs at each of *n* timepoints). Nevertheless, it is still computationally feasible to perform GSDA in practice; the superior statistical perfomance of GSDA is well worth the additional computing time.

### A pediatric leukemia application

We applied these seven analysis methods to the pediatric acute myeloid leukemia (AML) data set that is publicly available from the TARGET project website (https://target-data.nci.nih.gov/Public/AML/; accessed March 8, 2018). We also applied all eleven methods of the R package *piano* [14] to the data set. We evaluated the association of gene-level mRNA-seq expression data with presence/absence of chloroma at diagnosis (a binary variable), log white blood cell count (logWBC) at diagnosis (a quantitative variable), and event-free survival (a censored event time variable) for the 123 subjects with all of these data available. We used these 18 methods to evaluate the association of the expression of 56 genes annotated to the KEGG AML pathway (https://www.genome.jp/kegg-bin/show_pathway?hsa05221) with the presence or absence of chloroma at diagnosis (a binary variable), the log of the diagnostic white blood cell count (a numeric variable), and event-free survival of patients (a censored event time variable). We also used the procedure of “Verifying significant results with permutation” section to compute GSDA *p* values based on one million permutations.

**Table 2:** Results for of the pediatric AML analysis

Method	Chloroma	logWBC	EFS
GSDA (t-test)	0.020	$< 10^{-29}$	0.060
GSDA (perm)	0.029	$<10^{-6}$	0.051
GSEA	0.030	0.008	0.572
GSA	0.512	0.688	0.114
SAFE	0.561	0.014	0.336
GT	0.014	$< 10^{-5}$	0.250
TOTS	0.960	$< 0.001$	0.217
POST	0.027	$< 10^{-5}$	0.130
PIANO (Wilcoxon)*	0.804	0.004	0.209
PIANO (Fisher)*	0.0004	$< 10^{-37}$	0.092
PIANO (Stouffer)*	0.004	$< 10^{-27}$	0.164
PIANO (Reporter)*	0.742	0.004	0.288
PIANO (Tail Strength)*	0.744	0.016	0.239
PIANO (Mean)*	0.836	0.030	0.217
PIANO (Median)*	0.834	0.001	0.316
PIANO (Sum)*	0.858	0.042	0.221
PIANO (MaxMean)*	0.116	0.011	1.000
PIANO (GSEA)*	0.039	0.028	1.000
PIANO (FGSEA)*	0.014	0.029	1.000
PIANO (PAGE)*	0.044	0.096	0.112

The table shows the *p* values for each method for the association the expression of the KEGG AML pathway with clinical characteristics and survival in pediatric AML

*PIANO was performed with only the gene-level statistics with *signifMethod=’nullDist’* for Wilcoxon, Fisher, Stouffer, and Reporter methods and *signifMethod = ‘geneSampling’* and 999 permutations for the other methods

Table 2 provides the results. GSDA and 8 other methods found that the KEGG AML pathway was significantly associated with chloroma ($p < 0.04$); the other 10 methods did not find strong evidence of an association ($p > 0.11$ ). The AML pathway genes were not significantly associated with WBC according to GSA ($p = 0.688$) or the PAGE method of PIANO ($p=0.096$), but was significantly associated according to GSDA ($p < 10^{-6}$) and all other methods ($p \le 0.03$). GSDA found that the KEGG AML pathway had a marginally significant association with EFS (*t*-test $p = 0.06$, permutation $p = 0.050755$); all other methods obtained $p > 0.09$ for this association. GSDA obtaining the smallest *p* value for EFS is consistent with the simulation result showing that GSDA has unrivaled power to detect a complex association of a gene-set with a censored survival time variable with sample size of 100 (Fig. 3).

**Fig. 4:**
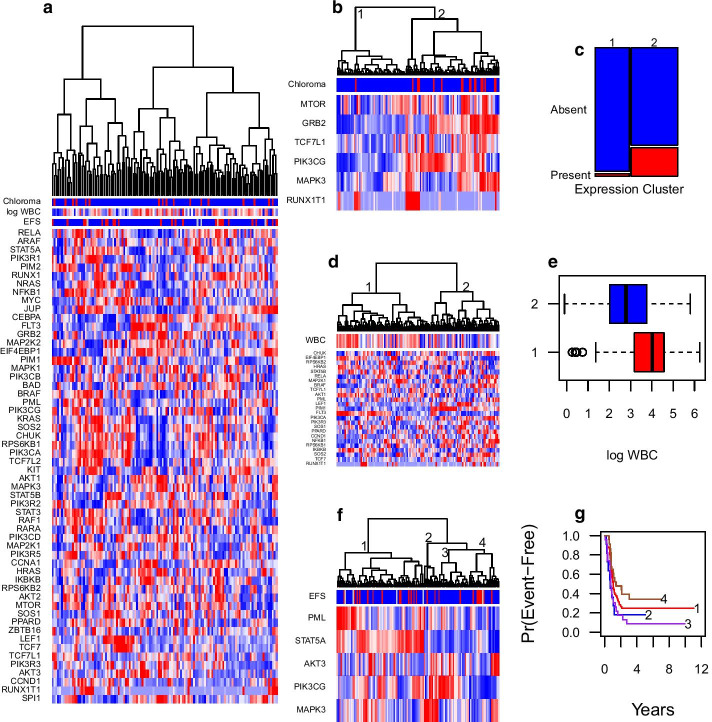
Results for the pediatric AML example. **a** The data for the three phenotypes as color bars and expression for all the KEGG AML pathway genes as a heatmap. The dendrogram is obtained by Euclidean distance clustering with Ward’s criteria on the expression data. In the color bar for chloroma, red indicates chloroma is present and blue indicates that chloroma is absent. The color bar for log WBC shows lower counts as blue and greater counts as red. The color bar for EFS shows events before year 4 as blue, censoring before year 4 as gray, and event after 4 years as red. **b** A color bar for chloroma and a heatmap of expression for the six genes obtained by the backward selection procedure of “Identifying the empirical drivers of an association” section. The dendrogram was determined by clustering on Euclidean distance on the expression of those six genes. Two branches of the dendrogram are numerically indexed. **c** A mosaicplot of chloroma and expression subgroup defined by the dendrogram in **b**. It shows that 16 of 17 choloroma cases belong to expression subgroup 2. **d** The color bar for log WBC and the heatmap of expression for associated genes as determined by the backward selection procedure. Two branches of the dendrogram are numerically indexed. **e** A boxplot of log WBC by expression subgroup defined in **d**. **f** A color bar of EFS and heatmap of expression for the 5 genes chosen by backward selection. Four branches of the dendrogram are numerically indexed. **g** Kaplan–Meier estimates of EFS for the four subgroups defined in **f**

Figure 4 graphically illustrates the results of GSDA and the backward elimination procedure of “Identifying the empirical drivers of an association” section. Figure 4a provides a heatmap of the expression of the AML pathway genes and a color scales for each of three endpoints. The backward elimination procedure identified RUNX1T1, MAPK3, PIK3CG, TCF7L1, GRB2, and MTOR as the empirical drivers of the association with chloroma (Fig. 4b). Hierarchical clustering of individuals by the expression of these six genes with Ward’s criteria [29] defines one cluster of 52 patients with only one of the 17 chloroma cases (Fig. 4c). The backward elimination procedure identified 25 of the 56 genes as empirical drivers of the association with logWBC (Fig. 4d). Hierarchical clustering of individuals by the expression of these 25 genes defined two subgroups with strong differential logWBC values (Fig. 4). By backward elimination, AKT3, MAPK3, PIK3CG, PML, and STAT5A were identified as empirical drivers of the association with EFS (Fig. 4f). Again, hierarchical clustering of subjects by these genes defined subgroups with differing EFS (Fig. 4g). We verified this result by using GSDA to test the association of these five genes with EFS in the entirely separate AML02 clinical trial cohort of 168 patients [30, 31]. In this independent validation cohort, GSDA found this set of five genes to be significantly associated with EFS (*t*-test $p = 0.013839$, permutation $p = 0.024695$).

In this example, only GSDA obtained $p < 0.09$ for the association with EFS in the TARGET AML cohort. There are several reasons the other methods did not find suggestive evidence of this association. Each of the other methods rely on Cox proportional hazards regression to model and evaluate the significance of the association. Fitting a single-predictor Cox regression to each gene in the gene-set yields a set of *p* values with a mean of 0.45. Pounds and Cheng [21] have shown that twice the average *p* value is a reasonable estimator of the proportion of tests with a true null hypothesis. Thus, an average Cox *p* value of 0.45 indicates that only 10% of the 56 genes are associated with EFS. This estimate is also consistent with the backwards elimination procedure identifying 5 genes as empirical drivers of the association with EFS. Additionally, we used the method of Grambsch and Therneau [32] to evaluate the validity of the proportional hazard assumption for each gene. The average *p* value was 0.41, indicating that 18% of genes in the gene set violate the assumption. The other methods did not find the association because a substantial proportion of genes violated the modeling assumption and only a small proportion were truly associated with EFS.

## Discussion

Many scientific discoveries have been made by identifying gene sets that associate with a treatment or an endpoint of interest. According to Google Scholar, the gene-set analysis methods evaluated in our simulation studies and example analysis have been cumulatively cited tens of thousands of times. Several methods have been successfully used for this purpose in the literature. Nevertheless, it is important to more fully understand the strengths and limitations of these methods in terms of recognizing biological settings for which each method has good or poor statistical performance. Our work has confirmed that many of the widely used methods perform well in certain settings, particularly in identifying gene sets for which most genes have a simple association with the endpoint or treatment of interest. Many times, these discoveries are scientifically meaningful; thus, the widely used methods can often reveal biological insights necessary to advance our understanding and treatment of several diseases.

However, as is the case for many statistical problems, each method has a niche of settings for which it performs well and for which it performs poorly. Nettleton et al. [12] observed that some complex forms of association that are essentially undetectable by some of the most widely used methods. Ho [28] also observed complex associations that are difficult to detect with methods that assume monotonic relationships between pairs of variables. Nettleton et al. [12] proposed the MRPP to evaluate the association of a gene set with a categorical endpoint or treatment variable. In addition to detecting differential average expression across groups, the MRPP can detect differential correlation across groups. However, the published MRPP did not evaluate the association of a gene set with a quantitative or censored survival time variable. In many disciplines, such as oncology, censored survival time variables are of greatest scientific interest.

Zhu et al. [15] developed a distance correlation *t*-test that can detect complex associations between two numeric data matrices. The method is appealing in that it can detect complex associations without resorting to computationally burdensome resampling procedures to determine statistical significance. However, in its published form, it is limited to evaluating associations between two numeric data matrices. We extended this framework to develop the GSDA method that can also evaluate associations of a numeric data matrix with categorical variables and censored event-time variables. GSDA was the best performer in 16 of 24 complex association settings and exceeded the power of all other methods by 20 percentage points in 11 of those settings. These results indicate that in many settings GSDA has much greater power than other methods to detect complex associations with a gene-set. In this way, GSDA can effectively make discoveries that complement the discoveries made by other gene-set testing methods.

We also developed methods to follow-up on the most statistically significant and/or biologically interesting results of a GSDA analysis. We developed a rapid permutation testing procedure for GSDA to confirm the statistical significance of distance correlation *t*-test. We used this procedure to confirm the accuracy of the significant distance correlation *t*-test in our example application and recommend using it in practice to follow-up on the most statistically significant distance correlation *t*-test results. We also developed a backward elimination procedure (“Identifying the empirical drivers of an association” section) to provide more focused biological insights by identifying the subset of genes in a gene set with the strongest distance correlation with the endpoint of scientific interest. This backward elimination procedure was very useful in the example application. It narrowed down the 56 genes in the AML gene set to a subset of 25 genes for association with chloroma, 6 genes for association with log WBC, and 5 genes for association with EFS. We were able to confirm the EFS association of the 5 genes in a separate clinical trial cohort.

Overall, the simulation and example application results have shown that GSDA is a useful tool to complement existing gene-set association testing methods. GSDA can identify complex associations that are characterized by non-monotonic relationships among pairs of variables or that violate other statistical modeling assumptions. These types of associations occur in practice and are difficult to detect with other methods. GSDA can rapidly complete the analysis of all genes and provide the rigor of a permutation test for the most significant results. Also, GSDA can provide a more focused biological insights by identifying the subset of genes in a gene set that most strongly associated with an endpoint of interest.

Future research should explore several intriguing open questions related to the use and improvement of GSDA. The use and development of GSDA may be substantially advanced by developing new distance measures and guidelines for their use in practice and considering different algorithms to find strongly associated subsets of gene sets that associate with the outcome or endpoint of scientific interest.

## Conclusions

We developed the gene-set distance analysis method and showed that it detects associations of gene-sets with phenotypes or treatments that are not easily identified by other methods. These results indicate that GSDA should compliment the use of other methods in data analysis practice to ensure that biologically meaningful associations are discovered that may otherwise be missed. Furthermore, our work suggests that future work should develop distnace-based methodologies for other problems and applications in the analysis of omics data.

## Supplementary information

**Additional file 1.** This supplementary file describes the detailed settings for simulation studyincluding gene set collection definition, association coefficients, simple/complex association and three types ofresponse variables.

**Additional file 2.** This supplementary file provides the empirical distribution function (EDF) plots ofGSDA simulation p values for each scenario.

**Additional file 3.** This supplementary file provides box plots of power and type I error rates at 5%nominal level for each scenario.

**Additional file 4.** This supplementary file contains one spreadsheet with data describing geneeffect sizes and gene-set memberships for each simulation setting and another spreadsheet with simulation resultsfor each gene-set by each method in each setting.

**Additional file 5.** This supplementary file contains bar plots of the compute speeds (number ofcompleted analyses per minute) for each method in each of the 48 scenarios.

## Data Availability

The AML data set used in the example application is from publicly available TARGET project with reformatting and available as rdata at https://github.com/xueyuancao/GSDA/tree/master/data; The source code is available at https://github.com/xueyuancao/GSDA/tree/master/R.

## Abbreviations

GSDA: Gene-set distance analysis
AML: Acute myeloid leukemia
EFS: Event-free survival
GT: Global test
TOTS: Total of test statistics
SAFE: The significance and function of expression
GSEA: Gene-set enrichment analysis
GSA: Gene-set association
MRPP: Multiresponse permutation procedure
POST: Projection onto orthogonal statistical tests
FDR: False discovery rate
EDF: Empirical distribution function
