# Supplementary material for: Gene-set distance analysis (GSDA): a powerful tool for gene-set association analysis
Source: BMC Bioinformatics. 2021 Apr 21;22:207. doi: 10.1186/s12859-021-04110-x (PMC8059024; doi:10.1186/s12859-021-04110-x)
Supplement: Supplementary file 1 — Additional file 1. This supplementary file describes the detailed settings for simulation studyincluding gene set collection definition, association coefficients, simple/complex association and three types ofresponse variables. [file 12859_2021_4110_MOESM1_ESM.pdf]

Description of Simulations for

Gene-Set Distance Associations (GSDA): A Powerful Tool for Gene-Set Association Analysis

by Xueyuan Cao and Stan Pounds

Gene-Set Collections and Gene Associaton Coefficients

Simulations generated data for two different collections of gene sets. The first collection assigned 100 genes into 60 gene sets with 8-10 genes per gene-set. Ten of these gene sets included at least one gene that was associated with the endpoint and the other 60 gene sets did not include any gene that was associated with the endpoint.

The second collection assigned 1000 genes into gene-sets of size 10, 25, 50, or 100 genes. Twenty of these gene-sets included at least one gene that was associated with the endpoint and the remaining 80 gene sets did not include any gene that was associated with the endpoint.

For both gene-set collections, the first 10 genes were associated with the endpoints. In the simple numeric and simple categorical settings, the association coefficients β for the first 10 genes were 1/8, 2/8, 3/8, 4/8, 5/8, -5/8, -4/8, -3/8, -2/8, and 1/8, respectively. For the other four settings, the association coefficients β for the first 10 genes were ½, 2/2, 3/2, 4/2, 5/2, -5/2, -4/2, -3/2, -2/2, and -1/2, respectively. The association coefficients were zero for all other genes, which indicated these genes were not associated with the endpoints.

The spreadsheet tabs *Gene.Set.Collection.A* and *Gene Set Collection B* of the supplementary Excel file *simulation-result-tables.xlsx* provide the association coefficients and gene set assignments for each gene in each gene set collection.

Calculation of Statistical Level and Power

In each simulation replication (generation and analysis of one data set), the level for an analysis method was computed as the proportion of null gene-sets with p-value less than 0.05 and the power was computed as the proportion of non-null gene-sets with p-value less than 0.05. For each simulation (set of all replications of a specific setting with a given sample size), the level was the average of the level across all replications and the power was the average of the power across all replications.

Simple Categorical (SC) Simulation

In the simple categorical (SC) simulation setting, data for n subjects in each of two groups was generated. The $m \times 2n$ expression matrix X was generated as $X = \beta\delta + \varepsilon$ where β was an $m \times 1$ vector of association coefficients, δ was a 1 x $2n$ vector of group differences (δ_i = +0.5 for subjects in group 1 and δ_i = -0.5 for subjects in group 2) and ε was an $m \times 2n$ matrix of *iid* N(0,1) errors. Here, the term $\beta\delta$ represents the matrix product of β and δ. The elements of the association vector β defined the effect size for each gene. Each gene g with β_g=0 was equally expressed across the two groups; genes with $\beta_g \neq$

0 were differentially expressed by β_g standard deviations across groups. Supplementary Figure S1 shows this simulation schema.

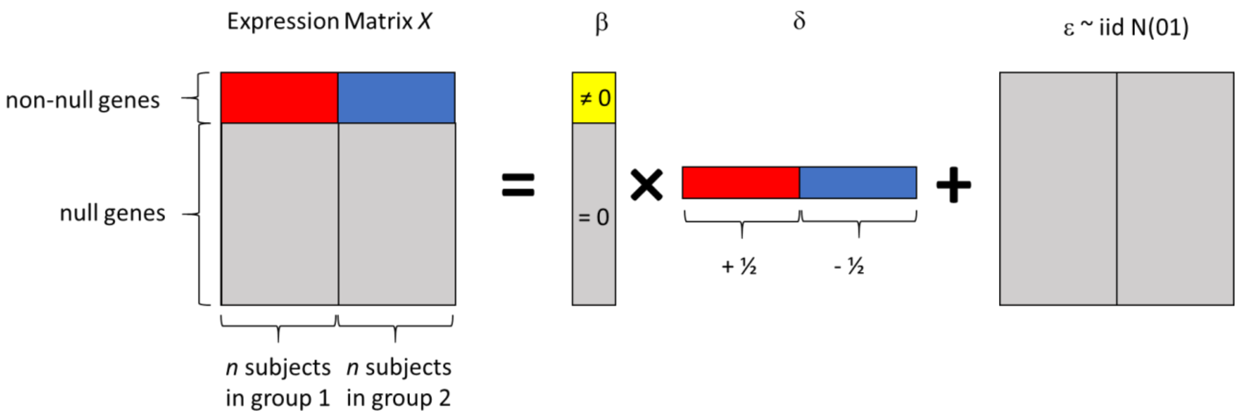

Figure S1. Simple categorical (SC) simulation schema.

Complex Categorical (CC) Simulation

In the complex categorical simulation, data for *n* subjects of each of two groups was generated. The $m \times 2n$ expression matrix X was generated as $X = \beta * z + \varepsilon$ where β is an m x 2n matrix of association coefficients, z is a 1 x2n latent variable vector of iid N(0,1) values, and ε is an m x 2n matrix of iid N(0,1) errors. Here, $\beta * z$ represents elementwise multiplication of each row of β with the latent variable vector z. The rows of β for null genes were all zero. For non-null genes, the odd rows of β had a value of +b/2 for group 1 and -b/2 for group 2 and the even rows of β had value -b/2 for all subjects, where b was the effect size vector described in the gene set association coefficients described above. Supplementary Figure S2 shows this simulation schema.

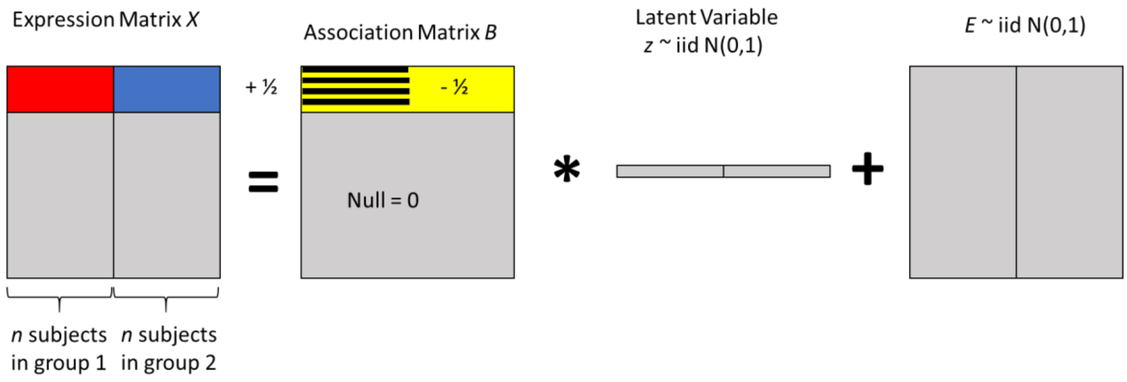

Figure S2. Complex categorical (CC) simulation schema.

Simple Numerical (SN) Simulation

The simple numeric simulation generated data for n subjects total. The 1 x n numeric outcome vector y was generated as a series of n iid N(0,1) observations. Given the n x 1 association coefficient vector β, the expression matrix was computed as $X = \beta y + \varepsilon$, where ε was an m x n matrix of iid N(0,1) errors. This simulation schema is shown in supplementary Figure S3.

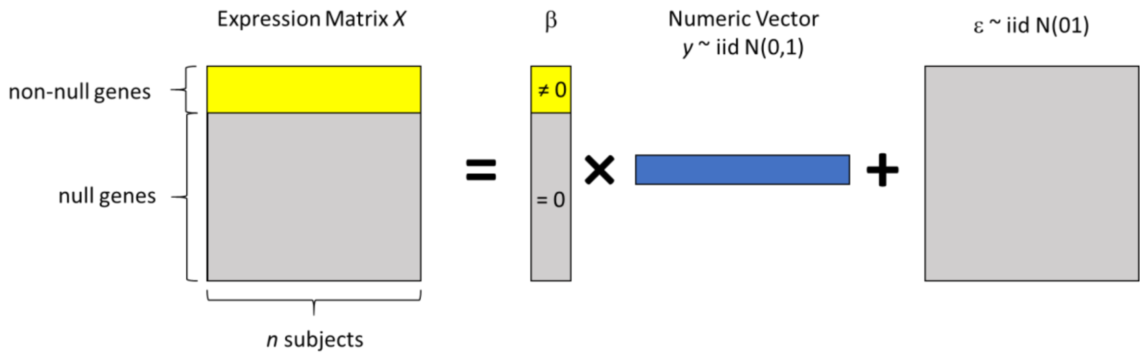

Figure S3. Simple numeric (SN) simulation schema.

Complex Numerical (CN) Simulation

The complex numerical simulation generated data for n subject as follows. The 1 x n numeric outcome variable y was generated as a series of n iid N(0,1) observations. The expression data matrix was computed as X = M + e where M = (βy)*L, where β is a given 1 x n association coefficient vector, L is a latent subgroup matrix for which the even rows have the same set of n observations randomly chosen from ± 1 with replacement (the expression of even- indexed genes are subject to two latent subgroups) and the odd rows are all 1 (the expression of odd-indexed genes are not subject to the latent subgroups), βy indicates the matrix product of β and y, and * indicates elementwise multiplication of two matrices of the same dimension. Supplementary Figure S4 shows the simulation schema for complex numeric associations.

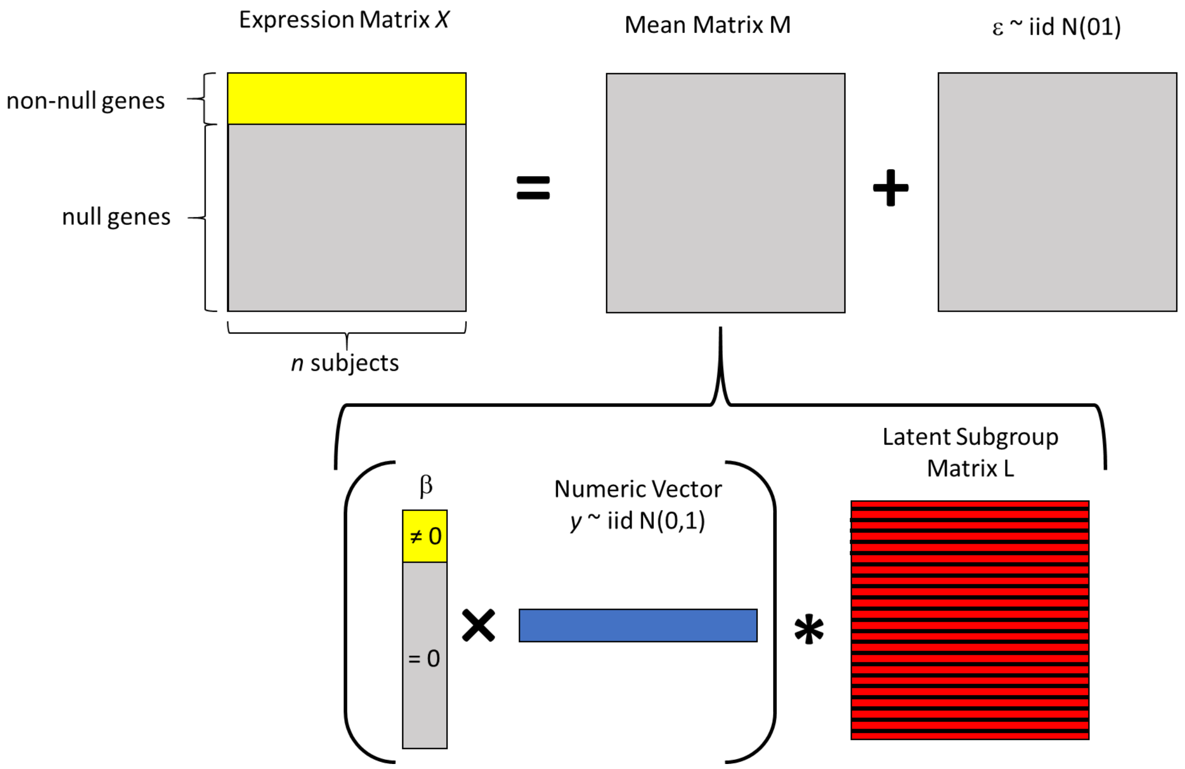

Figure S4. Simulation schema for complex numerical associations.

Simple Survival (SS) Association Simulation Schema

The simple survival association simulation generated data for n subjects as follows. A latent variable n-vector z was generated as a series of iid N(0,1) observations. Given an n x 1 association coefficient vector β and this 1 x n vector z, the m x n expression data matrix was generated as $X = \beta z + \varepsilon$, where ε was an m x n matrix of iid N(0,1) errors. The latent variable z was also used in the generation of survival time data so that each gene with $\beta \neq 0$ was associated with survival times. First, a cure indicator n-vector was generated as a series of independent Bernoulli observations where the success probability for each subject was $\Phi(2z)$, i.e., the probit function evaluated at $2z$. The complete survival time for cured subjects was defined as $+\infty$ and the survival time for other subjects were generated as independent observations from an exponential distribution with rate $-z$. Censoring times were generated as iid uniform(3,10) observations. For each subject, the observed survival time was the minimum of the complete survival time and the censoring time. The event status was an indicator of whether the complete survival time was less than or equal to the censoring time. Supplementary Figure S5 shows the simulation schema for simple survival associations.

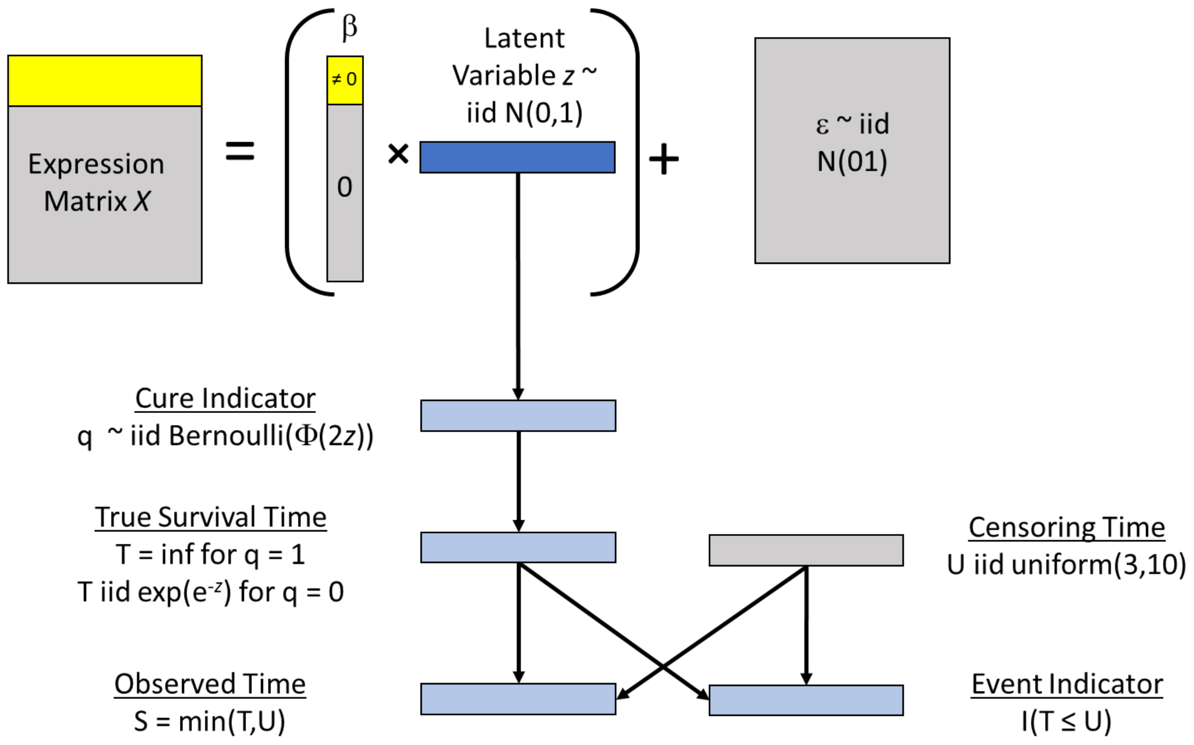

Figure S5: Simple Survival Association Simulation Schema

Complex Survival (CS) Association Simulation Schema

Data sets with complex survival associations were generated as follows. A numeric latent variable n-vector z and a latent subgroup n-vector ℓ were generated. The entries of the latent numeric vector z were generated as a series of iid N(0,1) observations. The entries of the latent subgroup vector ℓ were generated by randomly sampling from (-1,1) with replacement. A latent matrix L includes this latent subgroup vector on its odds rows and has all ones on its even rows. The m x n expression matrix X is then generated as X= $(\beta z)*L + \varepsilon$, where ε is an m x n matrix of iid N(0,1) errors. The numeric latent variable z and latent subgroup variable ℓ were also used in the generation of survival time data so that each gene with b ne 0 was associated with survival time. A cure indicator vector q was generated as a series of independent Bernoulli observations where each subject's probability of cure was the probit of $2z + 5\ell$. The complete survival time was infinite for cured subjects and was generated as a series of independent exponential observations such that the rate for each subject was $\exp(-z-3\ell)$. Censoring times were generated as iid uniform(3,10) observations. For each subject, the observed survival time was the minimum of the censoring and complete survival time and the event indicator was one if the complete survival time was less than or equal to the censoring time and zero if the censoring time was less than the complete survival time. Supplementary Figure S6 shows the simulation schema.

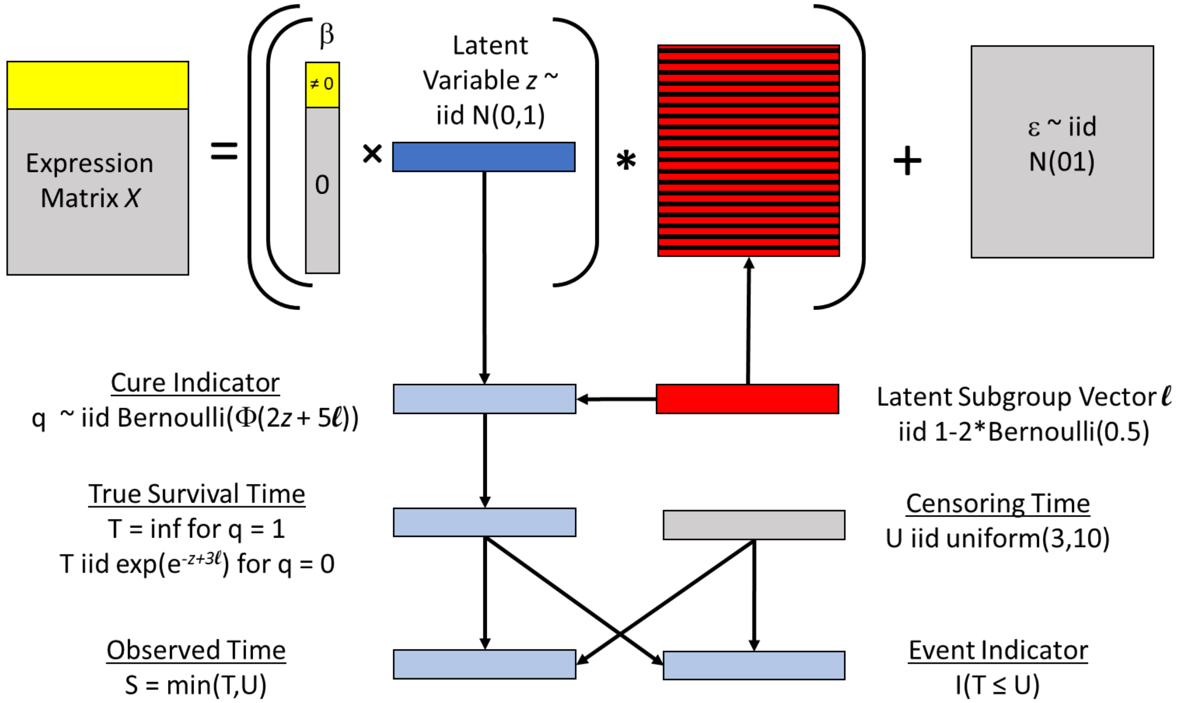

Figure S6. Complex Survival (CS) Association Simulation Schema
