# Supplementary material for: Gene-set distance analysis (GSDA): a powerful tool for gene-set association analysis
Source: BMC Bioinformatics. 2021 Apr 21;22:207. doi: 10.1186/s12859-021-04110-x (PMC8059024; doi:10.1186/s12859-021-04110-x)
Supplement: Supplementary file 2 — Additional file 2. This supplementary file provides the empirical distribution function (EDF) plots ofGSDA simulation p values for each scenario. [file 12859_2021_4110_MOESM2_ESM.pdf]

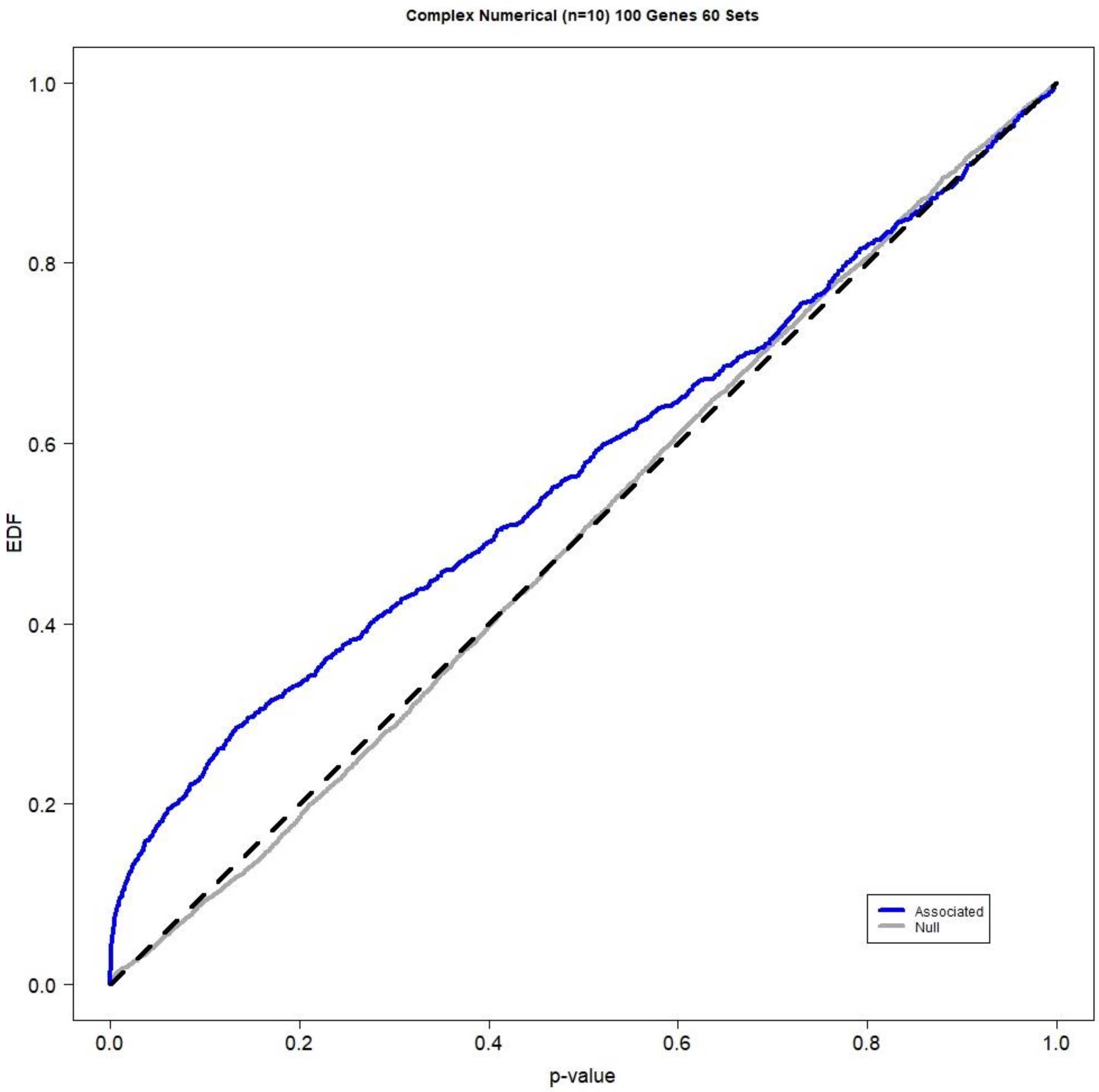

Complex Numerical (n=10) 100 Genes 60 Sets
EDF
p-value
0.0
0.2
0.4
0.6
0.8
1.0
Associated
Null

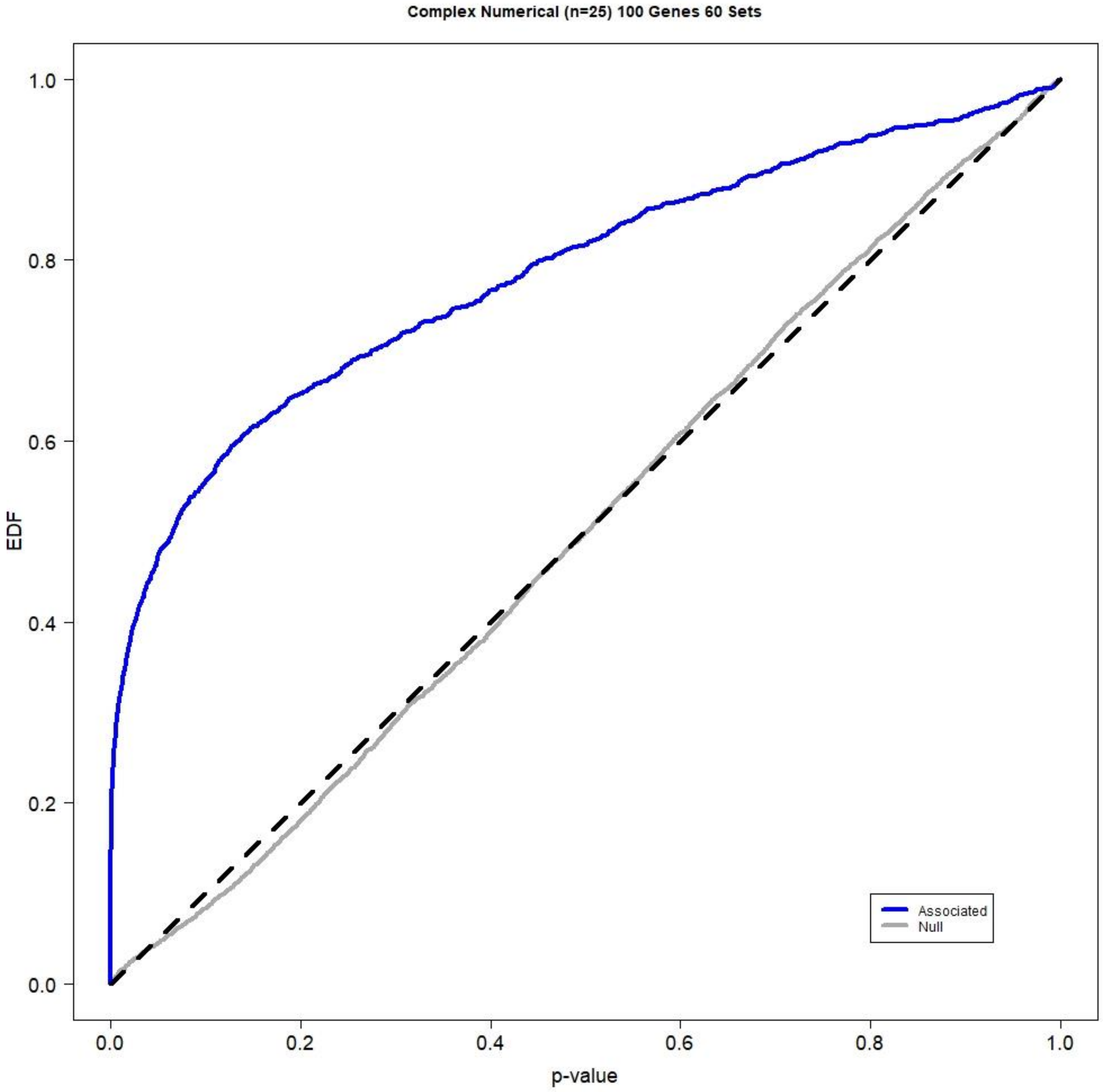
Complex Numerical (n=25) 100 Genes 60 Sets
EDF
p-value
0.0
0.2
0.4
0.6
0.8
1.0
0.0
0.2
0.4
0.6
0.8
1.0
Associated
Null

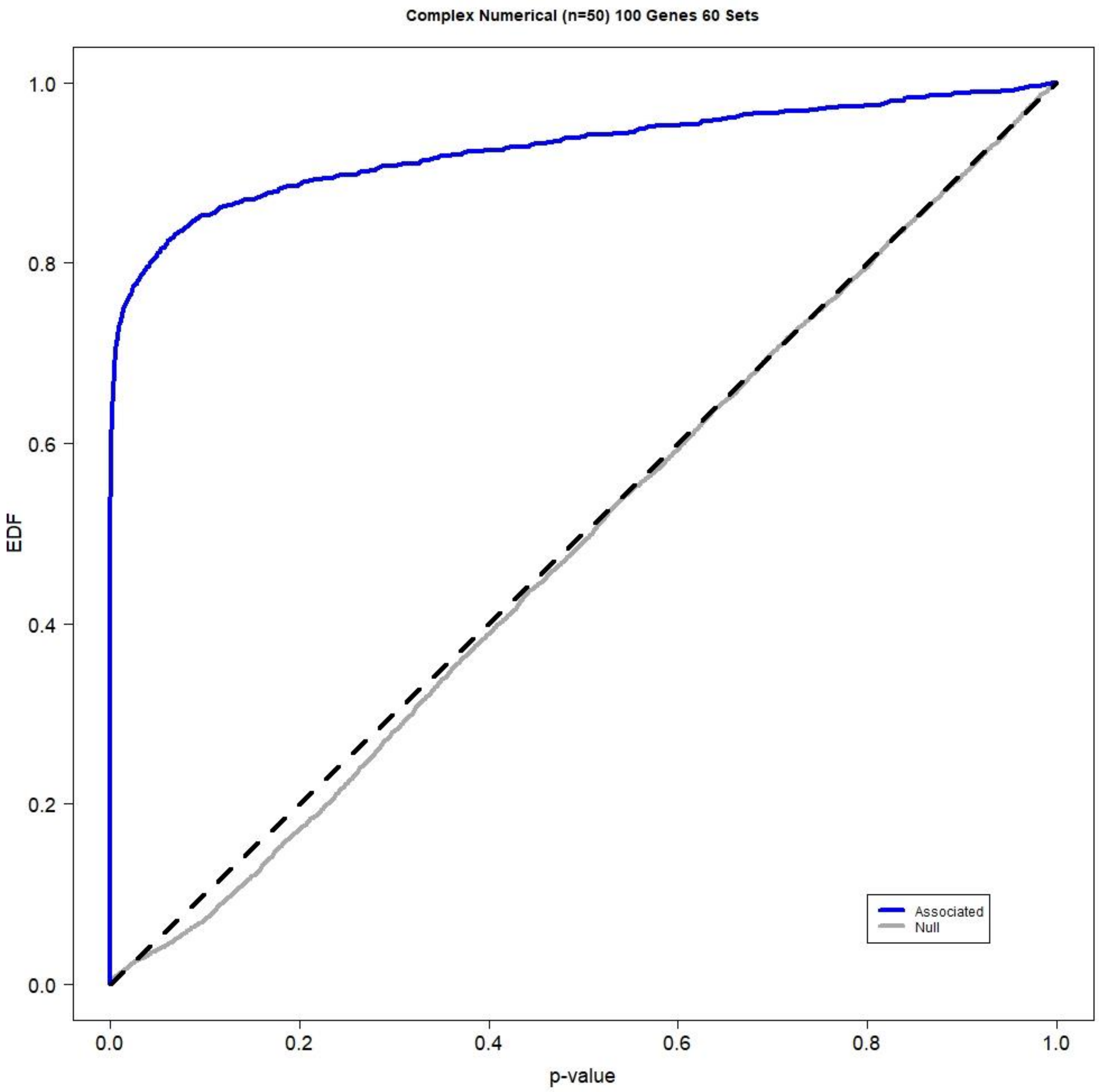

Complex Numerical (n=50) 100 Genes 60 Sets
EDF
p-value
0.0
0.2
0.4
0.6
0.8
1.0
Associated
Null

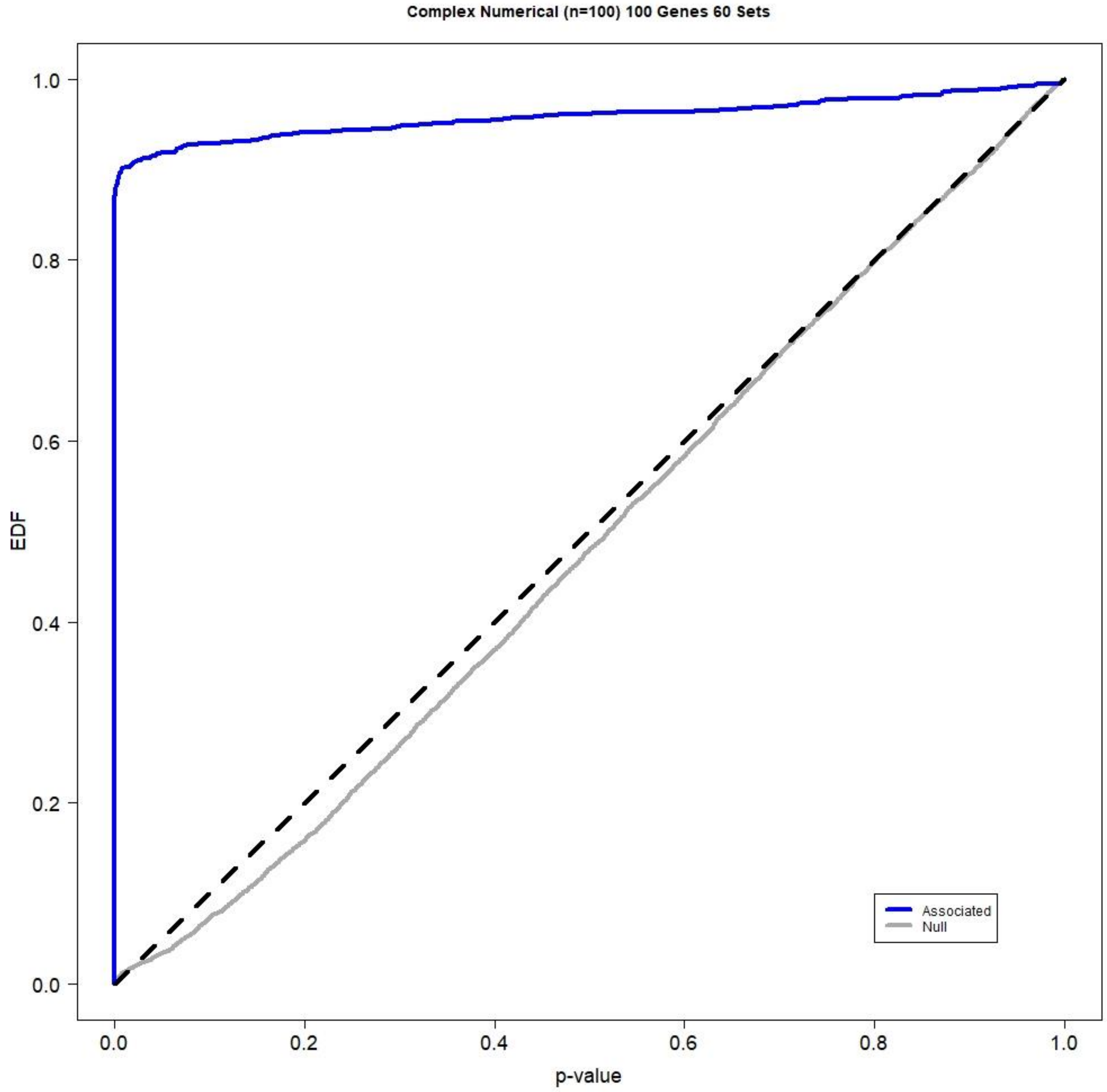
Complex Numerical (n=100) 100 Genes 60 Sets
EDF
p-value
0.0
0.2
0.4
0.6
0.8
1.0
Associated
Null

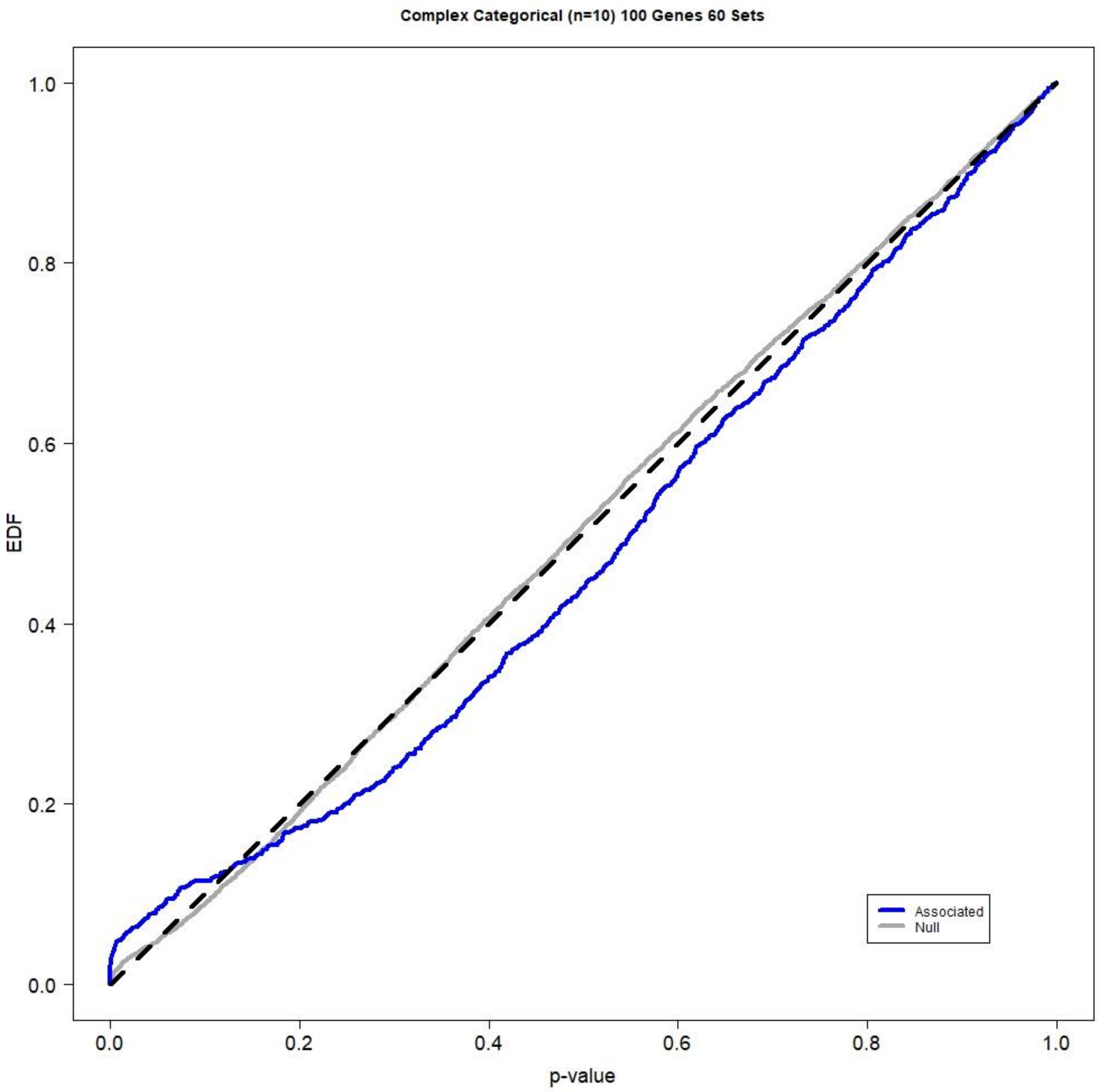

Complex Categorical (n=10) 100 Genes 60 Sets
EDF
p-value
0.0
0.2
0.4
0.6
0.8
1.0
0.0
0.2
0.4
0.6
0.8
1.0
Associated
Null

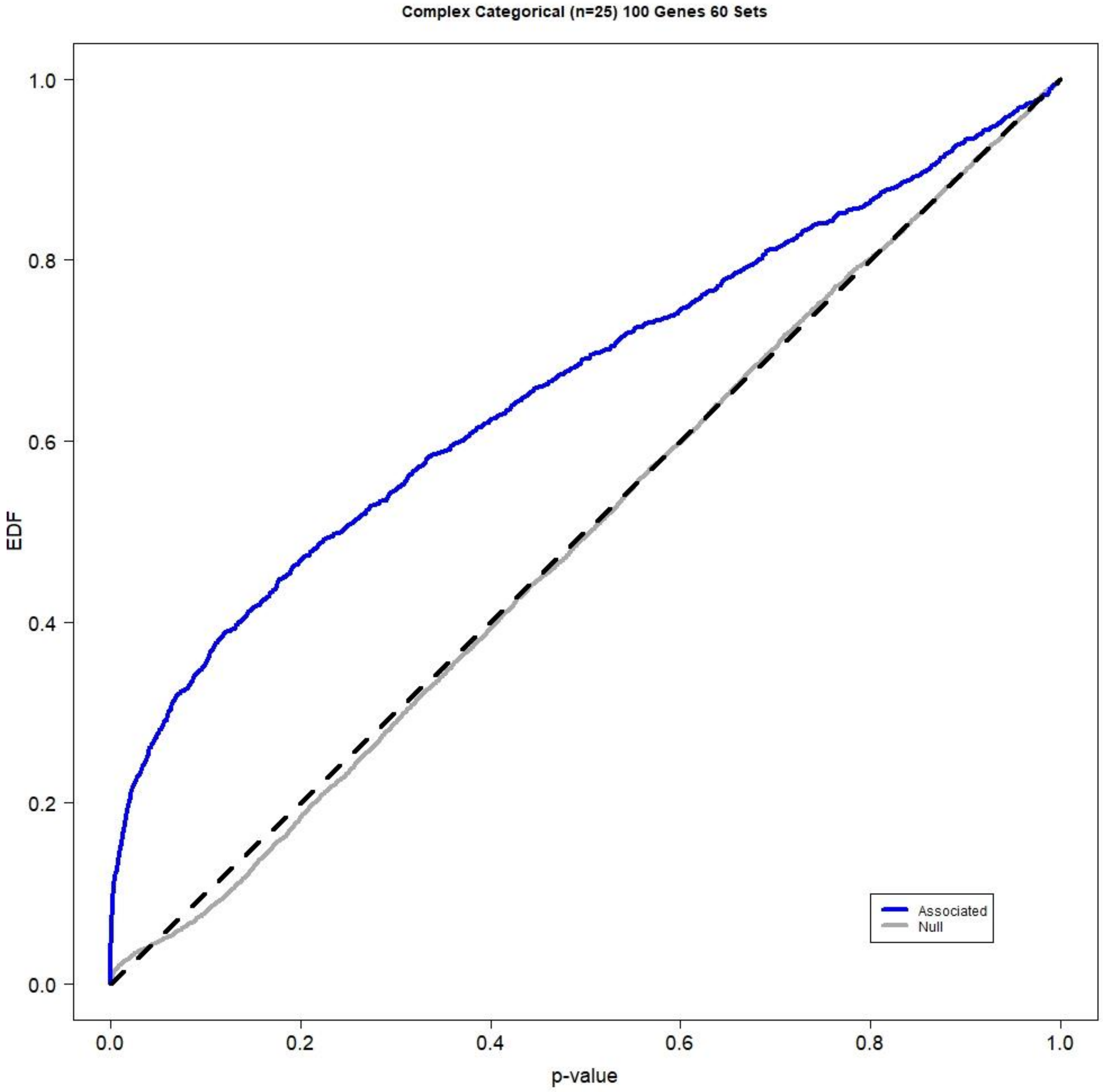
Complex Categorical (n=25) 100 Genes 60 Sets
EDF
p-value
0.0
0.2
0.4
0.6
0.8
1.0
0.0
0.2
0.4
0.6
0.8
1.0
Associated
Null

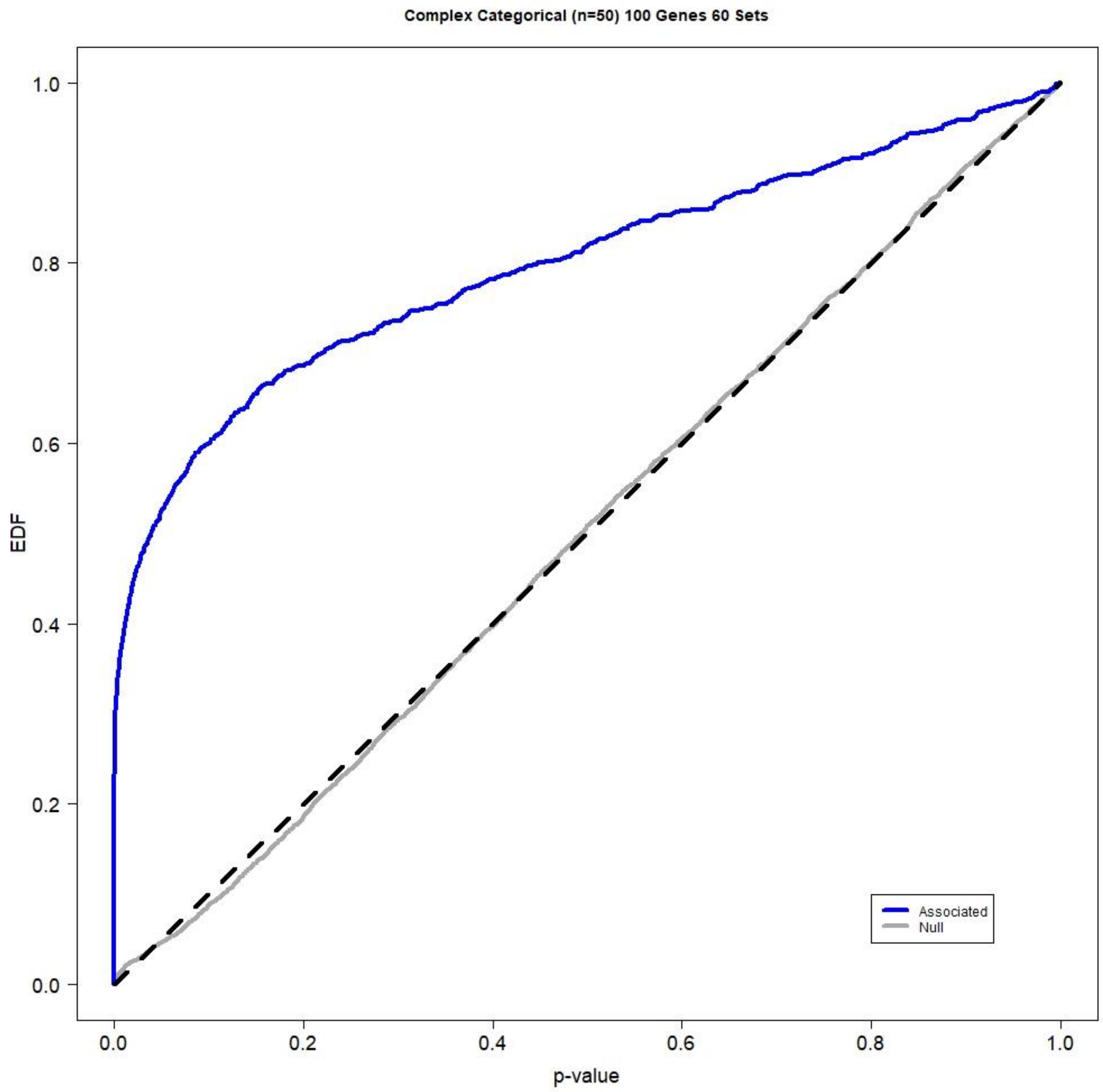

Complex Categorical (n=50) 100 Genes 60 Sets
EDF
p-value
0.0
0.2
0.4
0.6
0.8
1.0
Associated
Null

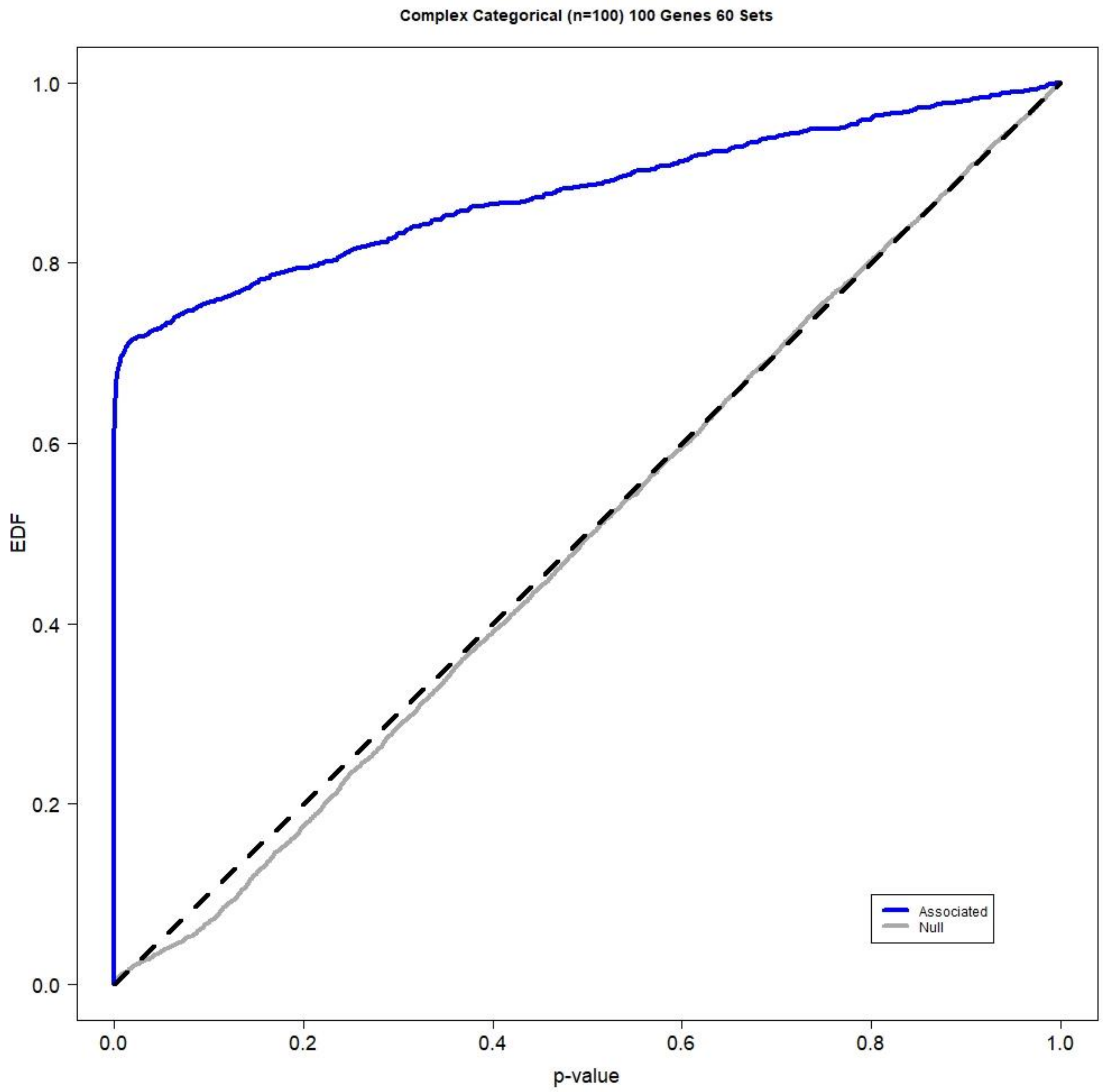
Complex Categorical (n=100) 100 Genes 60 Sets
EDF
p-value
0.0
0.2
0.4
0.6
0.8
1.0
Associated
Null

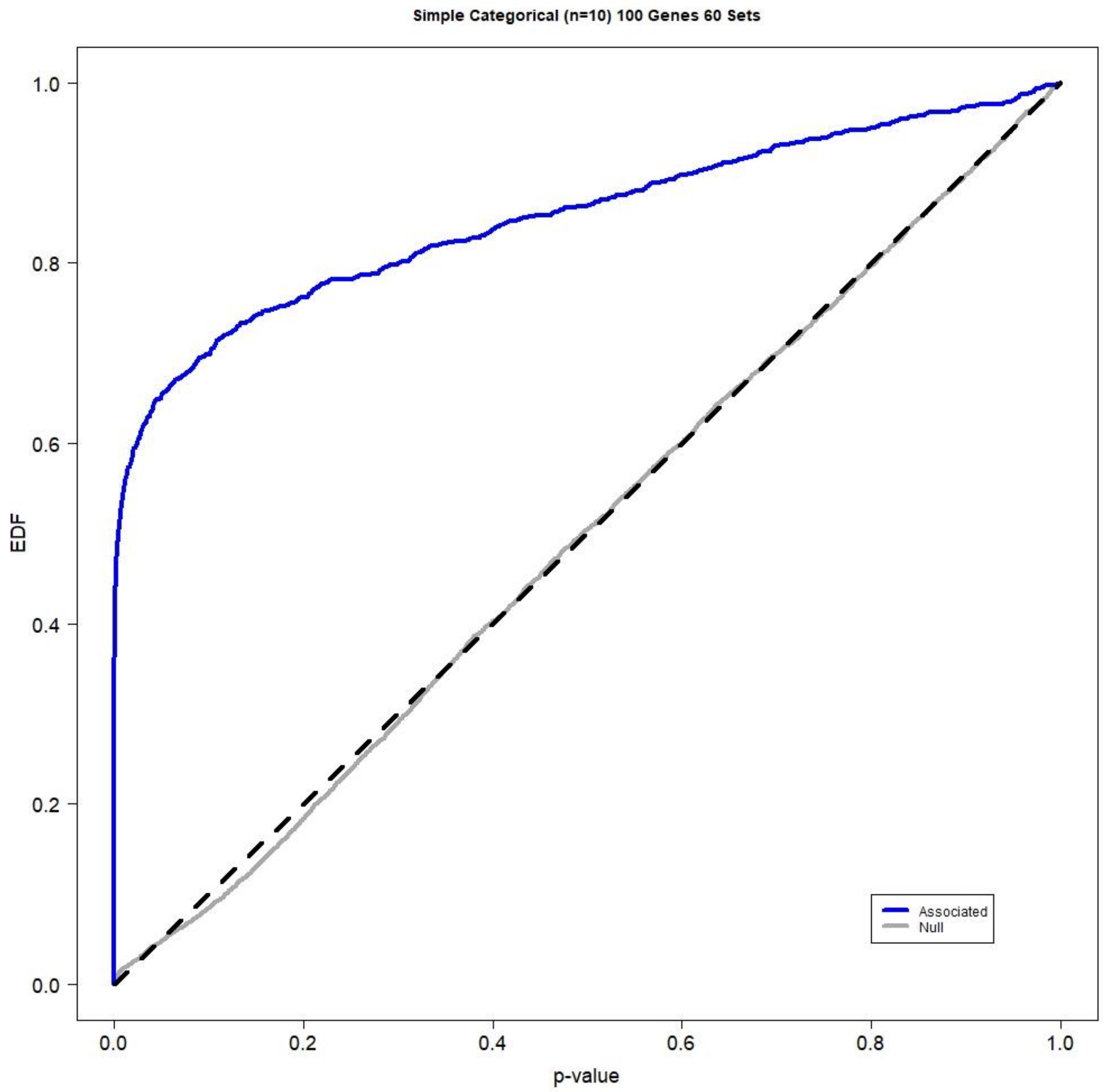

Simple Categorical (n=10) 100 Genes 60 Sets
EDF
p-value
0.0
0.2
0.4
0.6
0.8
1.0
Associated
Null

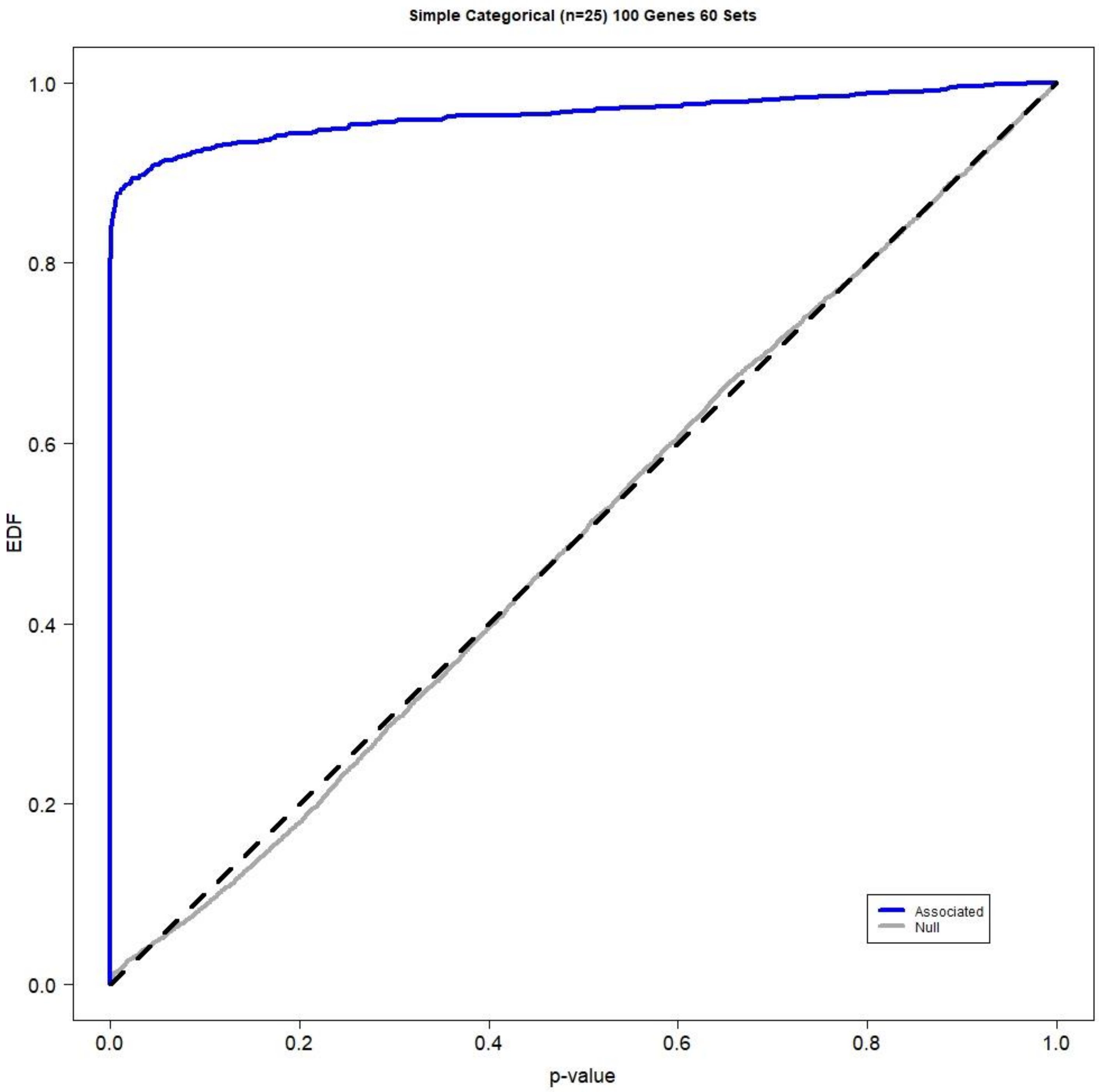
Simple Categorical (n=25) 100 Genes 60 Sets
EDF
p-value
0.0
0.2
0.4
0.6
0.8
1.0
Associated
Null

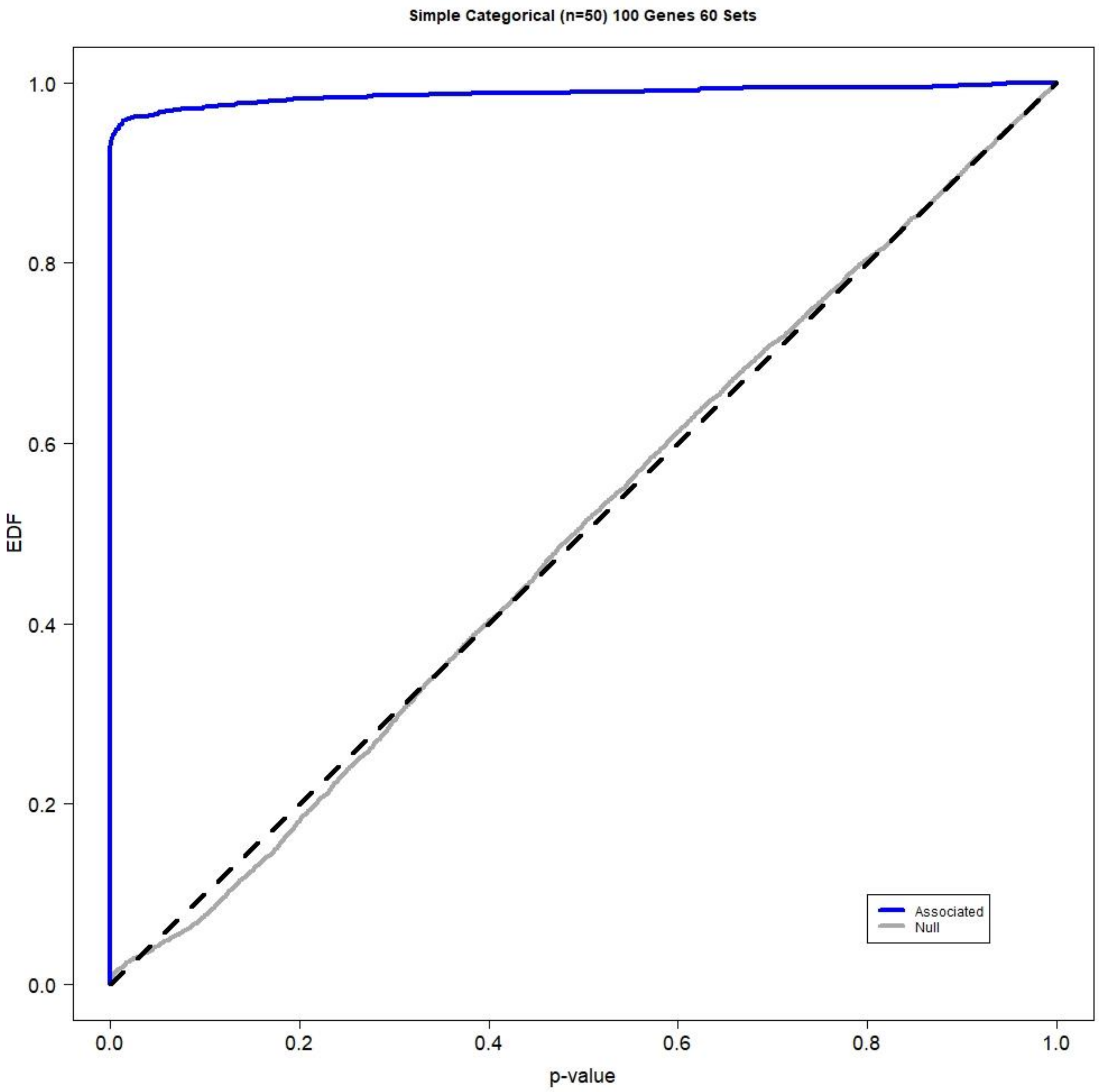
Simple Categorical (n=50) 100 Genes 60 Sets
EDF
p-value
0.0
0.2
0.4
0.6
0.8
1.0
Associated
Null

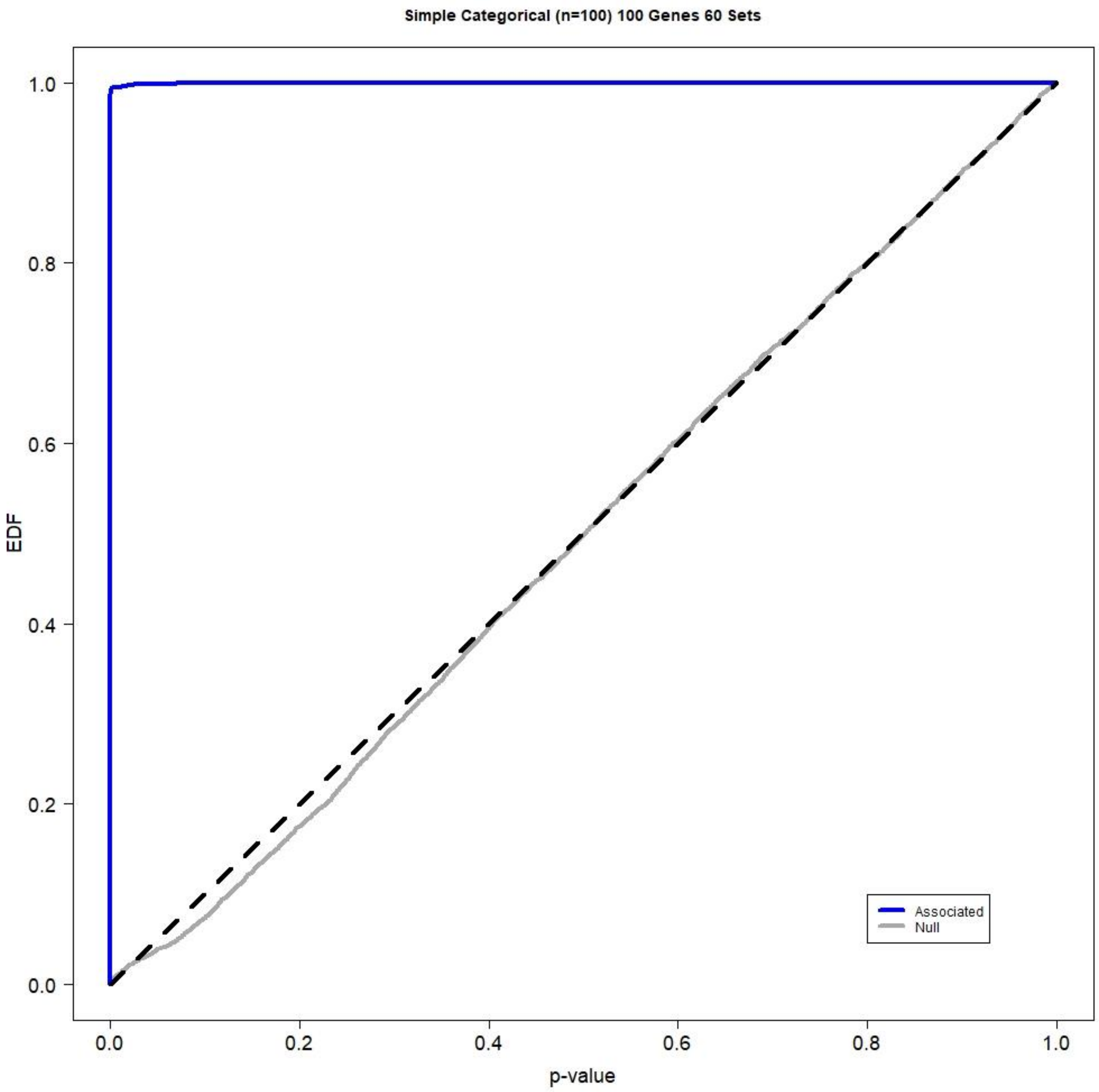

Simple Categorical (n=100) 100 Genes 60 Sets
EDF
p-value
Associated
Null
0.0
0.2
0.4
0.6
0.8
1.0

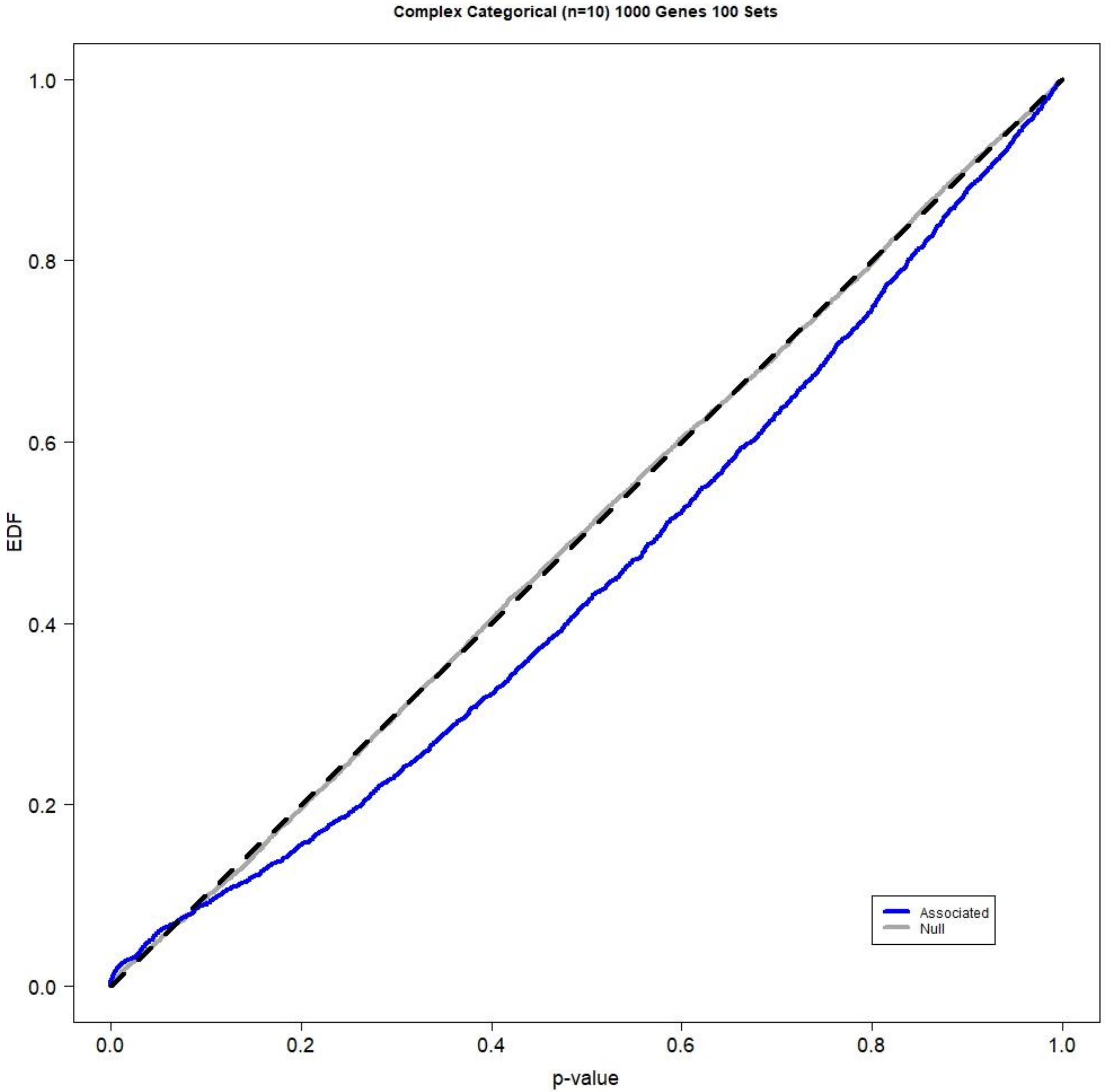

Complex Categorical (n=10) 1000 Genes 100 Sets
EDF
p-value
0.0
0.2
0.4
0.6
0.8
1.0
Associated
Null

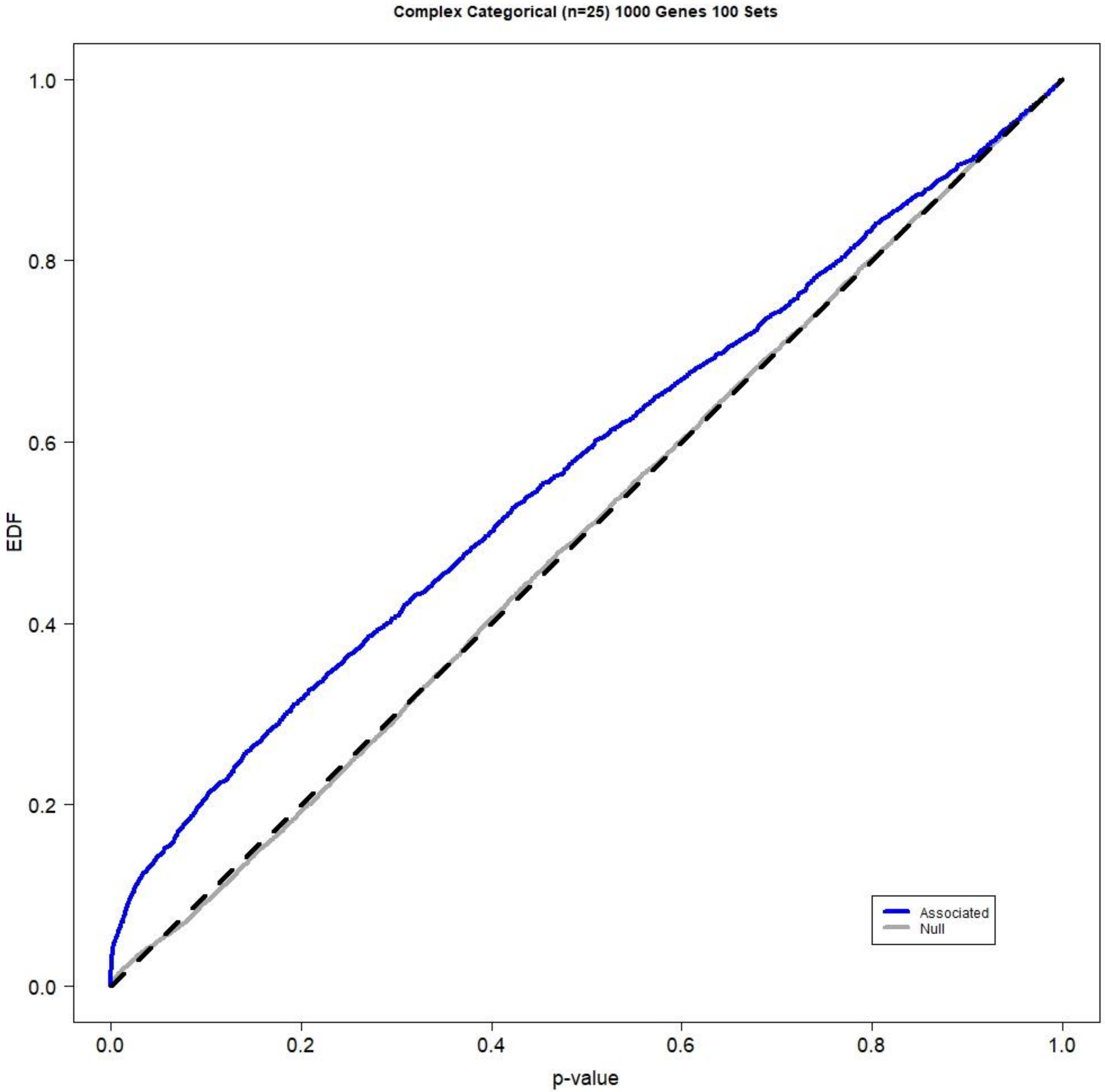

Complex Categorical (n=25) 1000 Genes 100 Sets
EDF
p-value
0.0
0.2
0.4
0.6
0.8
1.0
Associated
Null

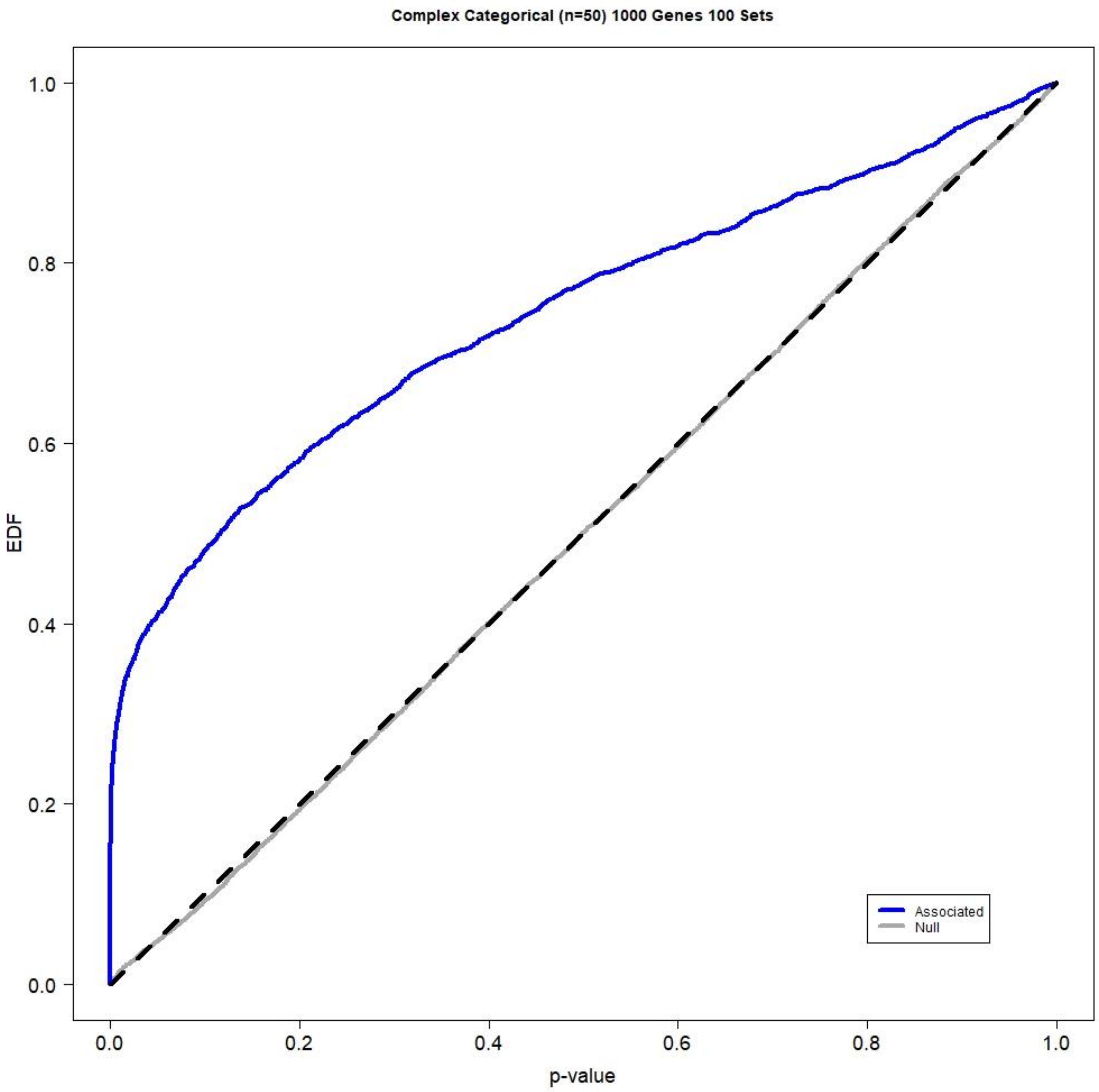

Complex Categorical (n=50) 1000 Genes 100 Sets
EDF
p-value
0.0
0.2
0.4
0.6
0.8
1.0
Associated
Null

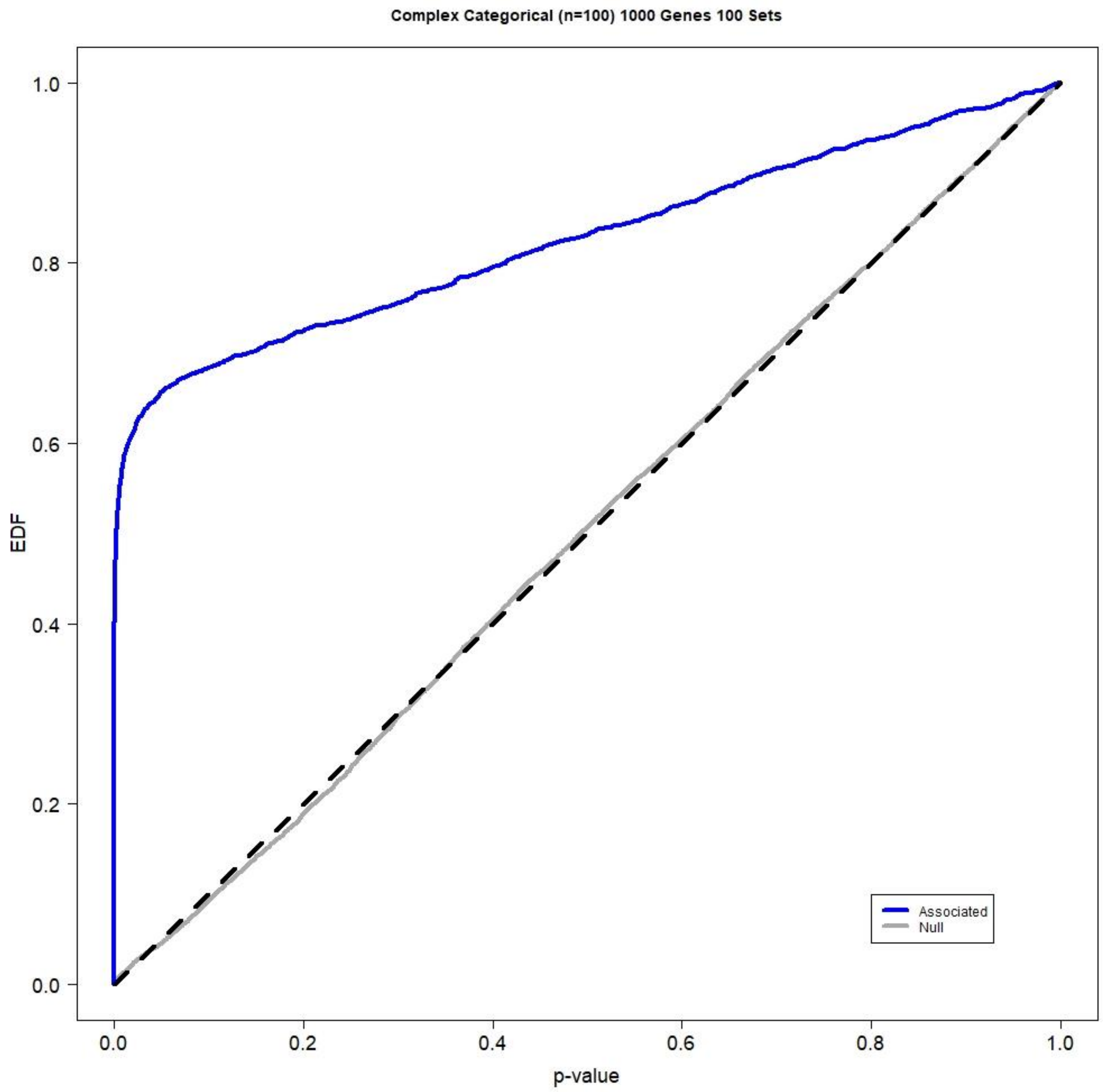
Complex Categorical (n=100) 1000 Genes 100 Sets
EDF
p-value
0.0
0.2
0.4
0.6
0.8
1.0
Associated
Null

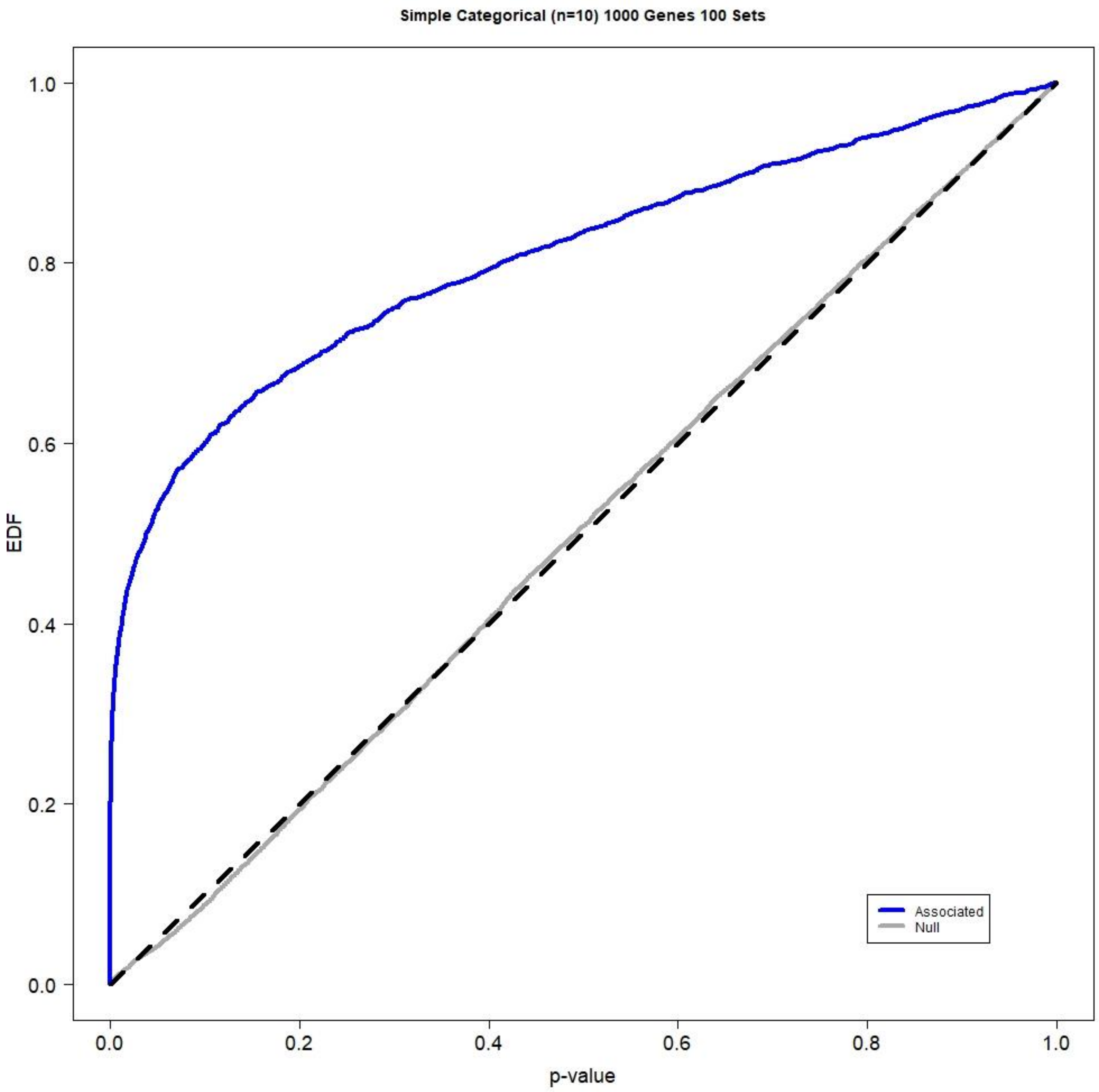

Simple Categorical (n=10) 1000 Genes 100 Sets
EDF
p-value
0.0
0.2
0.4
0.6
0.8
1.0
Associated
Null

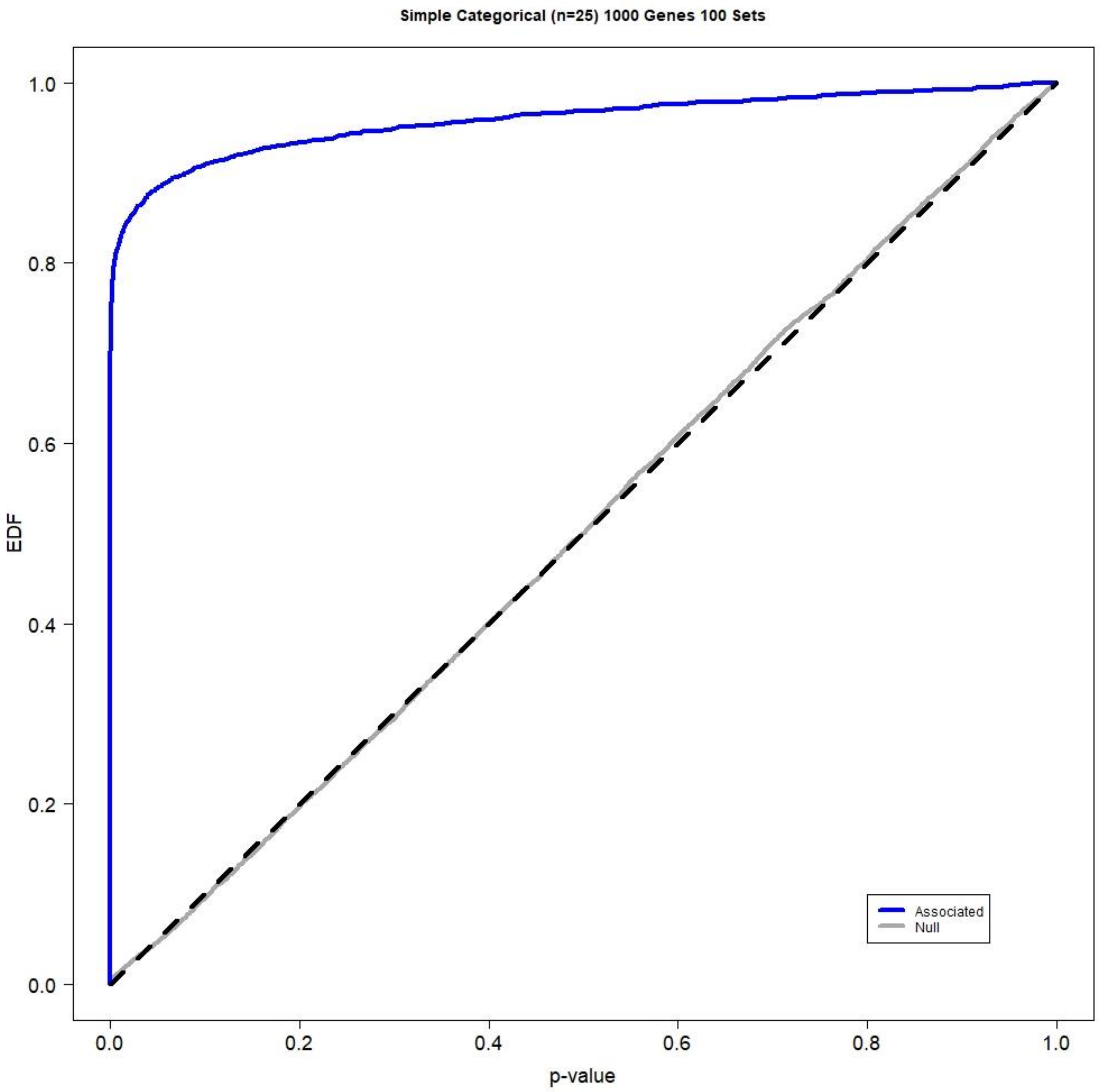

Simple Categorical (n=25) 1000 Genes 100 Sets
EDF
p-value
0.0
0.2
0.4
0.6
0.8
1.0
Associated
Null

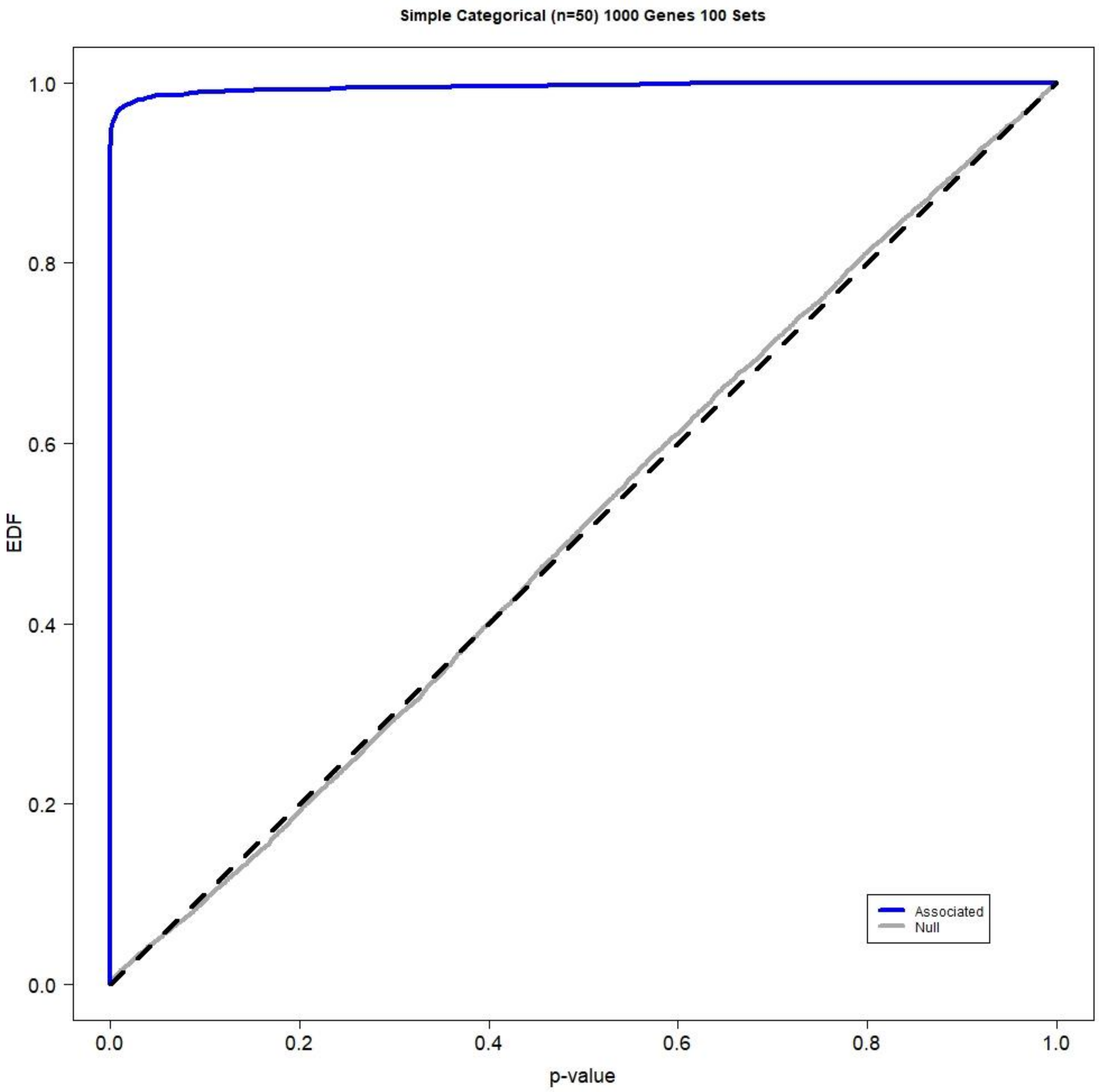
Simple Categorical (n=50) 1000 Genes 100 Sets
1.0
0.8
0.6
0.4
0.2
0.0
EDF
0.0
0.2
0.4
0.6
0.8
1.0
p-value
Associated
Null

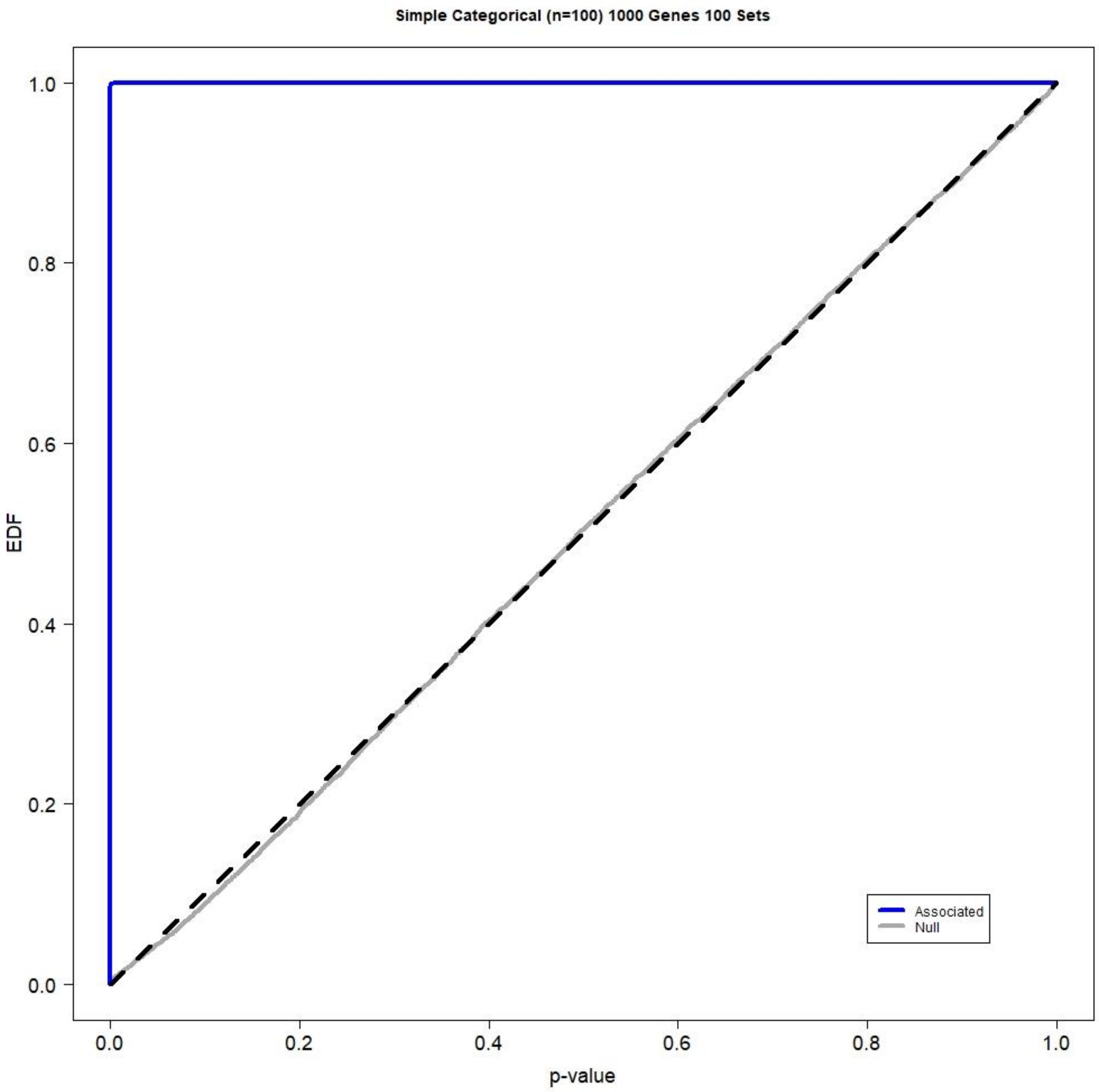

Simple Categorical (n=100) 1000 Genes 100 Sets
EDF
p-value
0.0
0.2
0.4
0.6
0.8
1.0
Associated
Null

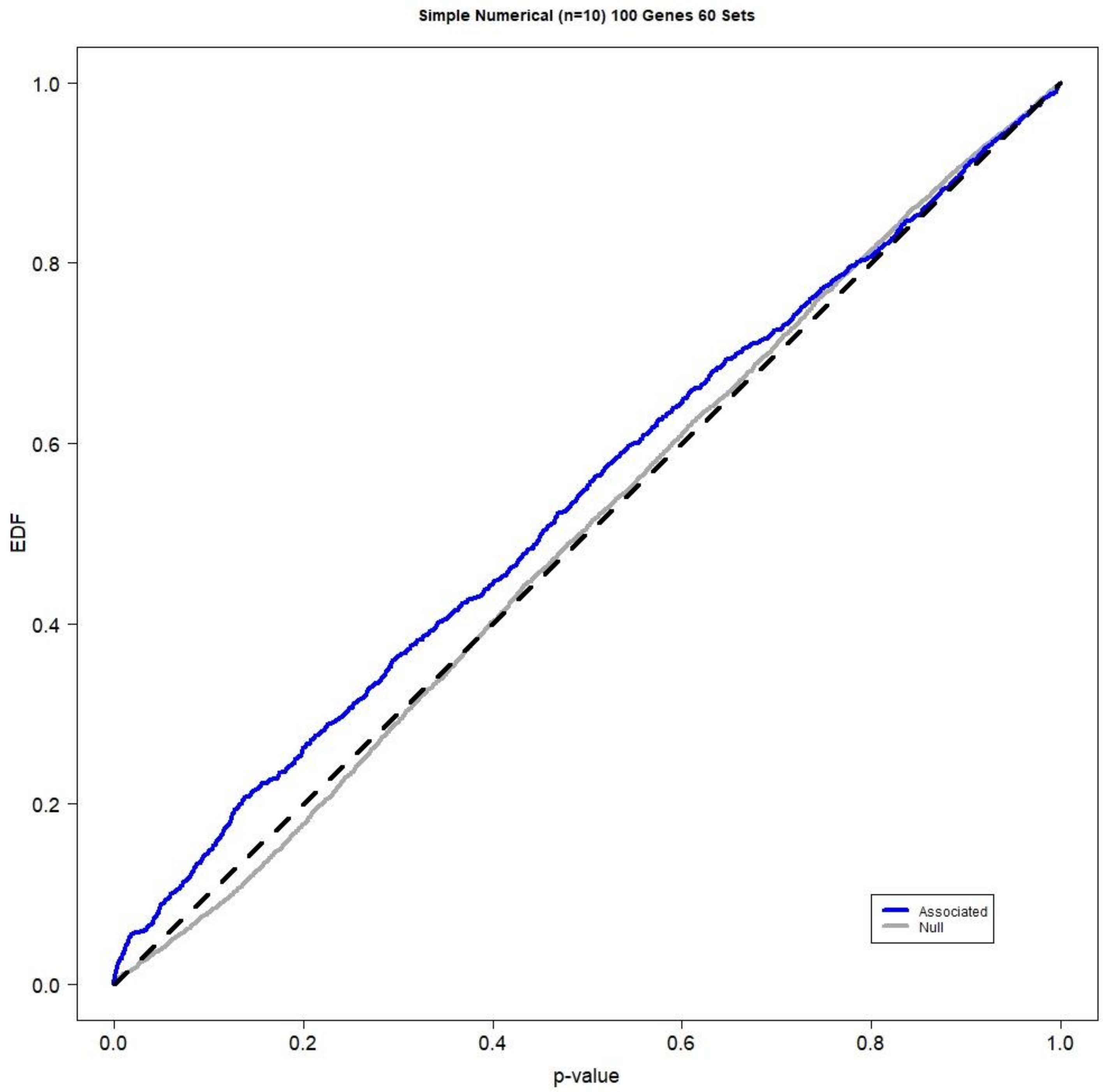
Simple Numerical (n=10) 100 Genes 60 Sets
EDF
p-value
0.0
0.2
0.4
0.6
0.8
1.0
Associated
Null

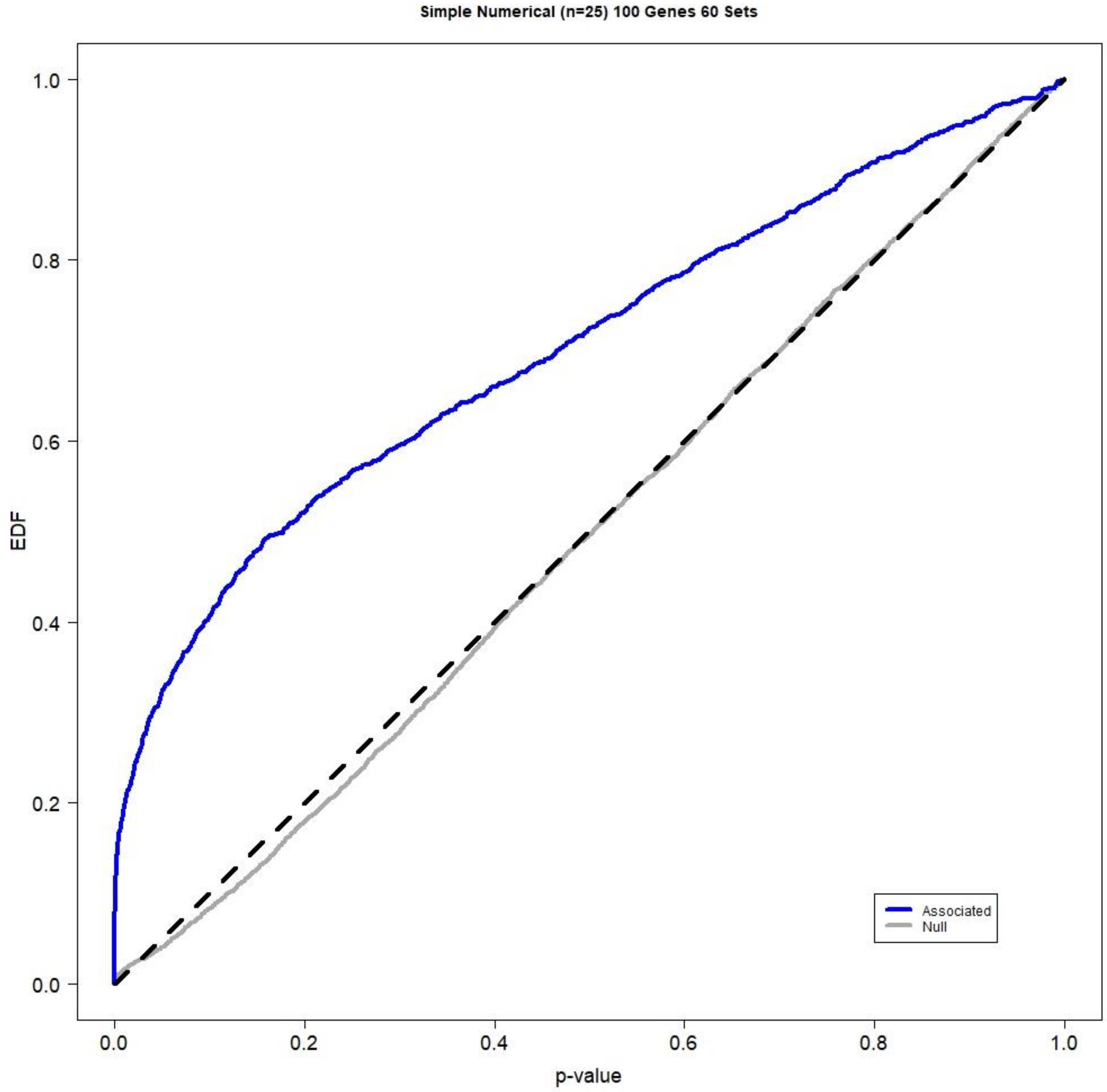
Simple Numerical (n=25) 100 Genes 60 Sets
EDF
p-value
0.0
0.2
0.4
0.6
0.8
1.0
Associated
Null

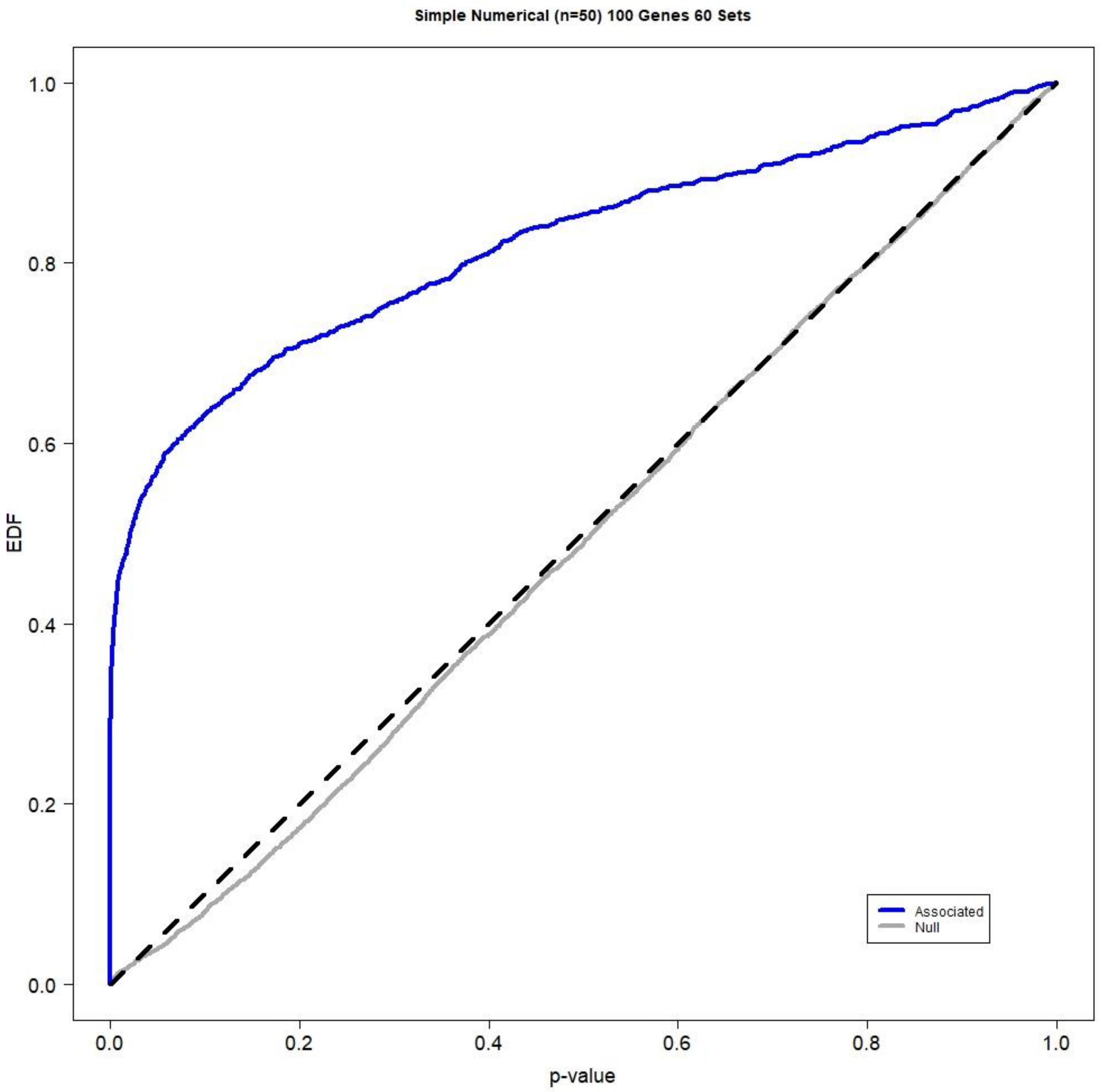

Simple Numerical (n=50) 100 Genes 60 Sets
EDF
p-value
0.0
0.2
0.4
0.6
0.8
1.0
Associated
Null

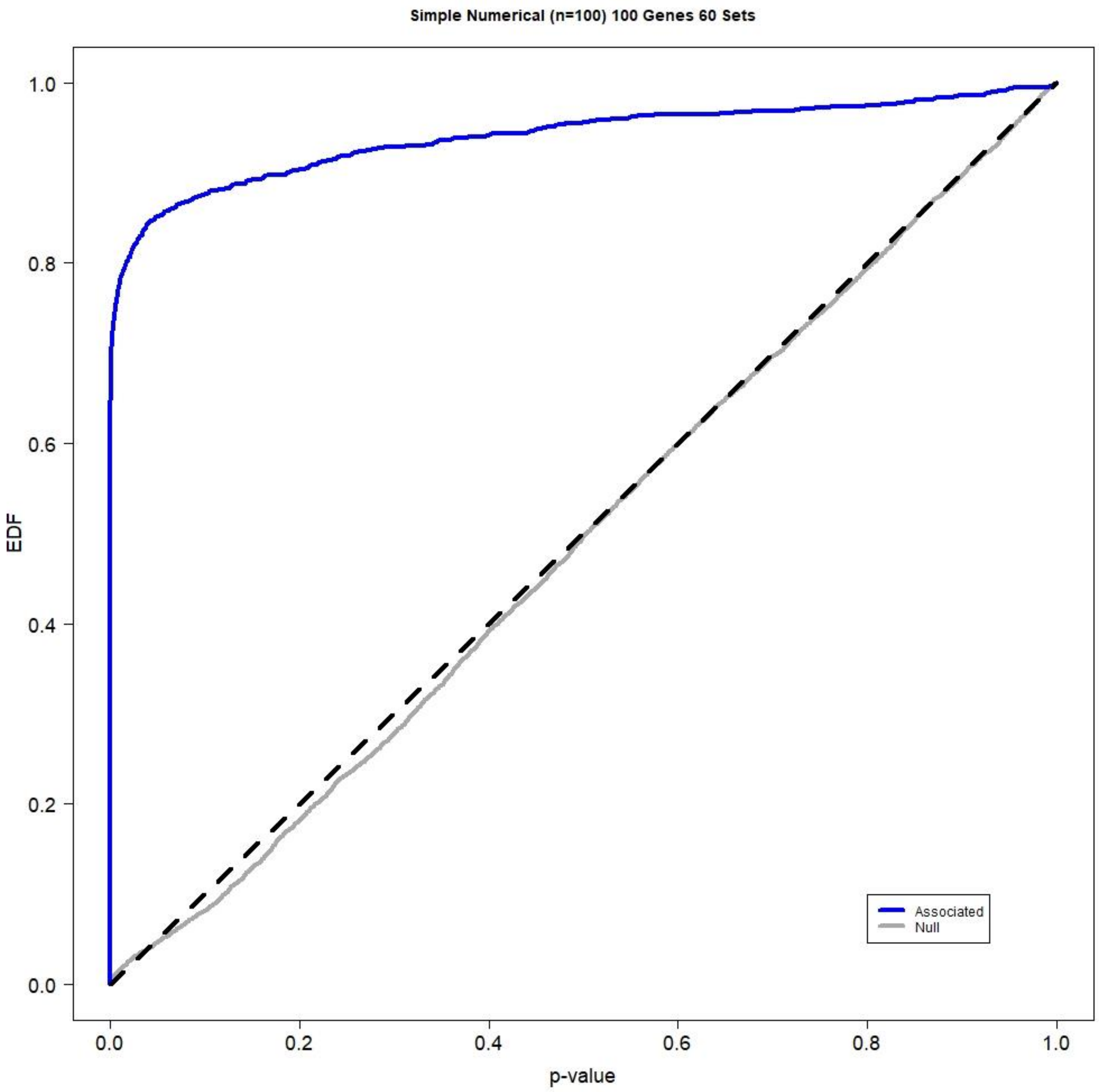

Simple Numerical (n=100) 100 Genes 60 Sets
EDF
p-value
0.0
0.2
0.4
0.6
0.8
1.0
Associated
Null

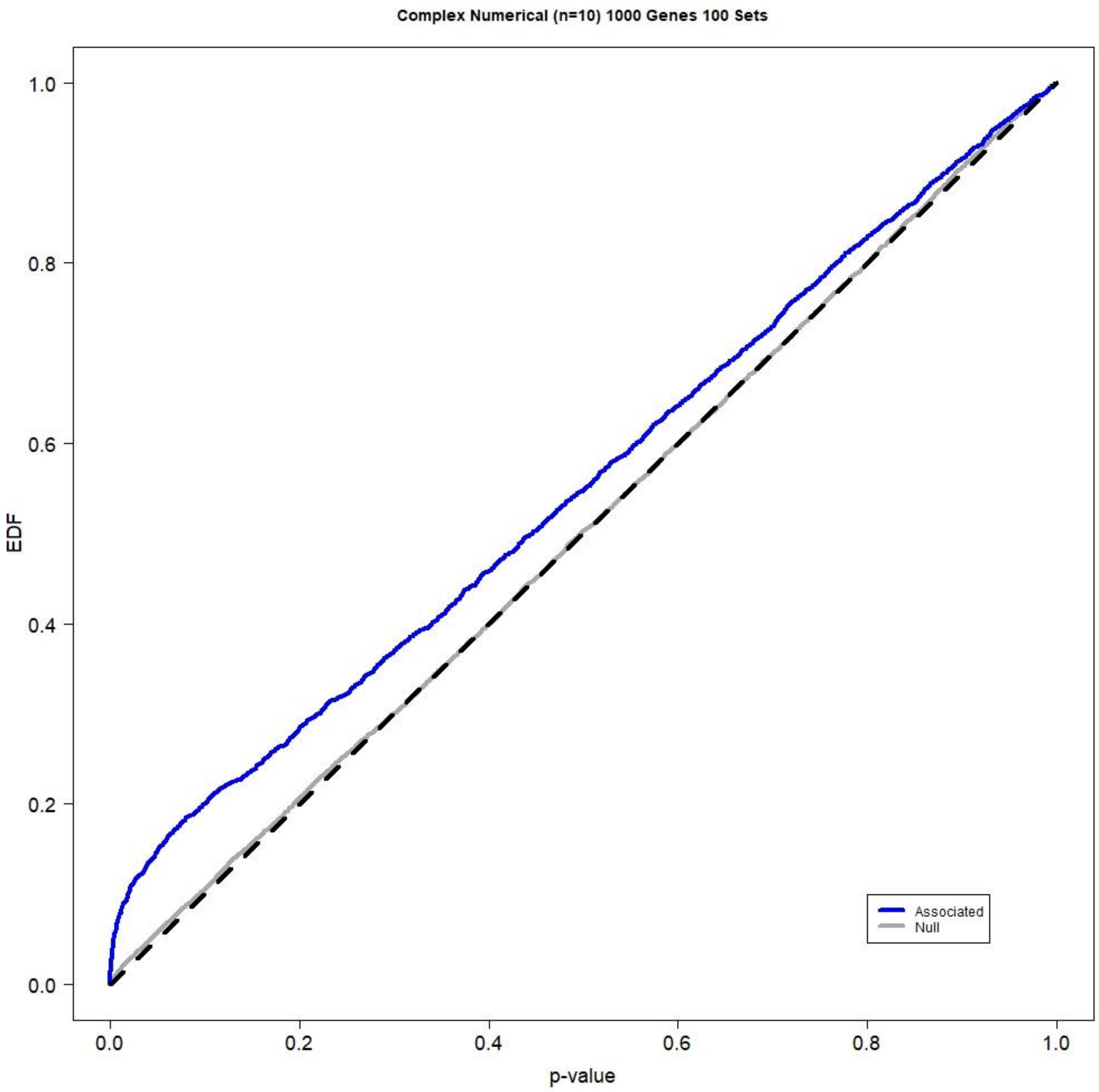
Complex Numerical (n=10) 1000 Genes 100 Sets
EDF
p-value
0.0
0.2
0.4
0.6
0.8
1.0
Associated
Null

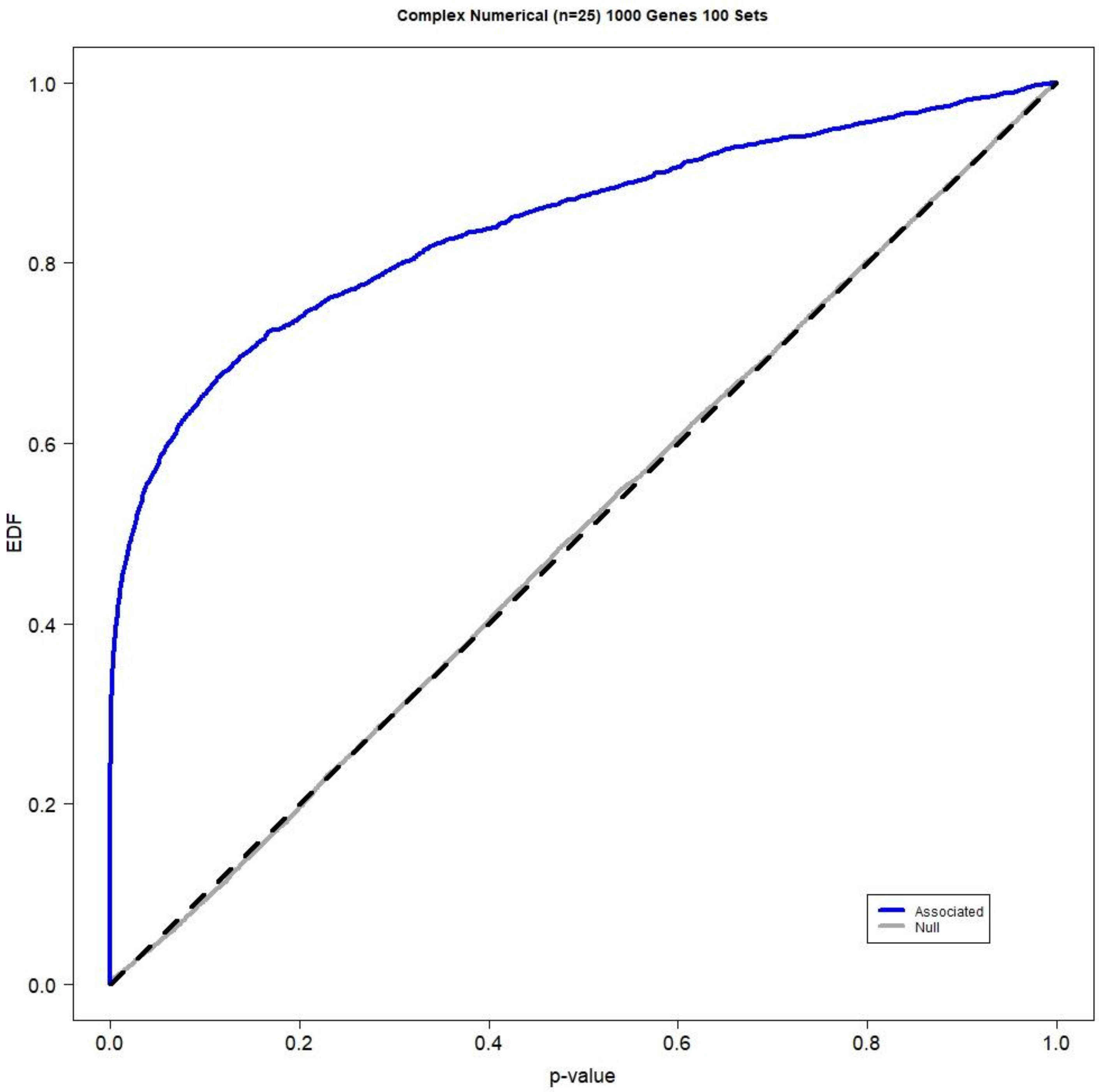

Complex Numerical (n=25) 1000 Genes 100 Sets
EDF
p-value
0.0
0.2
0.4
0.6
0.8
1.0
Associated
Null

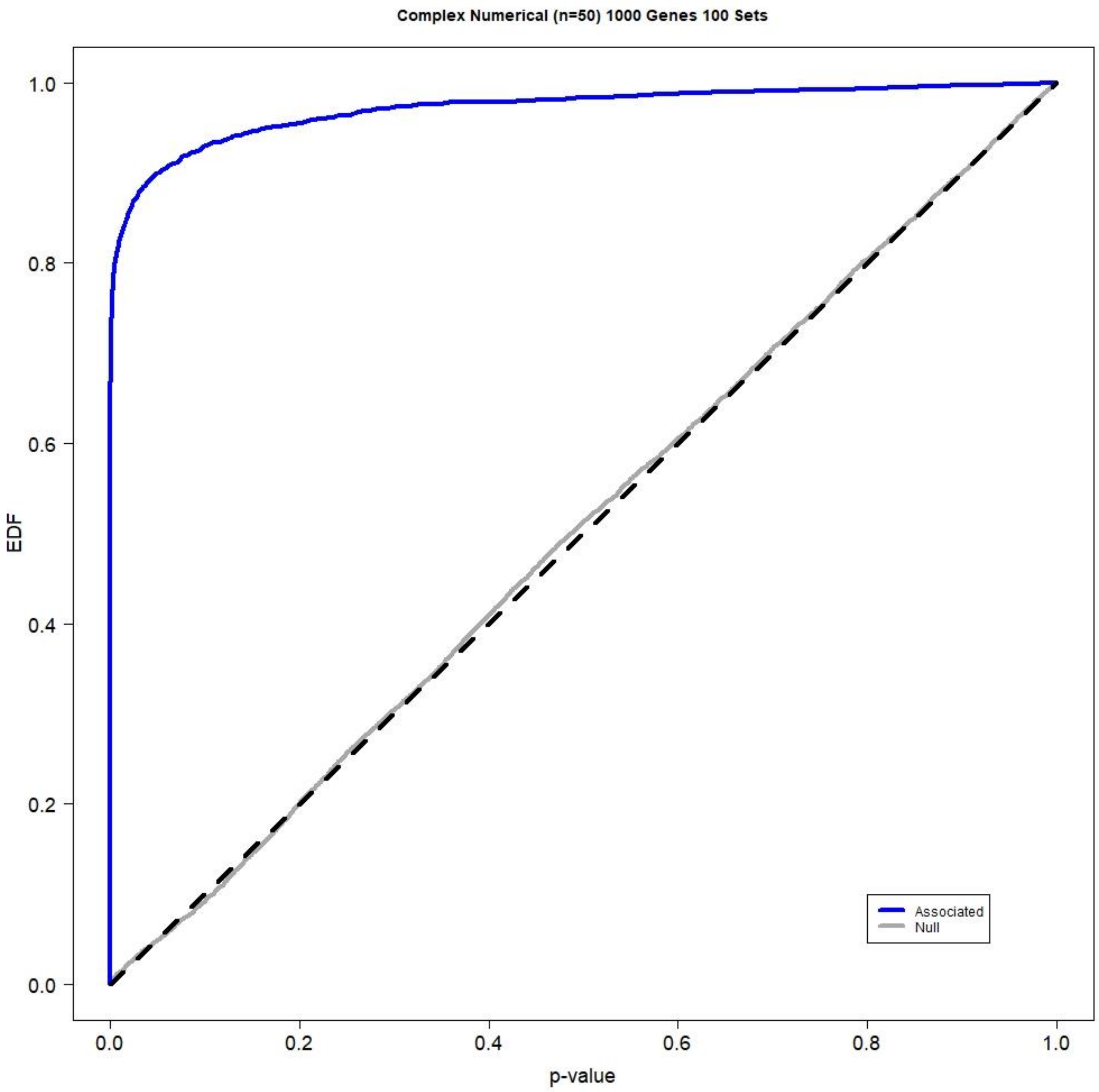
Complex Numerical (n=50) 1000 Genes 100 Sets
EDF
p-value
0.0
0.2
0.4
0.6
0.8
1.0
Associated
Null

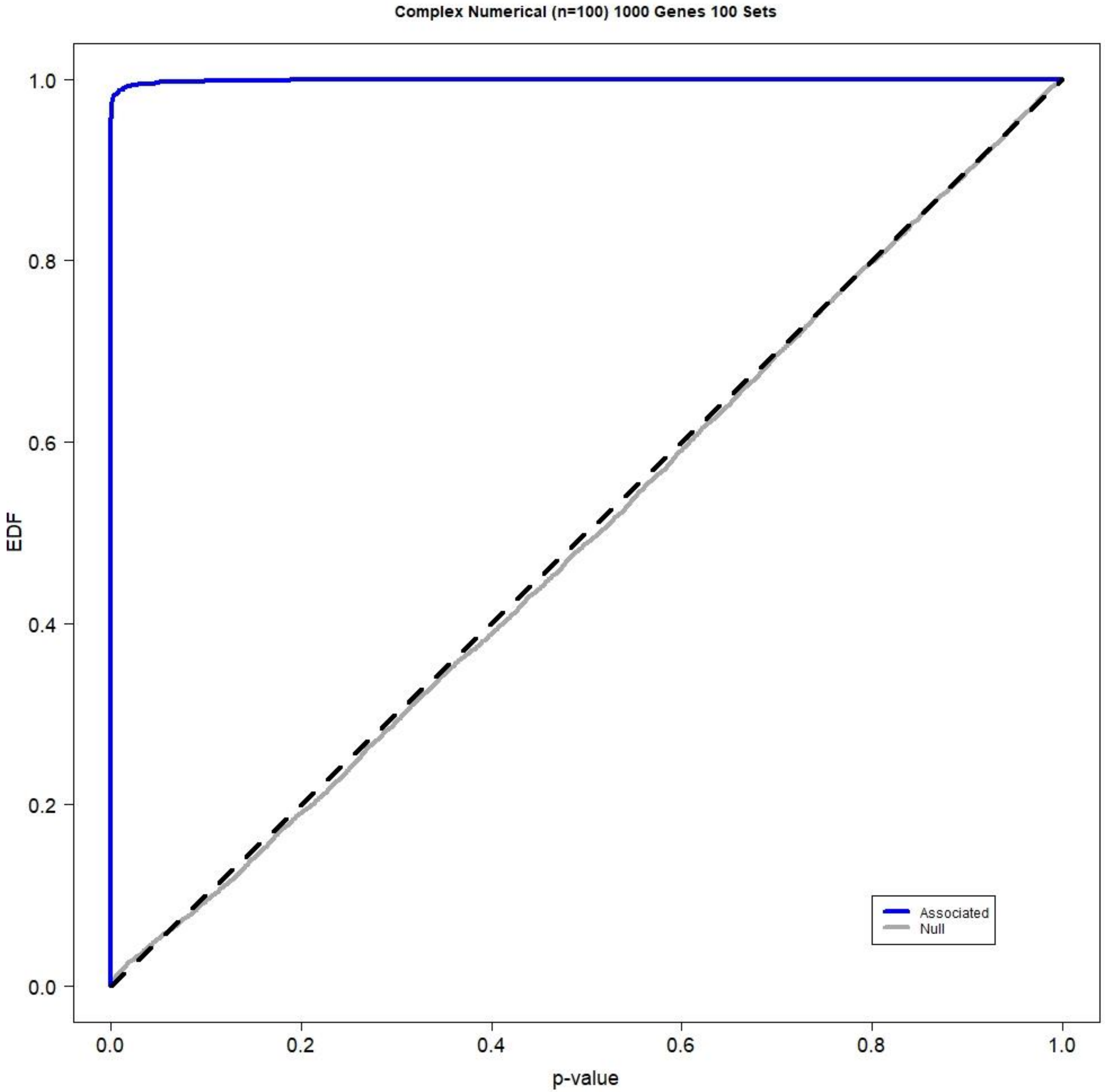
Complex Numerical (n=100) 1000 Genes 100 Sets
EDF
p-value
0.0
0.2
0.4
0.6
0.8
1.0
Associated
Null

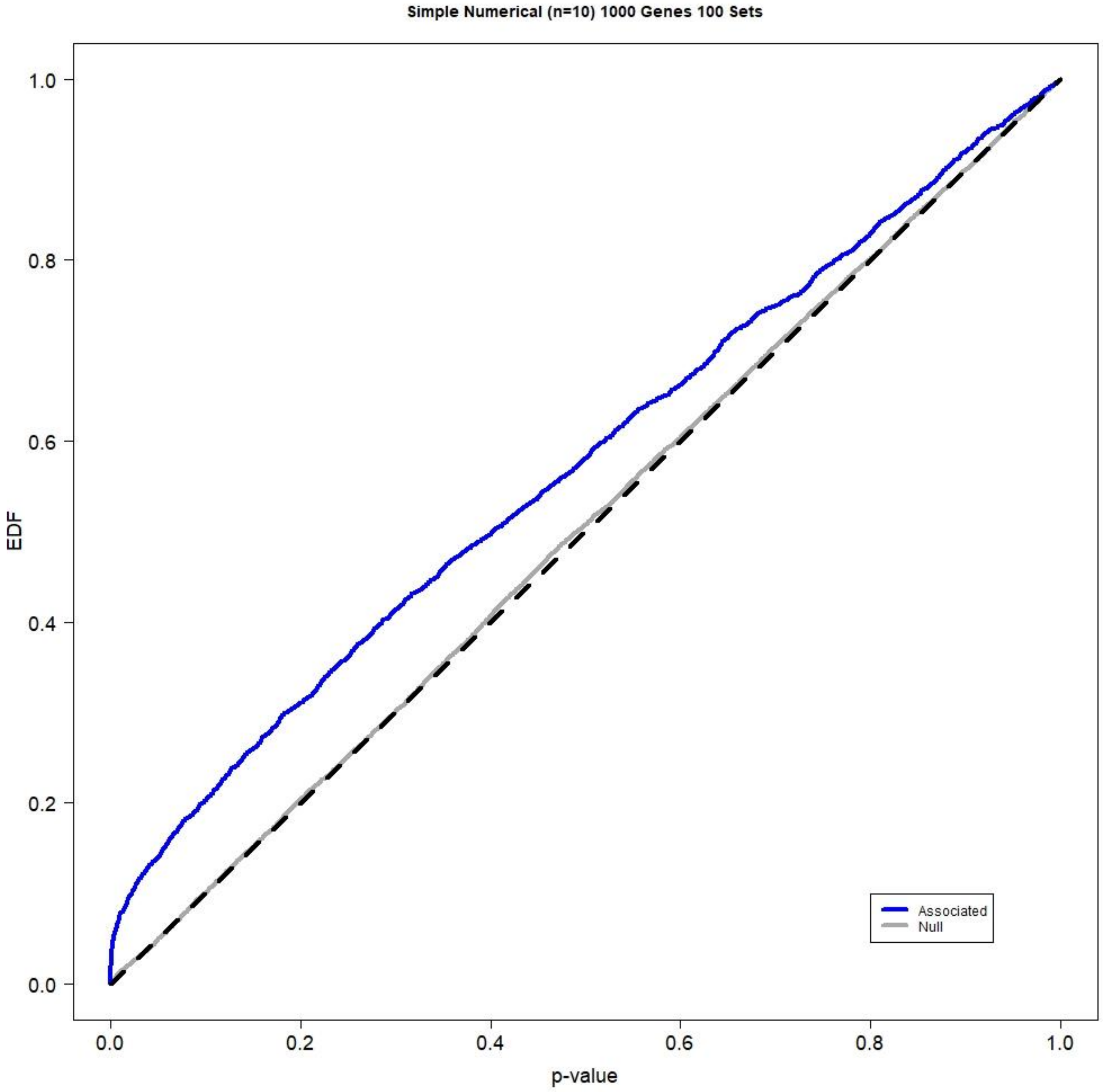
Simple Numerical (n=10) 1000 Genes 100 Sets
EDF
p-value
0.0
0.2
0.4
0.6
0.8
1.0
Associated
Null

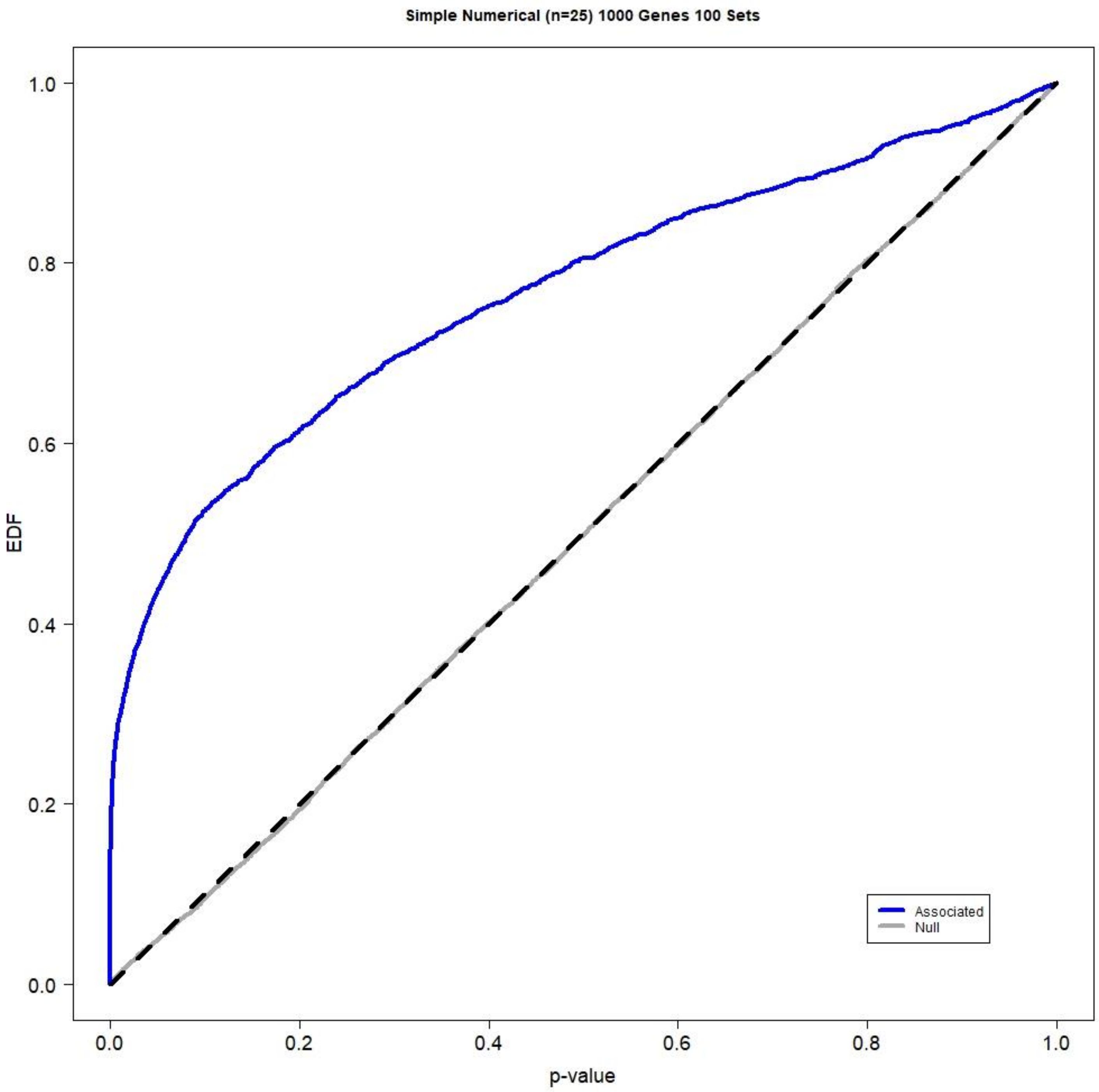
Simple Numerical (n=25) 1000 Genes 100 Sets
1.0
0.8
0.6
0.4
0.2
0.0
EDF
0.0
0.2
0.4
0.6
0.8
1.0
p-value
Associated
Null

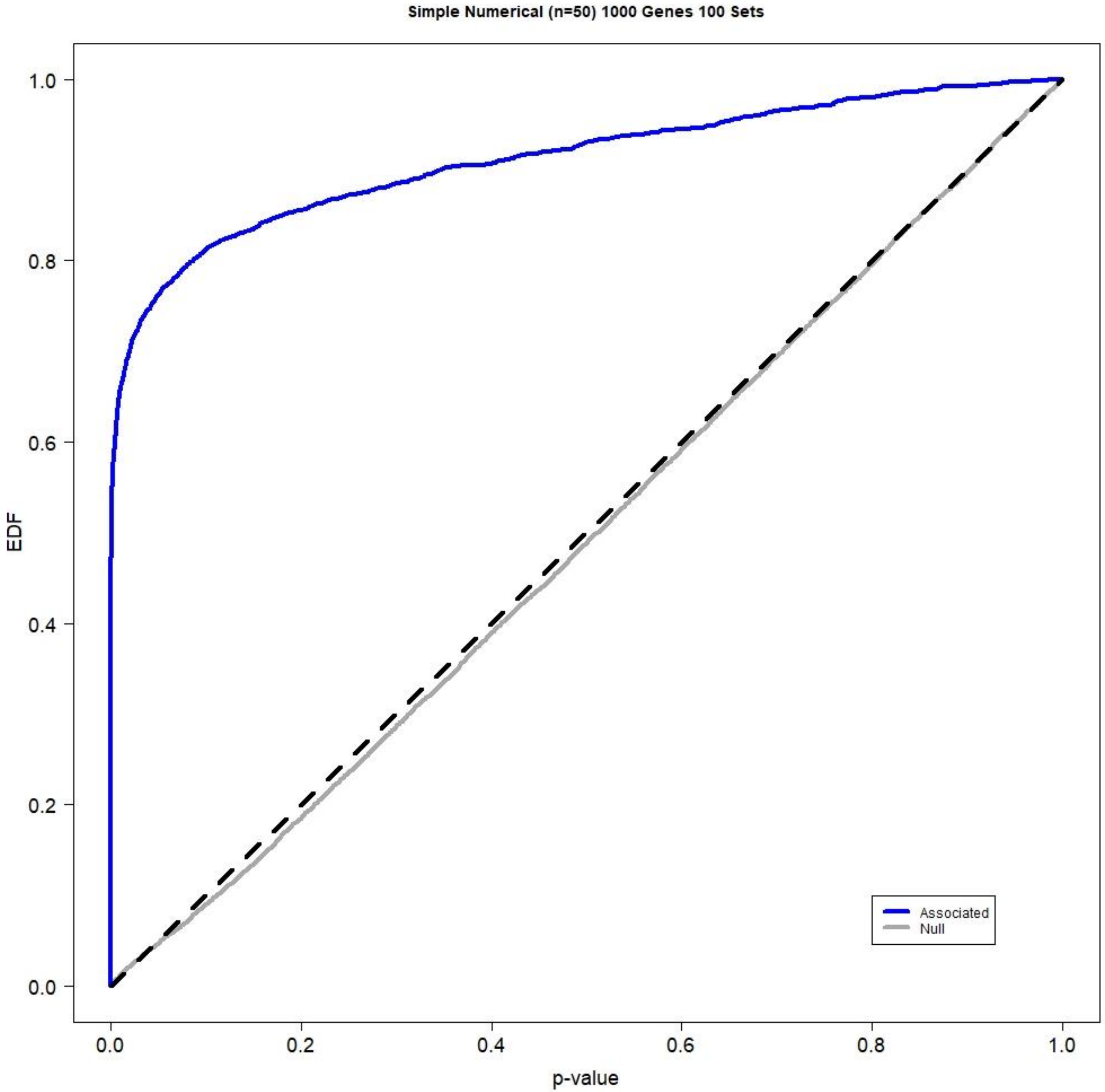
Simple Numerical (n=50) 1000 Genes 100 Sets
EDF
p-value
0.0
0.2
0.4
0.6
0.8
1.0
0.0
0.2
0.4
0.6
0.8
1.0
Associated
Null

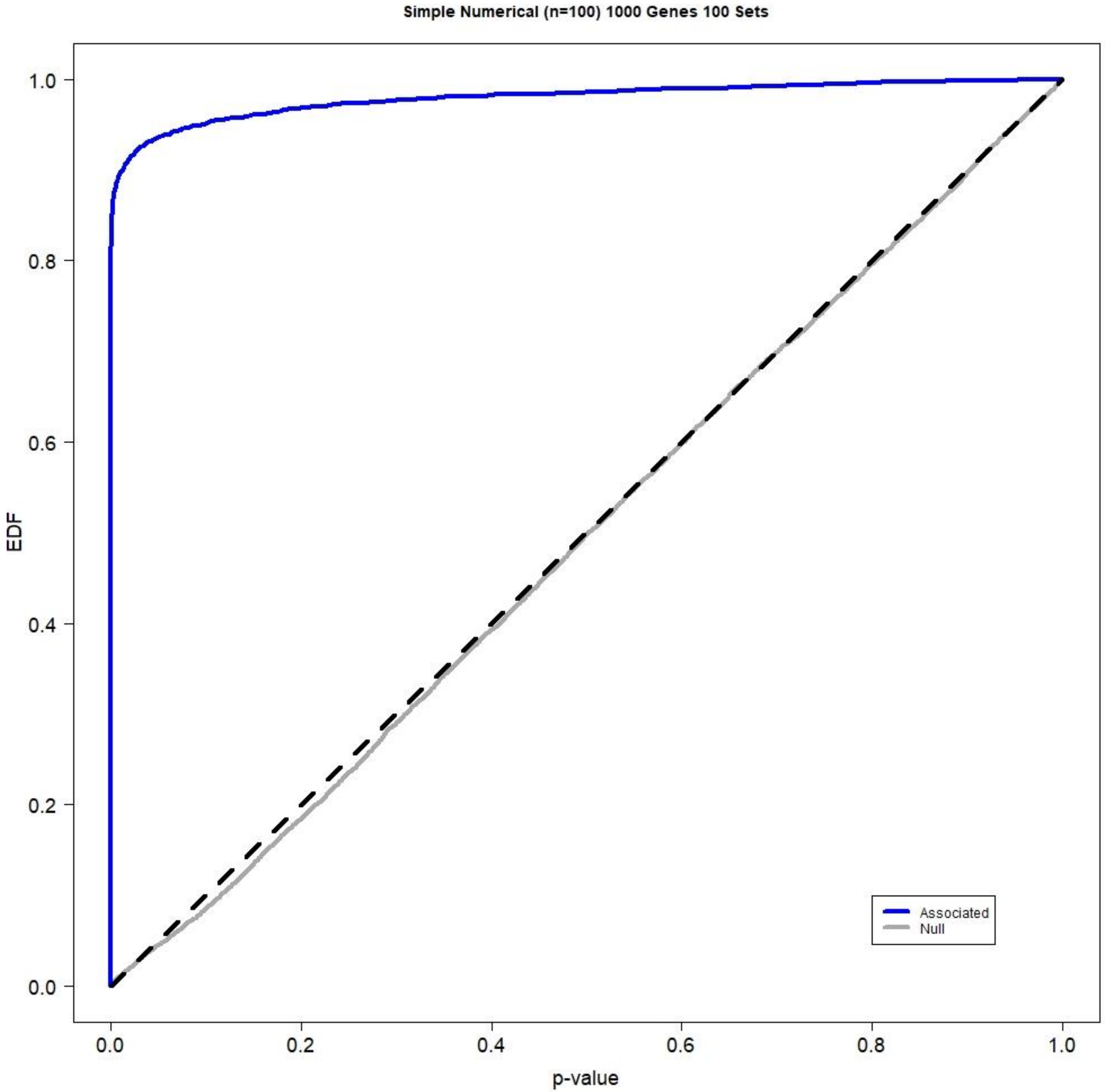

Simple Numerical (n=100) 1000 Genes 100 Sets
EDF
p-value
0.0
0.2
0.4
0.6
0.8
1.0
Associated
Null

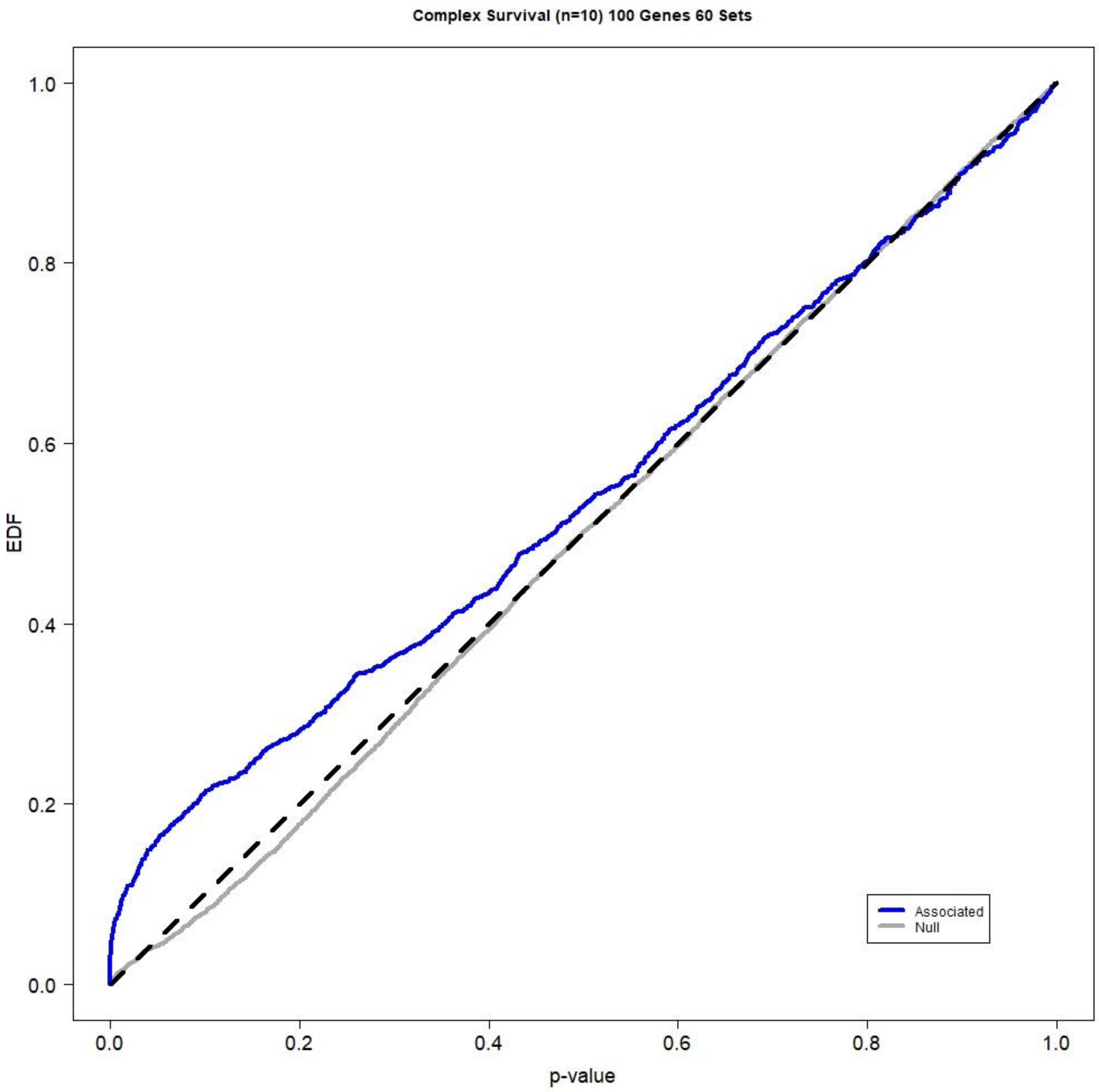
Complex Survival (n=10) 100 Genes 60 Sets
EDF
p-value
0.0
0.2
0.4
0.6
0.8
1.0
Associated
Null

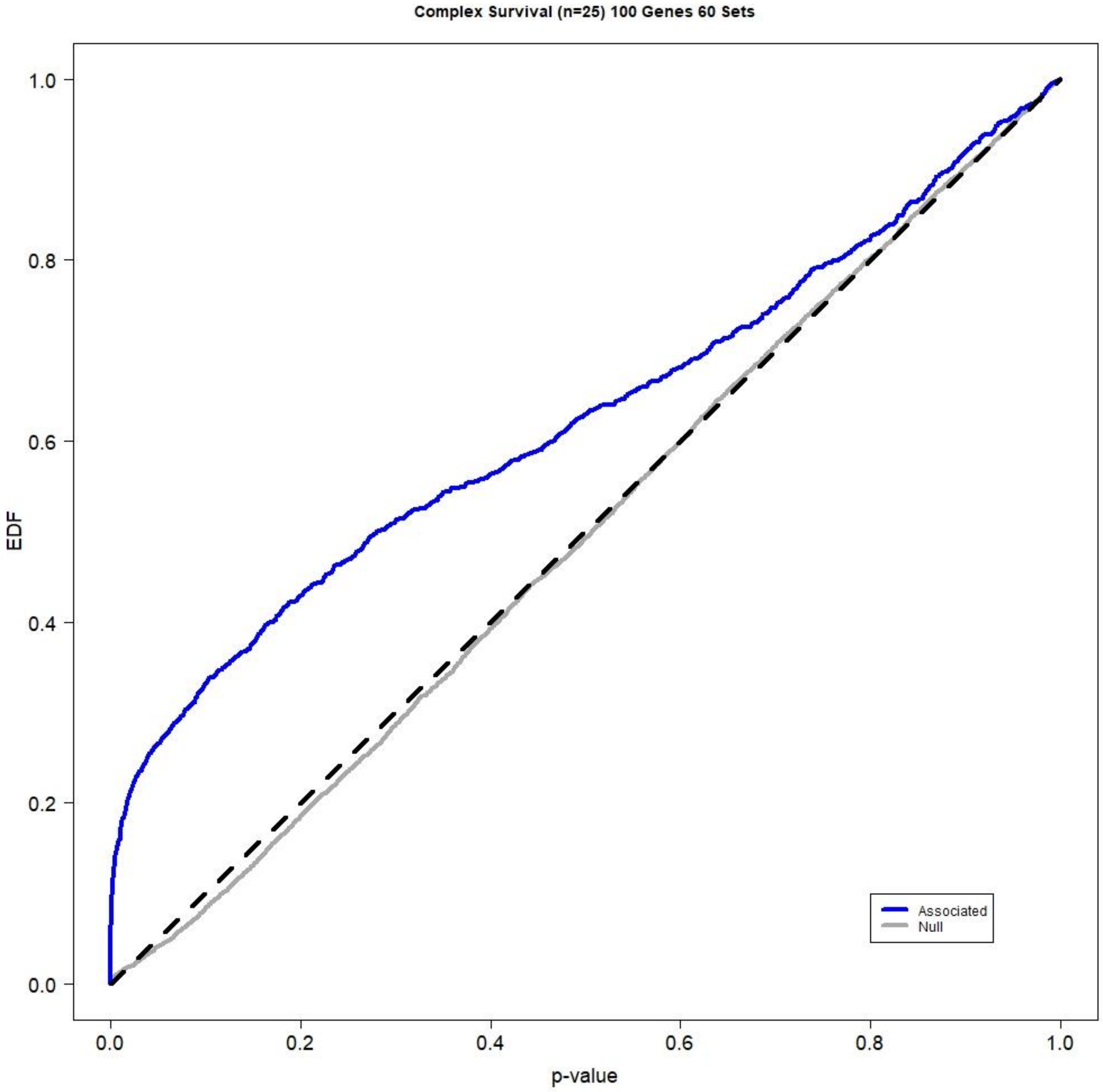
Complex Survival (n=25) 100 Genes 60 Sets
EDF
p-value
0.0
0.2
0.4
0.6
0.8
1.0
0.0
0.2
0.4
0.6
0.8
1.0
Associated
Null

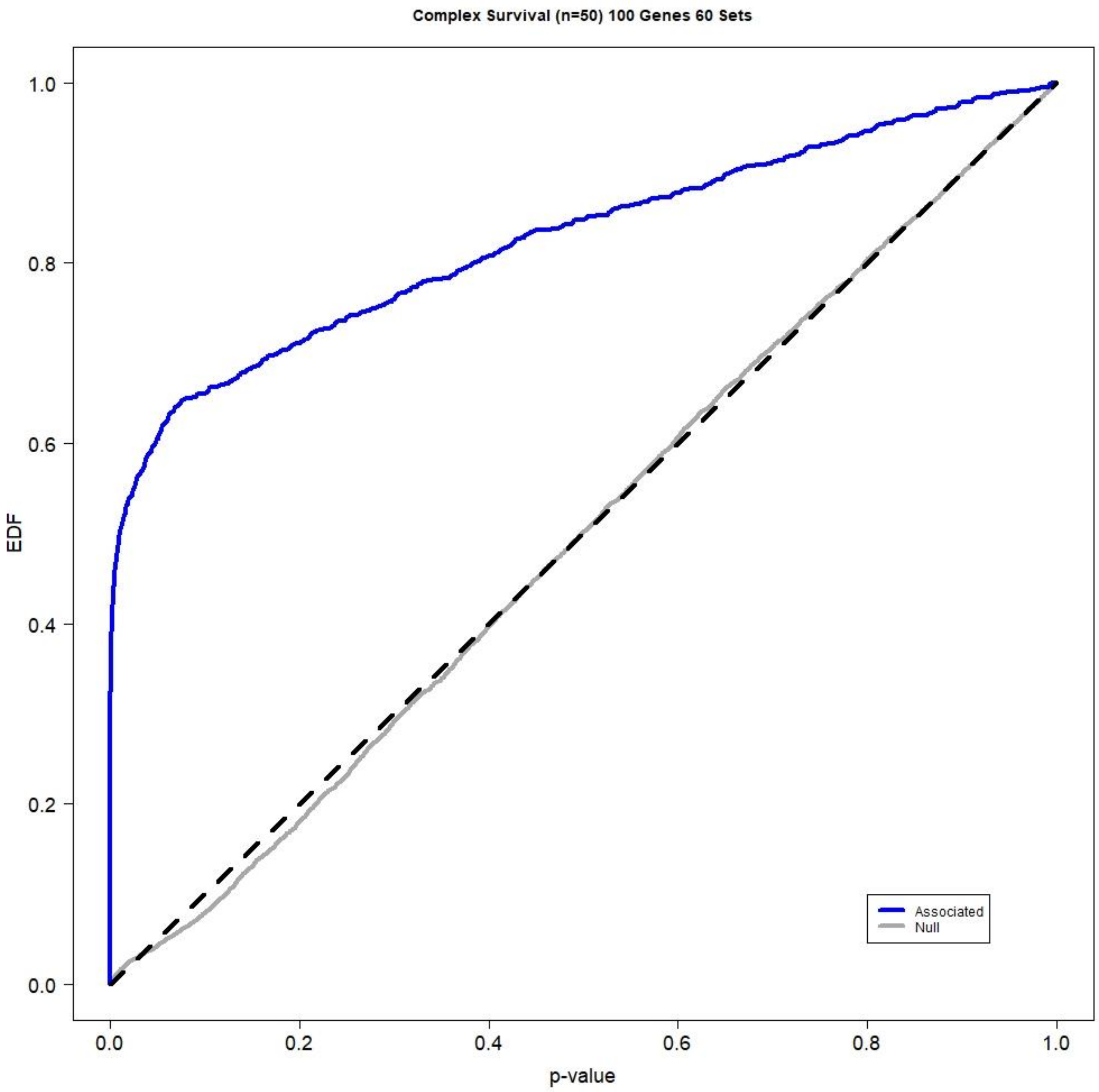

Complex Survival (n=50) 100 Genes 60 Sets
EDF
p-value
0.0
0.2
0.4
0.6
0.8
1.0
0.0
0.2
0.4
0.6
0.8
1.0
Associated
Null

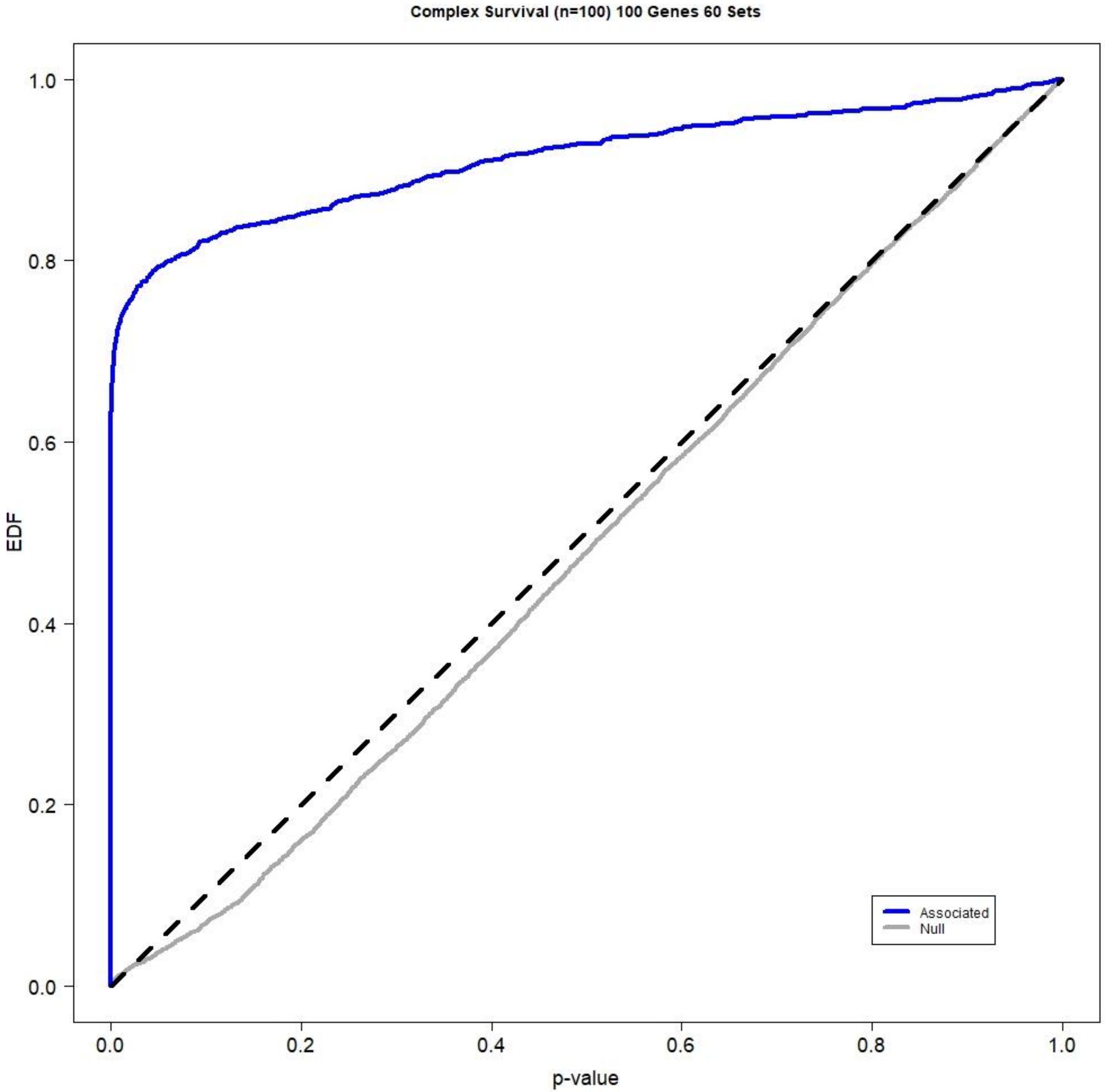

Complex Survival (n=100) 100 Genes 60 Sets
EDF
p-value
0.0
0.2
0.4
0.6
0.8
1.0
Associated
Null

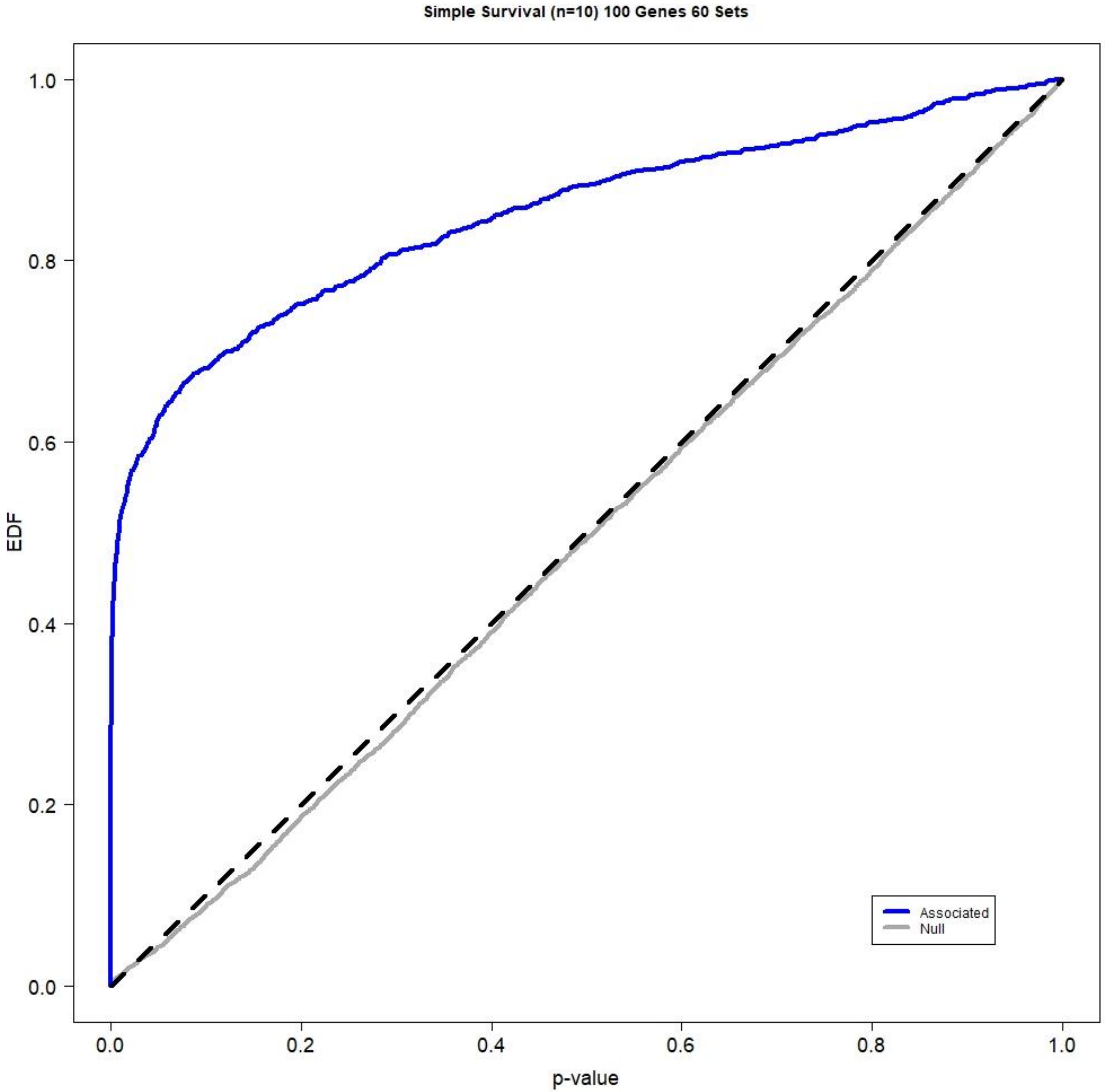
Simple Survival (n=10) 100 Genes 60 Sets
1.0
0.8
0.6
0.4
0.2
0.0
EDF
0.0
0.2
0.4
0.6
0.8
1.0
p-value
Associated
Null

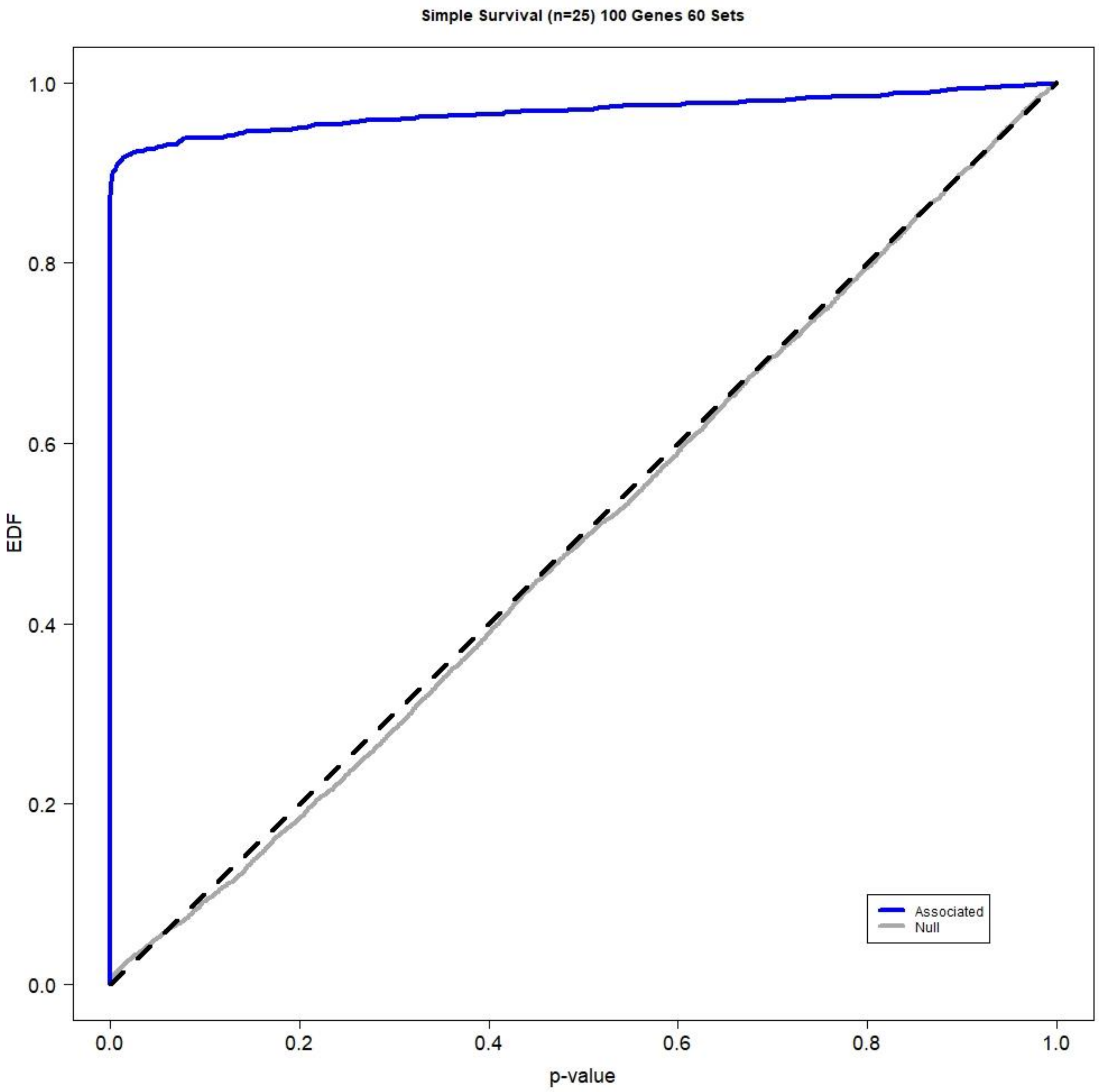

Simple Survival (n=25) 100 Genes 60 Sets
1.0
0.8
0.6
0.4
0.2
0.0
EDF
0.0
0.2
0.4
0.6
0.8
1.0
p-value
Associated
Null

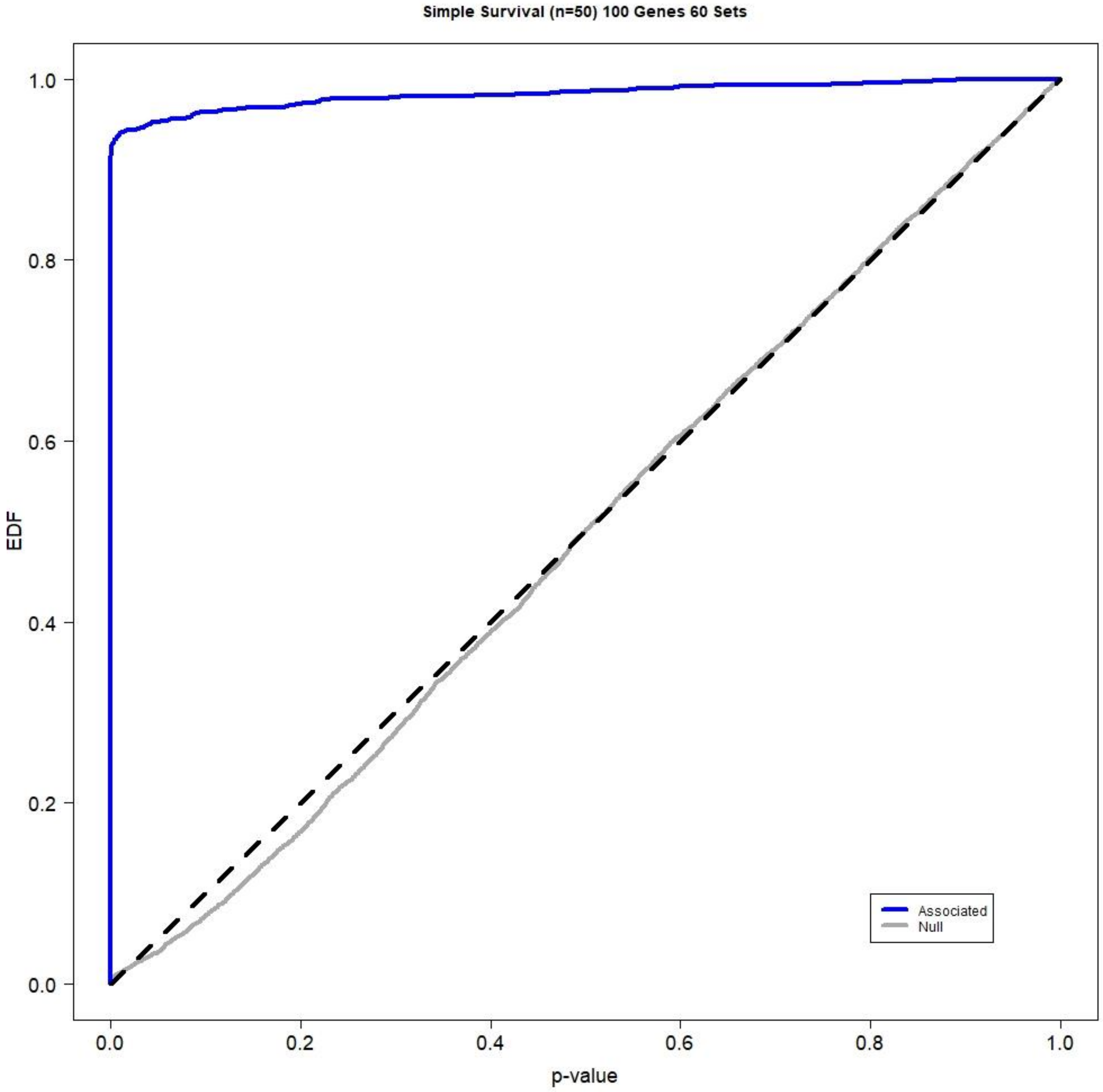

Simple Survival (n=50) 100 Genes 60 Sets
EDF
p-value
0.0
0.2
0.4
0.6
0.8
1.0
Associated
Null

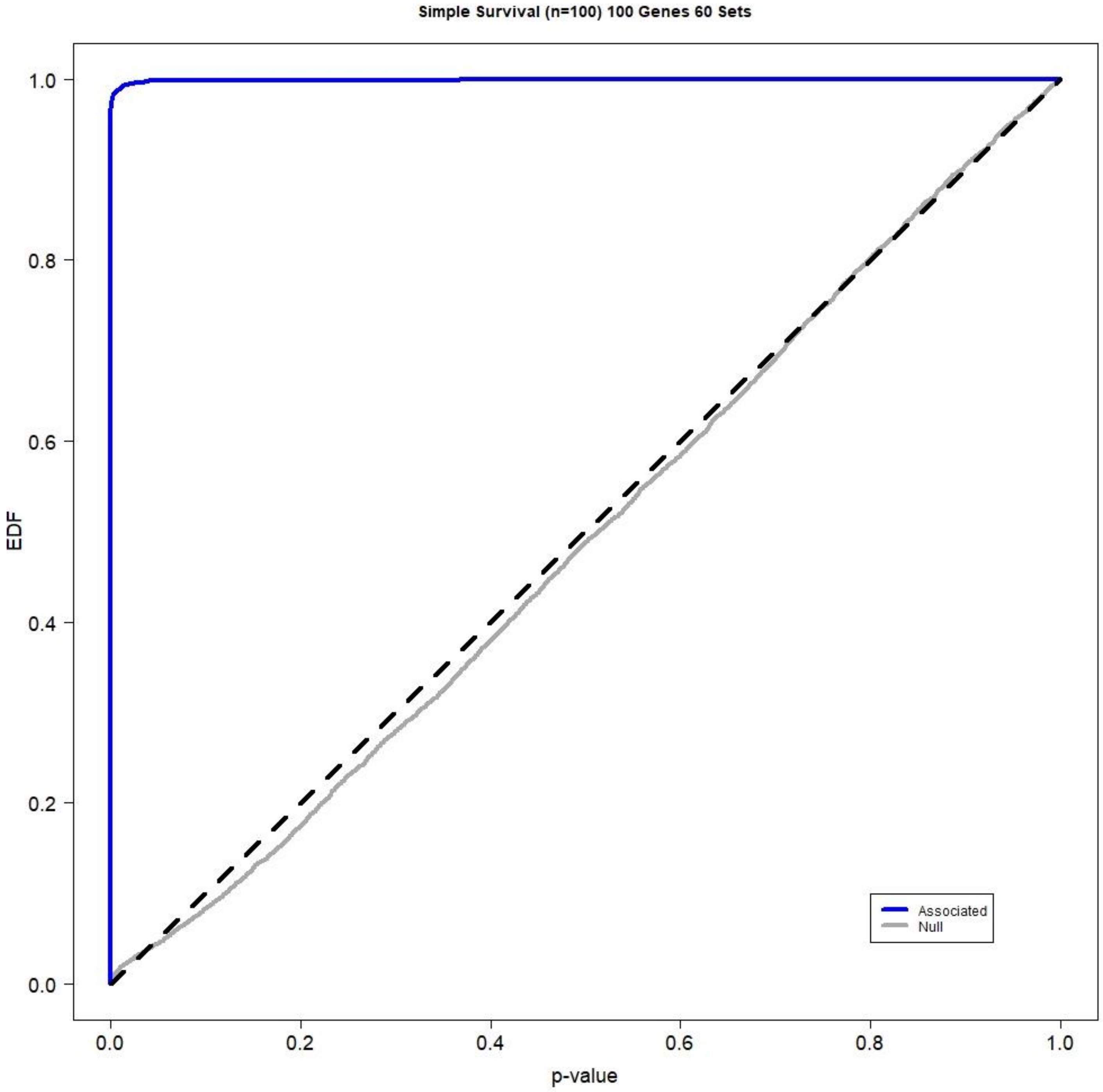
Simple Survival (n=100) 100 Genes 60 Sets
EDF
p-value
0.0
0.2
0.4
0.6
0.8
1.0
Associated
Null

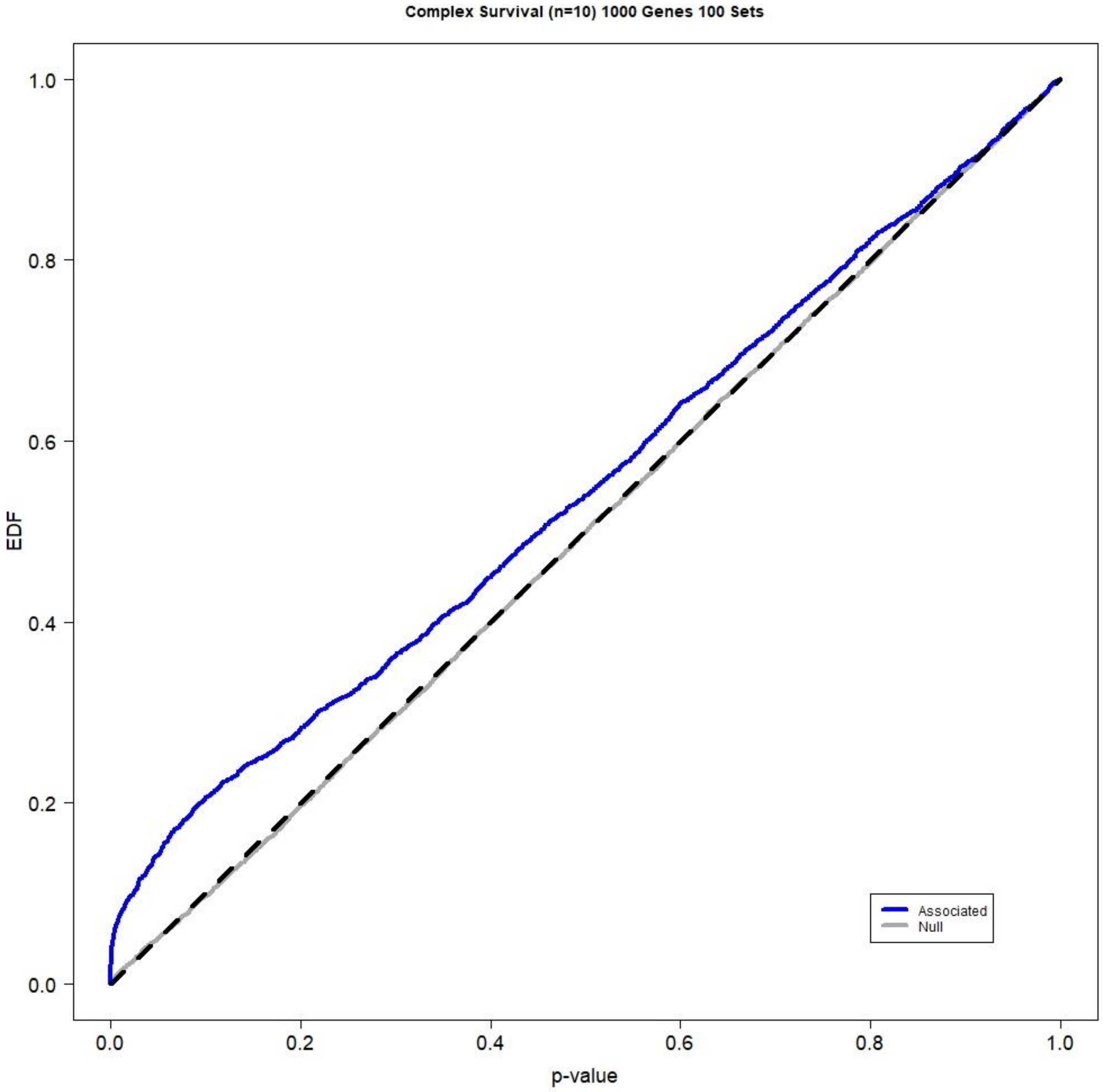
Complex Survival (n=10) 1000 Genes 100 Sets
EDF
p-value
0.0
0.2
0.4
0.6
0.8
1.0
Associated
Null

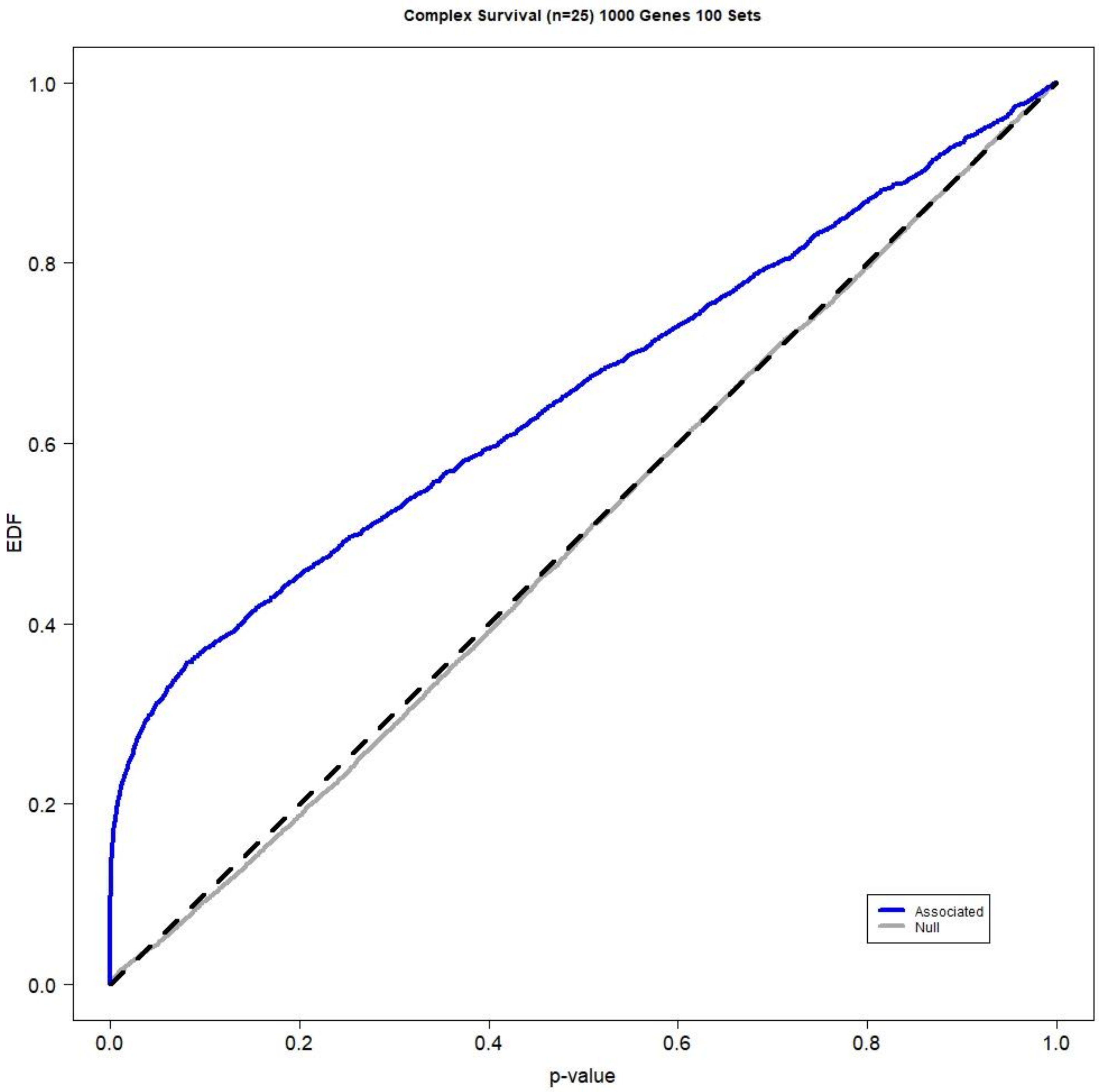

Complex Survival (n=25) 1000 Genes 100 Sets
EDF
p-value
0.0
0.2
0.4
0.6
0.8
1.0
Associated
Null

Complex Survival (n=50) 1000 Genes 100 Sets

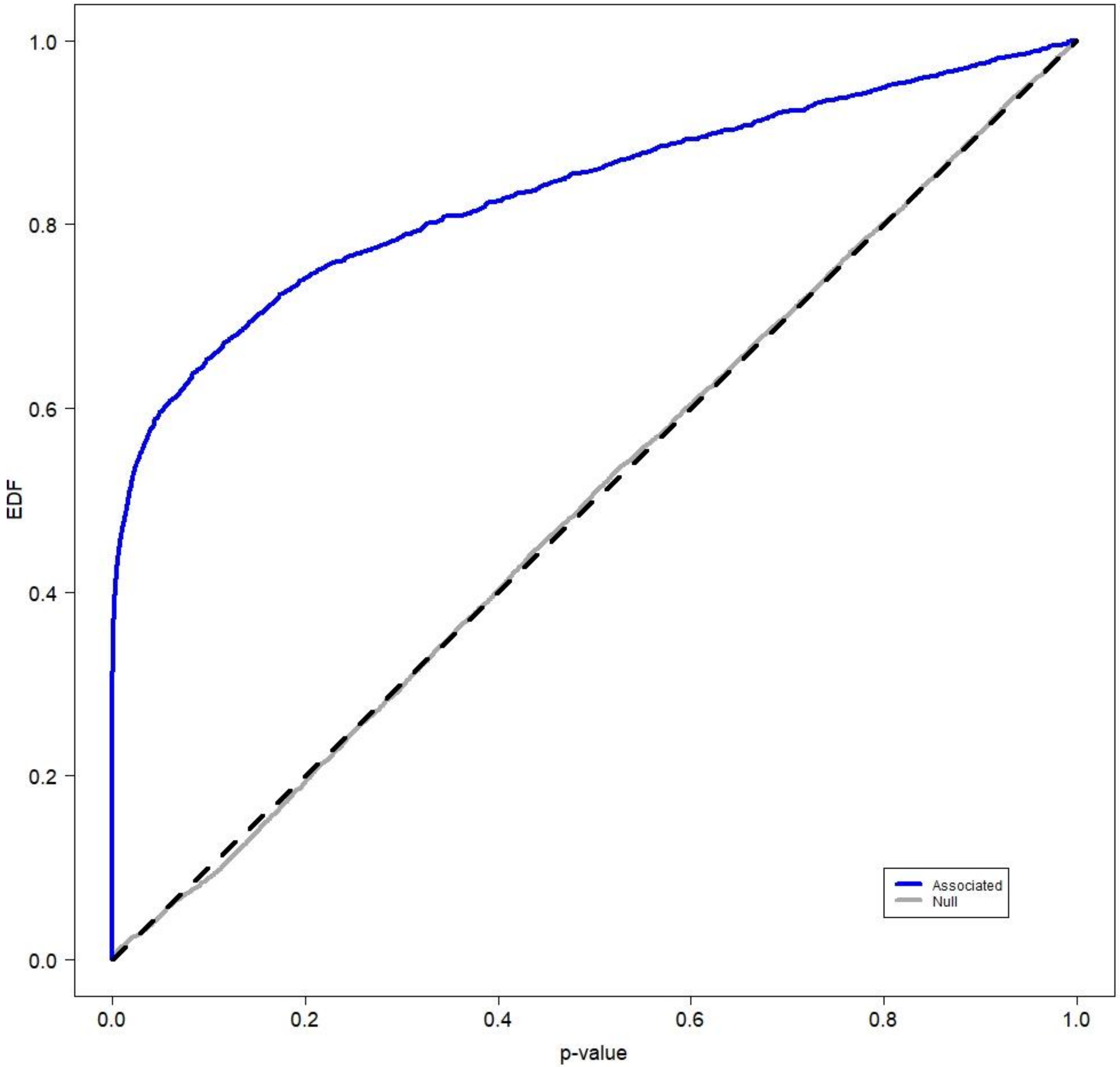

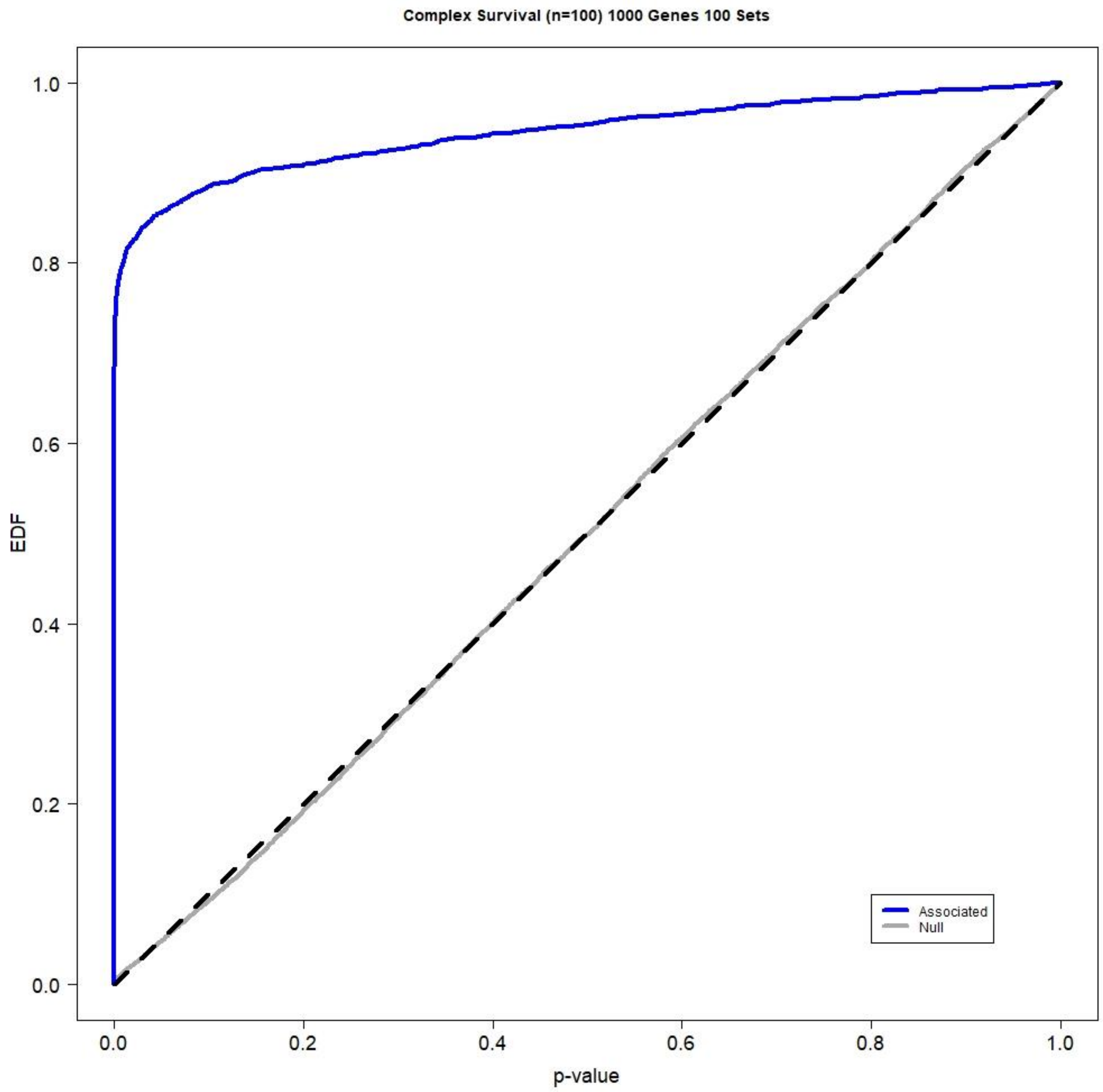
Complex Survival (n=100) 1000 Genes 100 Sets
1.0
0.8
0.6
0.4
0.2
0.0
EDF
0.0
0.2
0.4
0.6
0.8
1.0
p-value
Associated
Null

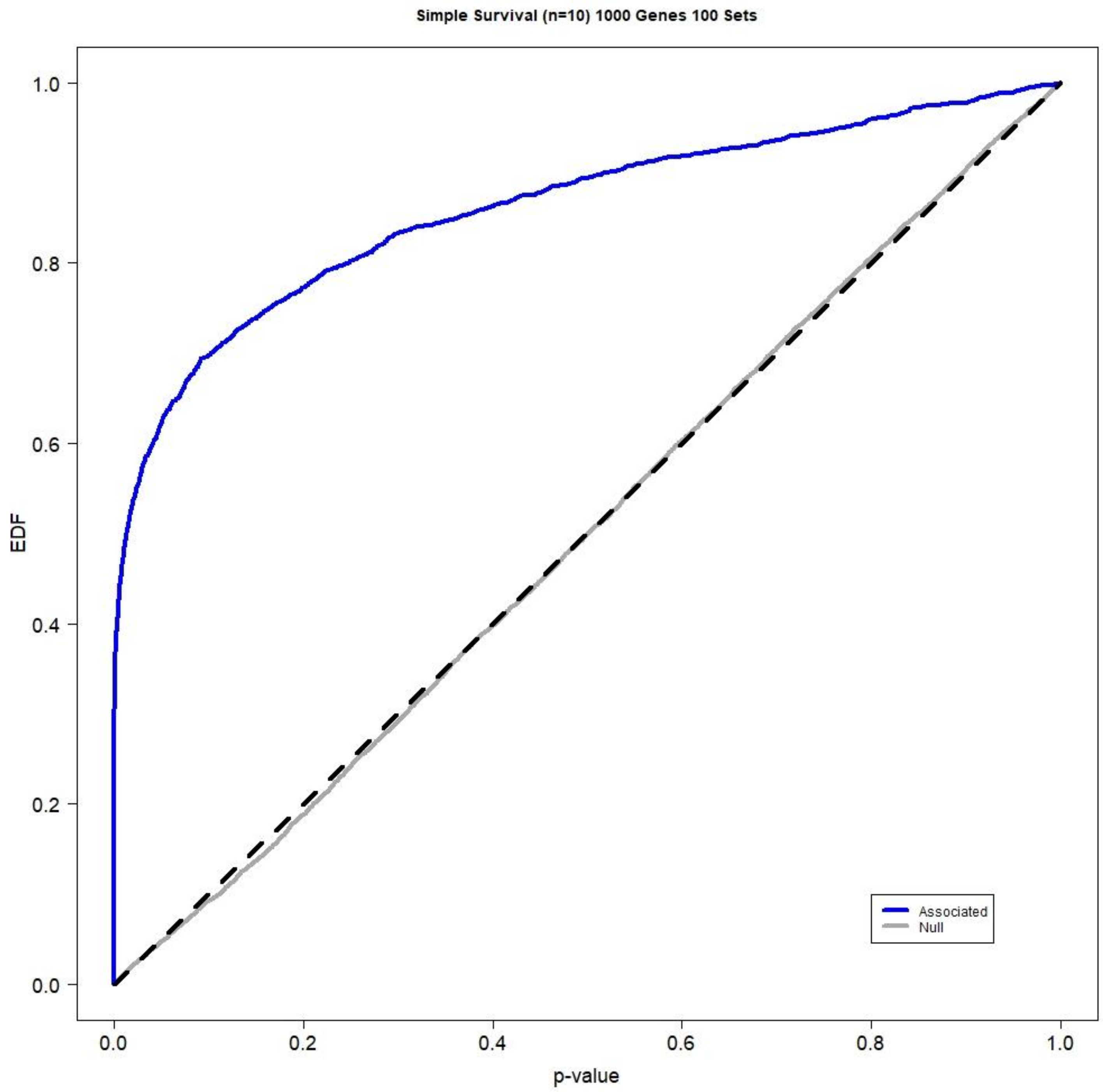
Simple Survival (n=10) 1000 Genes 100 Sets
EDF
p-value
0.0
0.2
0.4
0.6
0.8
1.0
Associated
Null

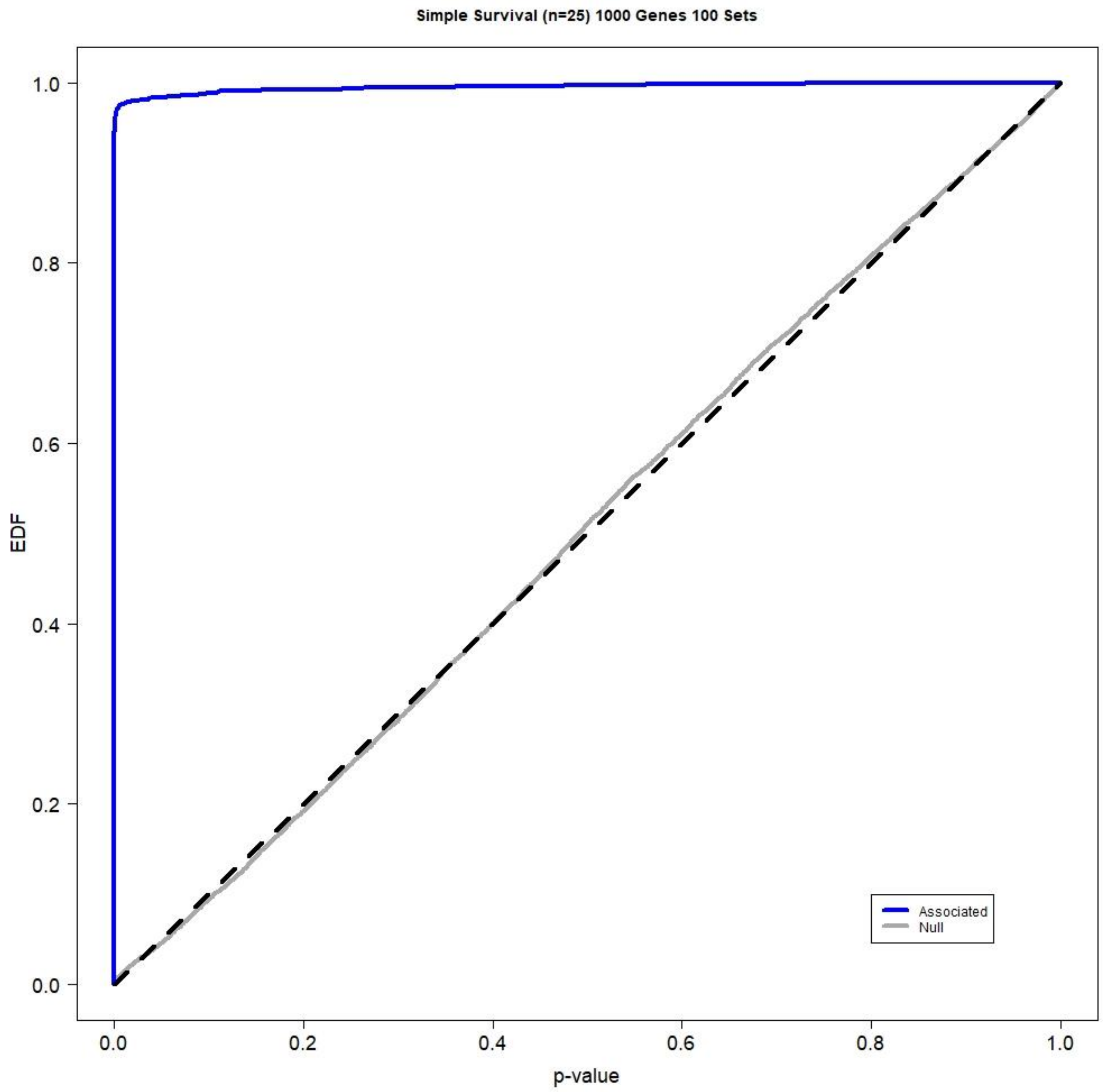
Simple Survival (n=25) 1000 Genes 100 Sets
EDF
p-value
0.0
0.2
0.4
0.6
0.8
1.0
Associated
Null

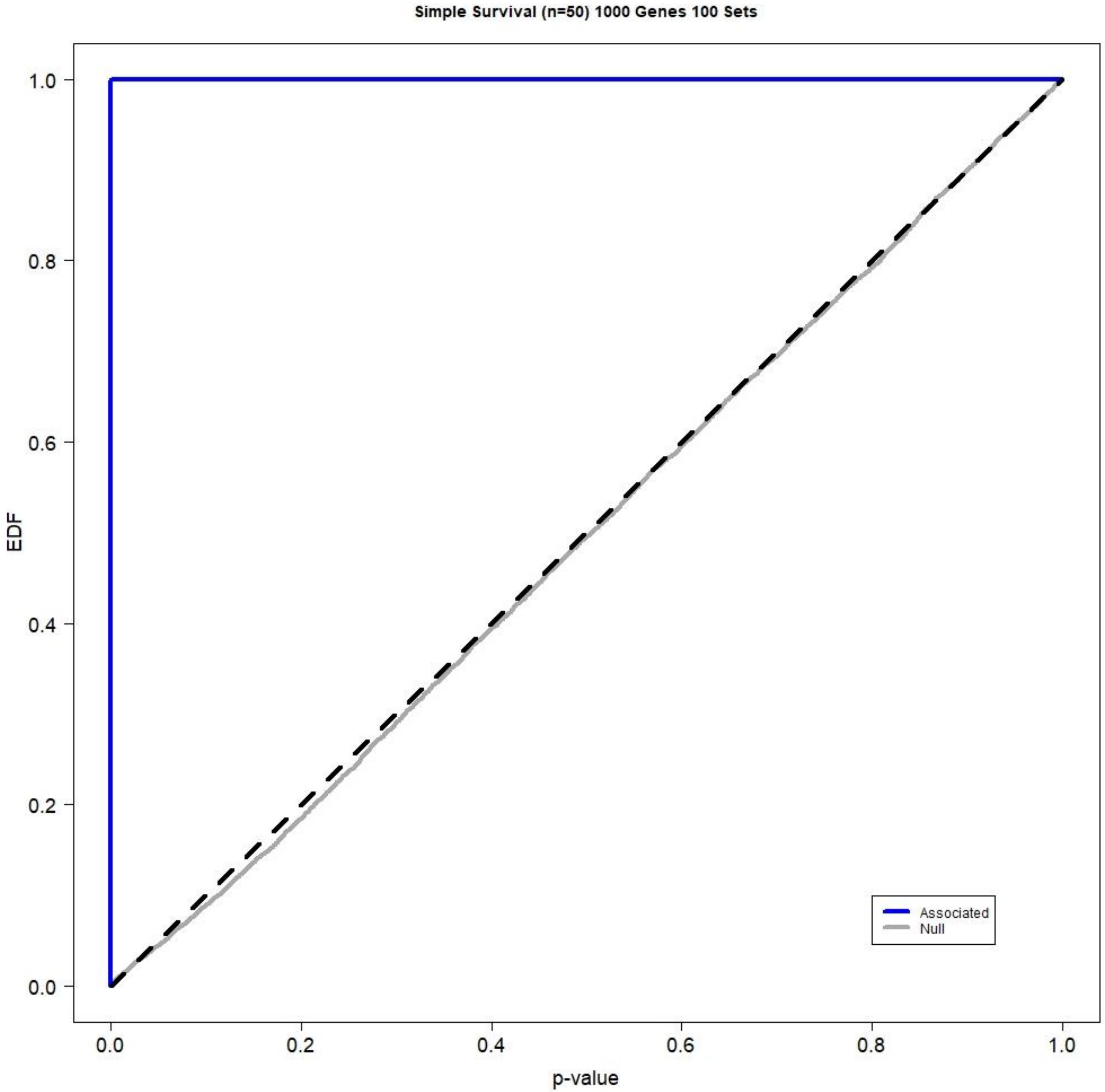
Simple Survival (n=50) 1000 Genes 100 Sets
EDF
p-value
0.0
0.2
0.4
0.6
0.8
1.0
Associated
Null

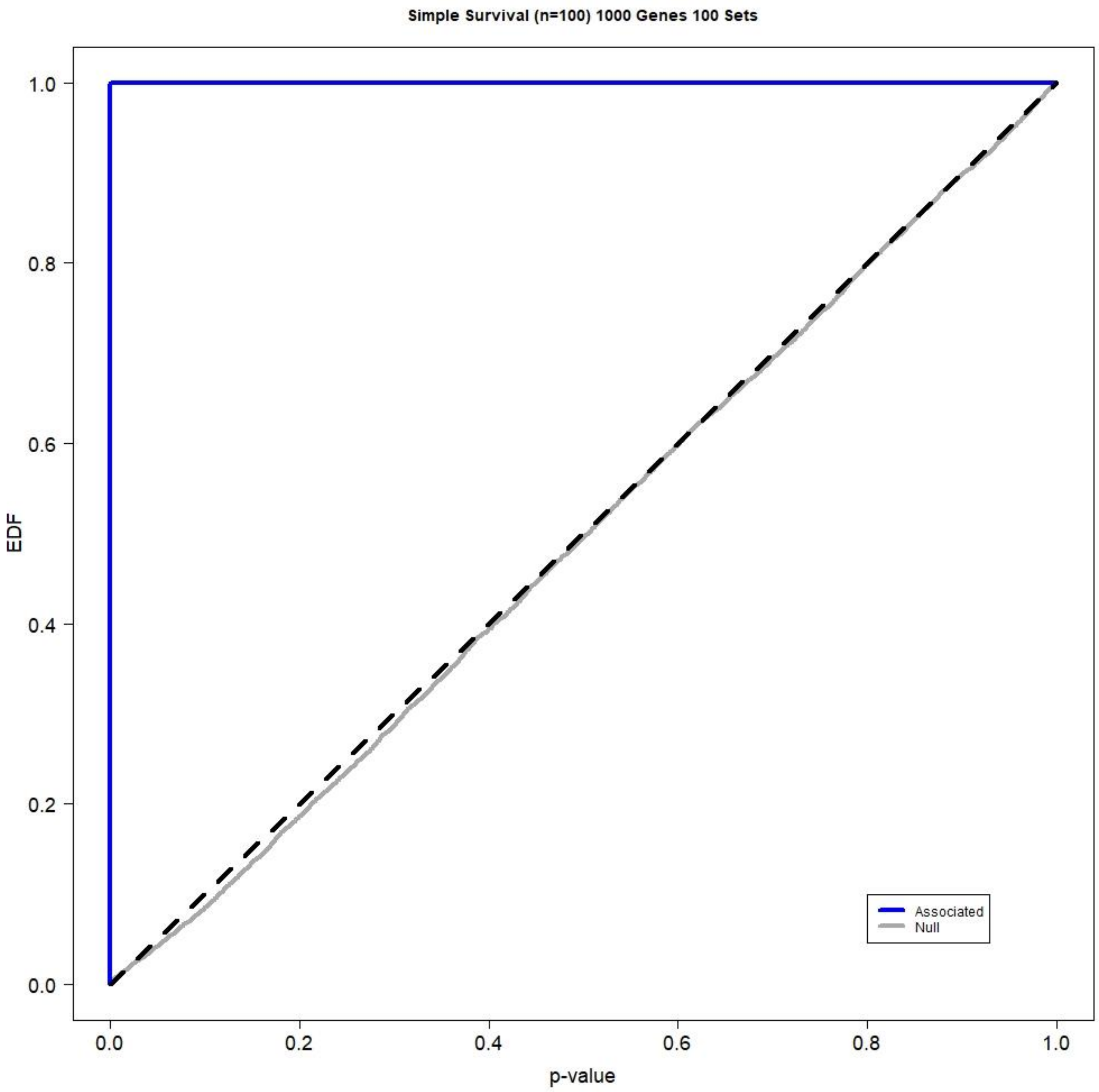

Simple Survival (n=100) 1000 Genes 100 Sets
1.0
0.8
0.6
0.4
0.2
0.0
EDF
0.0
0.2
0.4
0.6
0.8
1.0
p-value
Associated
Null
