# Supplementary material for: Gene-set distance analysis (GSDA): a powerful tool for gene-set association analysis
Source: BMC Bioinformatics. 2021 Apr 21;22:207. doi: 10.1186/s12859-021-04110-x (PMC8059024; doi:10.1186/s12859-021-04110-x)
Supplement: Supplementary file 3 — Additional file 3. This supplementary file provides box plots of power and type I error rates at 5%nominal level for each scenario. [file 12859_2021_4110_MOESM3_ESM.pdf]

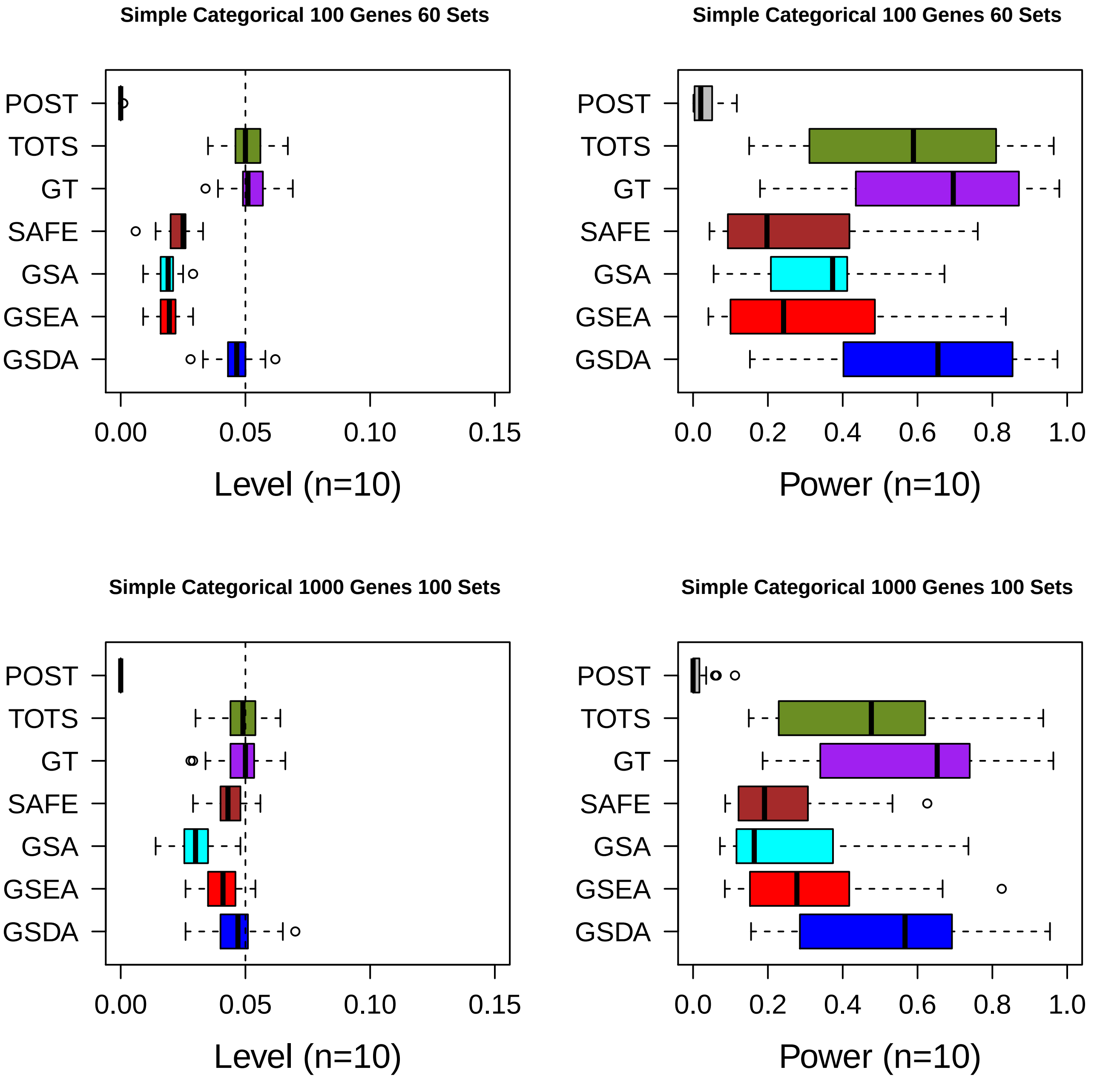

Simple Categorical 100 Genes 60 Sets
POST
TOTS
GT
SAFE
GSA
GSEA
GSDA
0.00
0.05
0.10
0.15
Level (n=10)
Simple Categorical 100 Genes 60 Sets
0.0
0.2
0.4
0.6
0.8
1.0
Power (n=10)
Simple Categorical 1000 Genes 100 Sets
Level (n=10)
Simple Categorical 1000 Genes 100 Sets
Power (n=10)

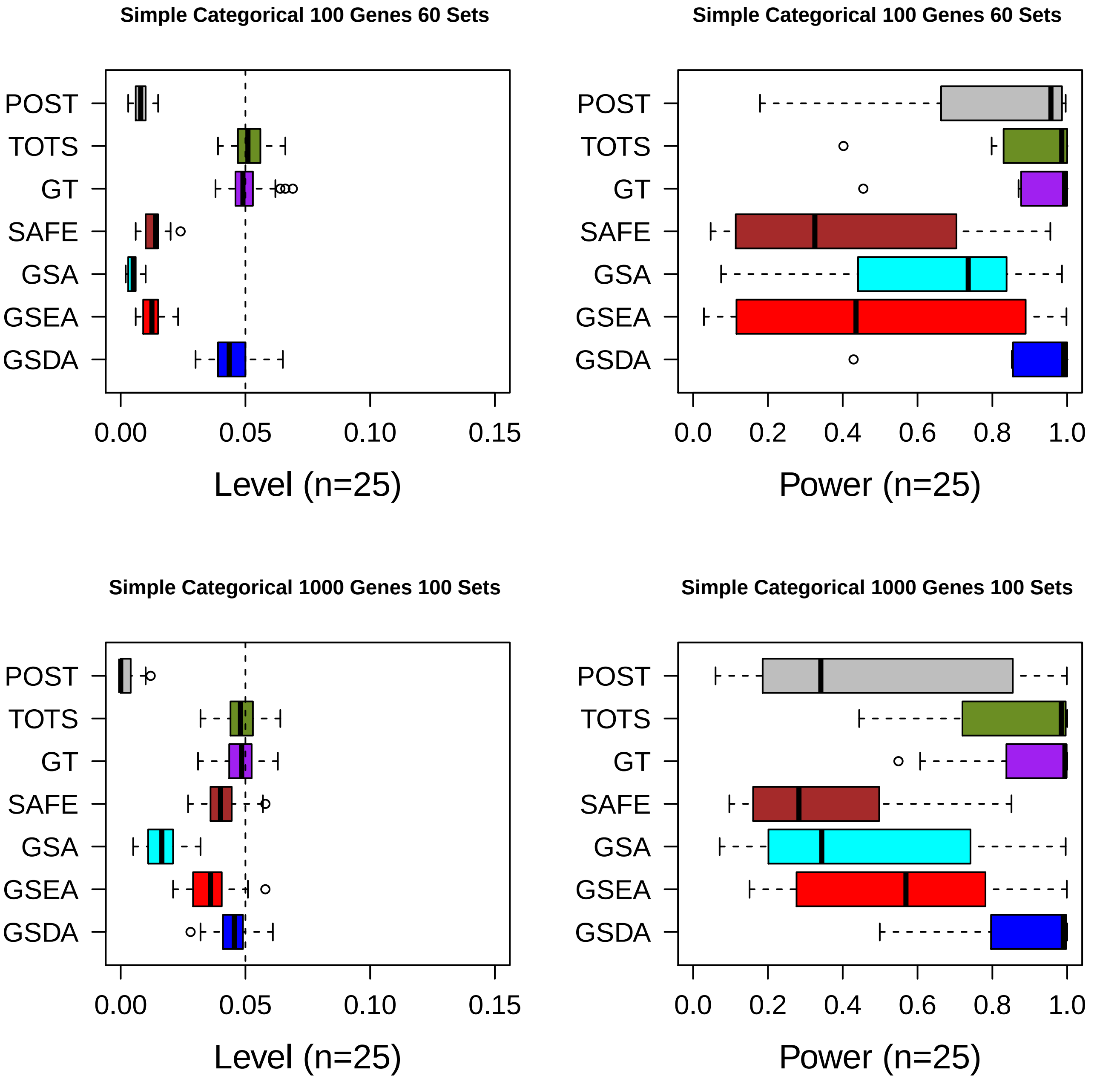

Simple Categorical 100 Genes 60 Sets
POST
TOTS
GT
SAFE
GSA
GSEA
GSDA
0.00
0.05
0.10
0.15
Level (n=25)
Simple Categorical 100 Genes 60 Sets
0.0
0.2
0.4
0.6
0.8
1.0
Power (n=25)
Simple Categorical 1000 Genes 100 Sets
Level (n=25)
Simple Categorical 1000 Genes 100 Sets
Power (n=25)

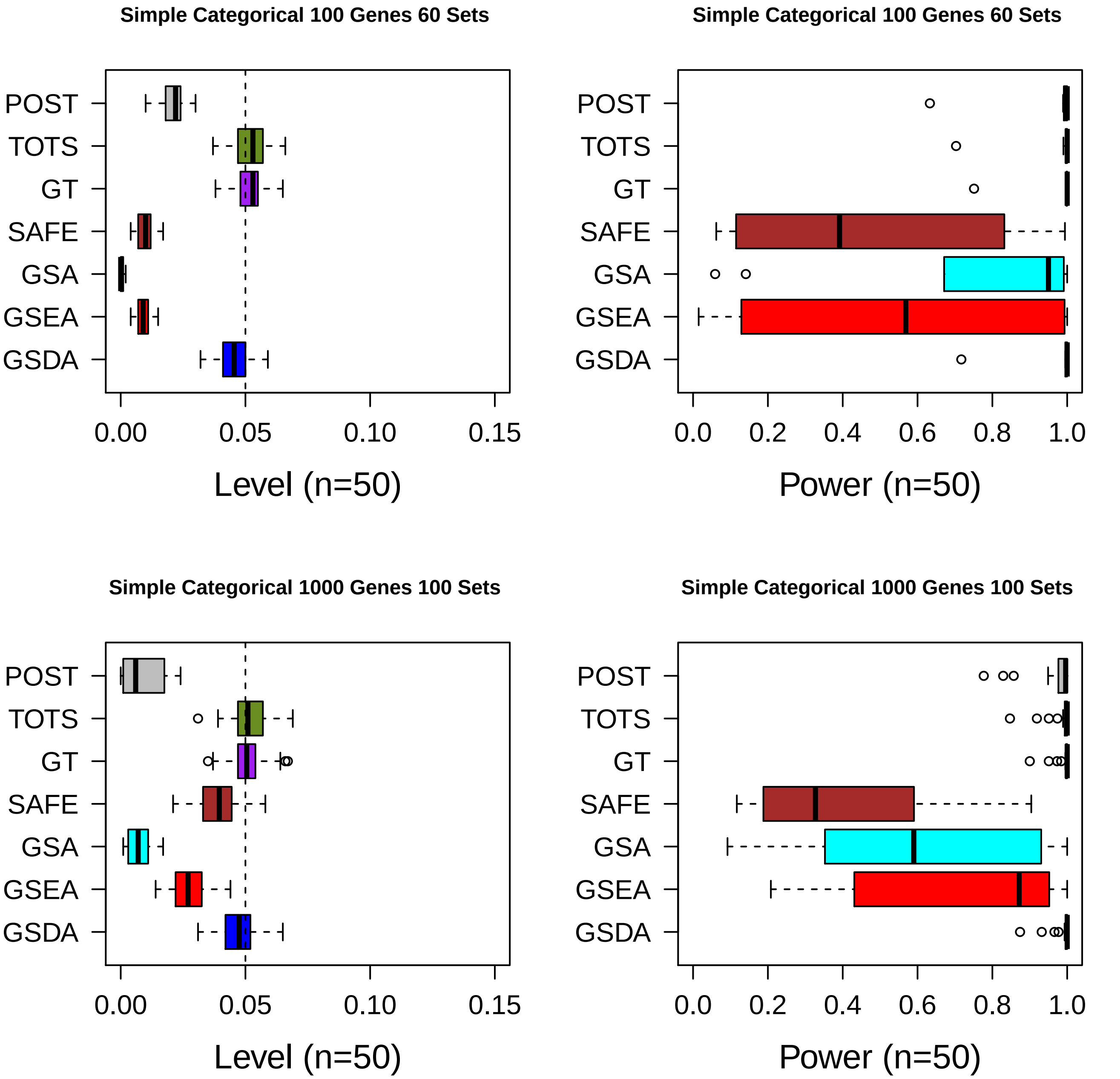
Simple Categorical 100 Genes 60 Sets
POST
TOTS
GT
SAFE
GSA
GSEA
GSDA
0.00
0.05
0.10
0.15
Level (n=50)
Simple Categorical 100 Genes 60 Sets
0.0
0.2
0.4
0.6
0.8
1.0
Power (n=50)
Simple Categorical 1000 Genes 100 Sets
Level (n=50)
Simple Categorical 1000 Genes 100 Sets
Power (n=50)

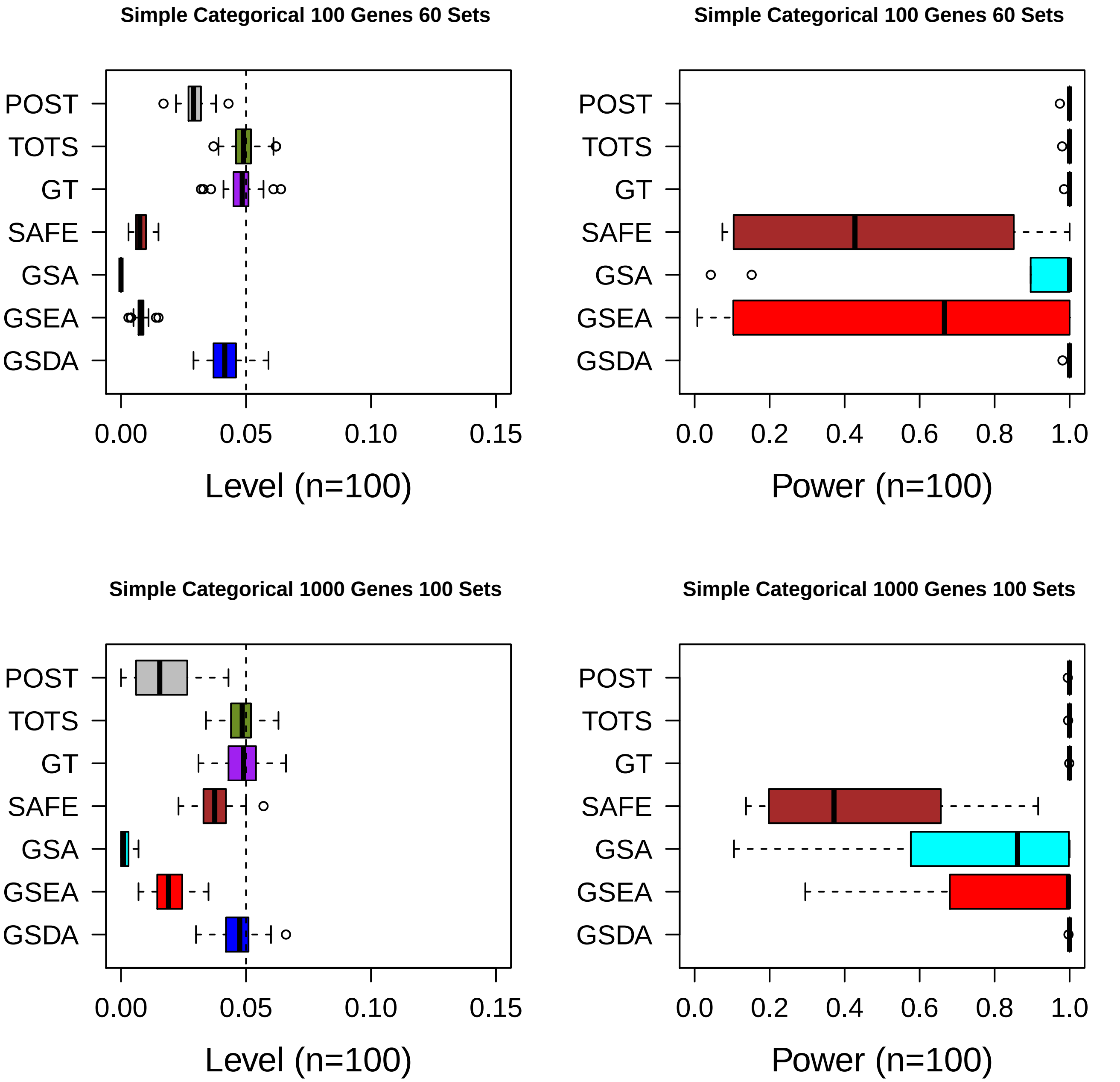
Simple Categorical 100 Genes 60 Sets
POST
TOTS
GT
SAFE
GSA
GSEA
GSDA
0.00
0.05
0.10
0.15
Level (n=100)
Simple Categorical 100 Genes 60 Sets
POST
TOTS
GT
SAFE
GSA
GSEA
GSDA
0.0
0.2
0.4
0.6
0.8
1.0
Power (n=100)
Simple Categorical 1000 Genes 100 Sets
POST
TOTS
GT
SAFE
GSA
GSEA
GSDA
0.00
0.05
0.10
0.15
Level (n=100)
Simple Categorical 1000 Genes 100 Sets
POST
TOTS
GT
SAFE
GSA
GSEA
GSDA
0.0
0.2
0.4
0.6
0.8
1.0
Power (n=100)

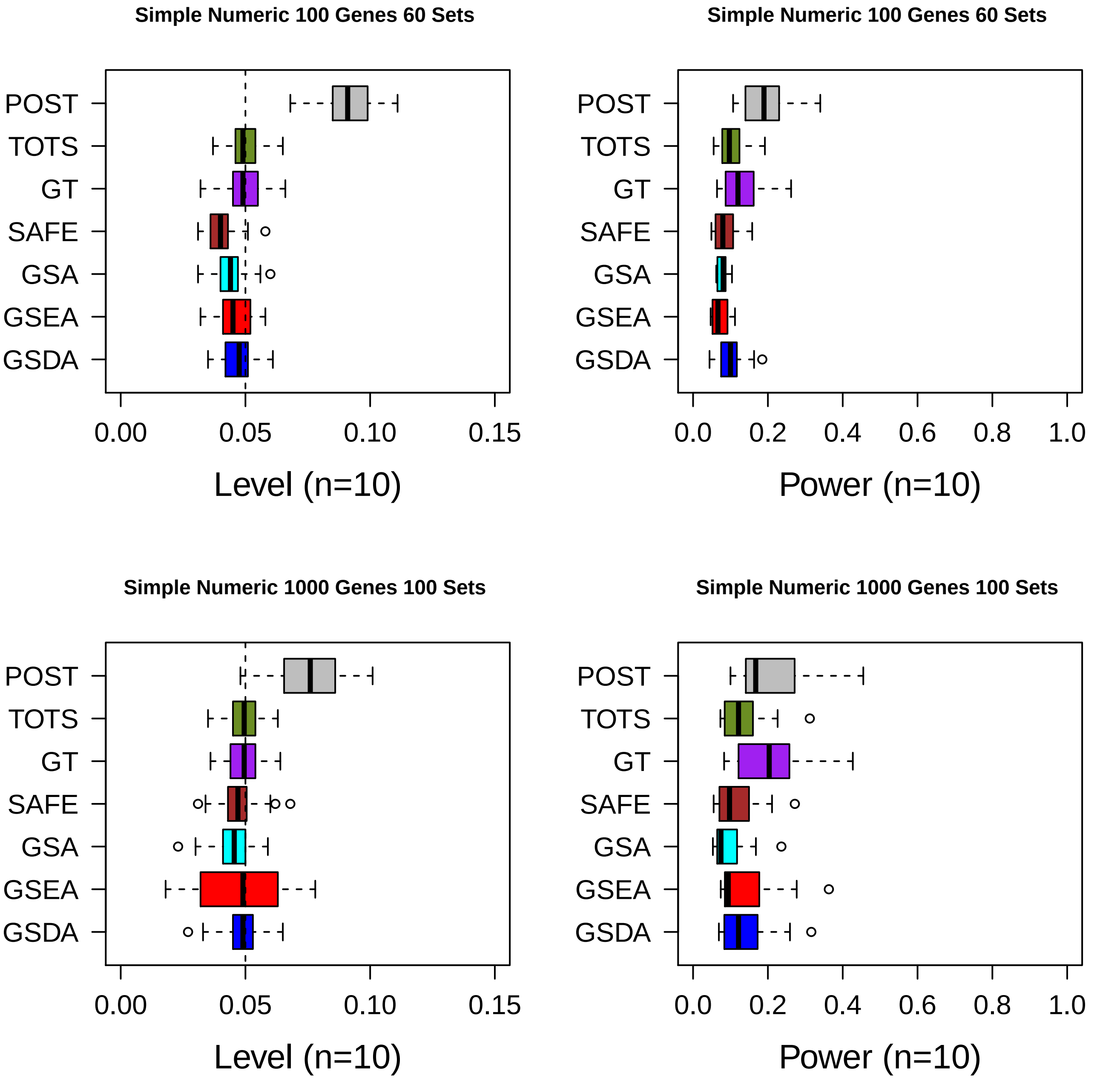
Simple Numeric 100 Genes 60 Sets
POST
TOTS
GT
SAFE
GSA
GSEA
GSDA
0.00
0.05
0.10
0.15
Level (n=10)
Simple Numeric 100 Genes 60 Sets
0.0
0.2
0.4
0.6
0.8
1.0
Power (n=10)
Simple Numeric 1000 Genes 100 Sets
Level (n=10)
Simple Numeric 1000 Genes 100 Sets
Power (n=10)

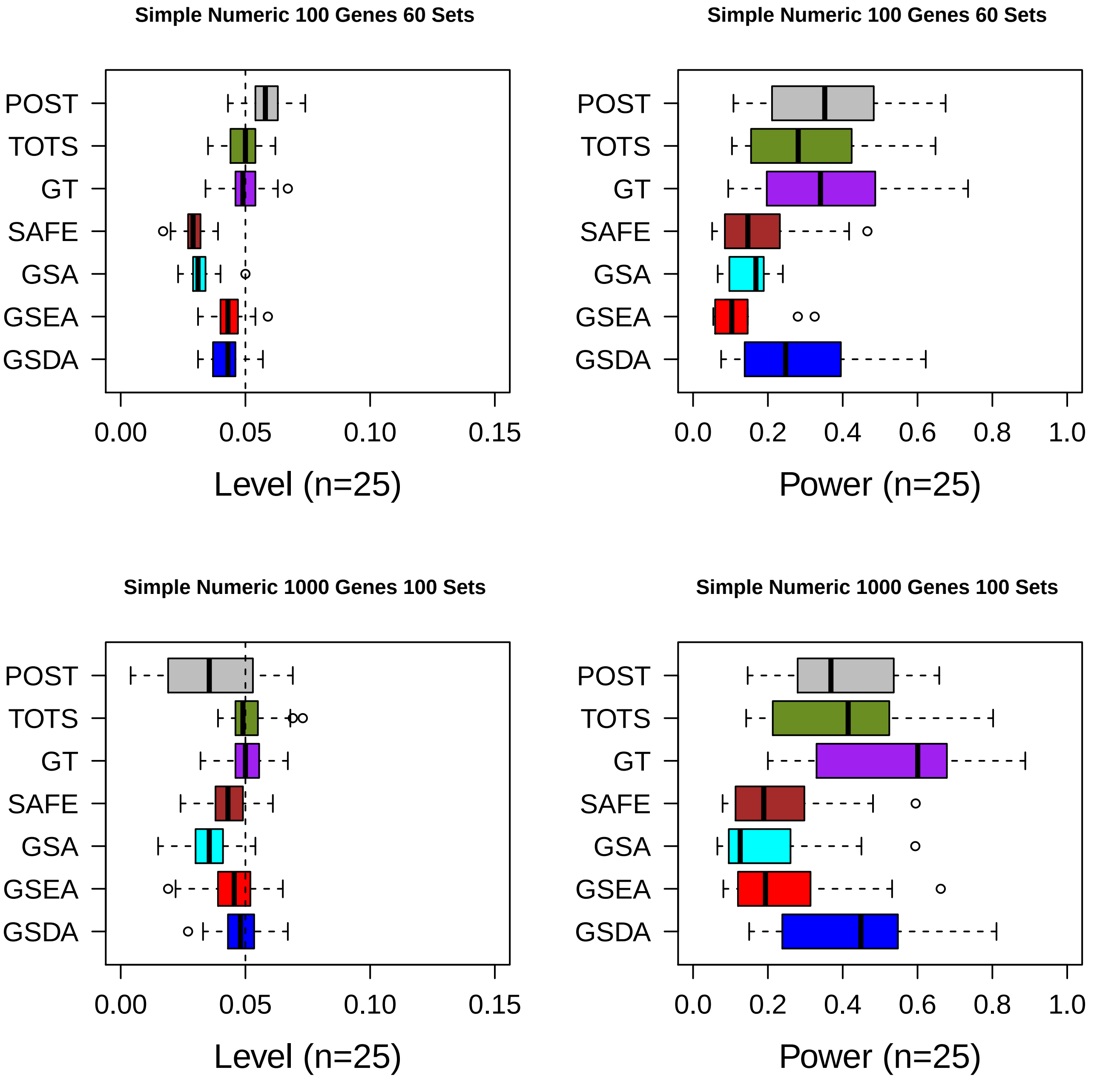
Simple Numeric 100 Genes 60 Sets
Simple Numeric 100 Genes 60 Sets
POST
TOTS
GT
SAFE
GSA
GSEA
GSDA
0.00
0.05
0.10
0.15
Level (n=25)
0.0
0.2
0.4
0.6
0.8
1.0
Power (n=25)
Simple Numeric 1000 Genes 100 Sets
Simple Numeric 1000 Genes 100 Sets
Level (n=25)
Power (n=25)

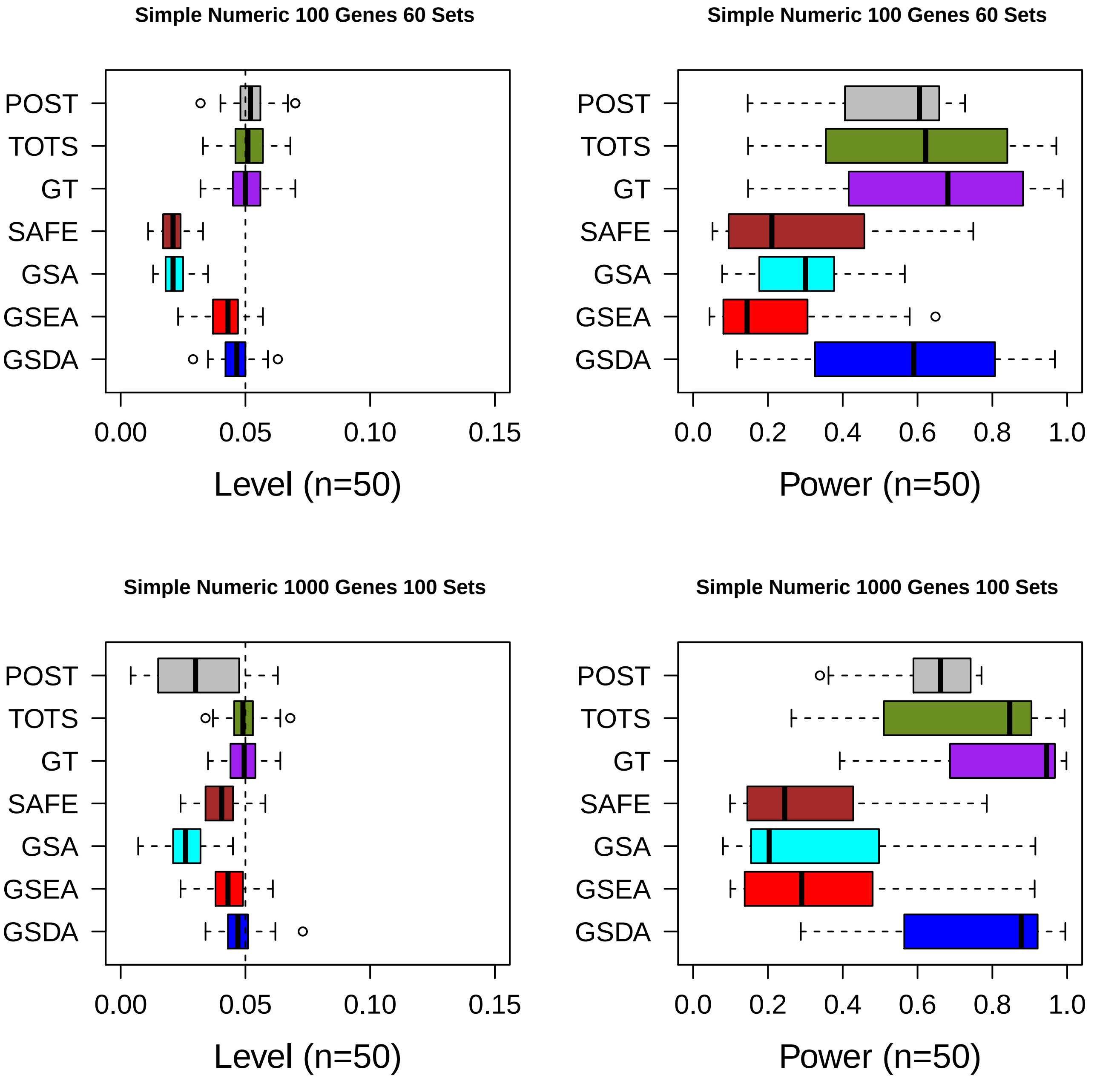
Simple Numeric 100 Genes 60 Sets
POST
TOTS
GT
SAFE
GSA
GSEA
GSDA
0.00
0.05
0.10
0.15
Level (n=50)
Simple Numeric 100 Genes 60 Sets
0.0
0.2
0.4
0.6
0.8
1.0
Power (n=50)
Simple Numeric 1000 Genes 100 Sets
Level (n=50)
Simple Numeric 1000 Genes 100 Sets
Power (n=50)

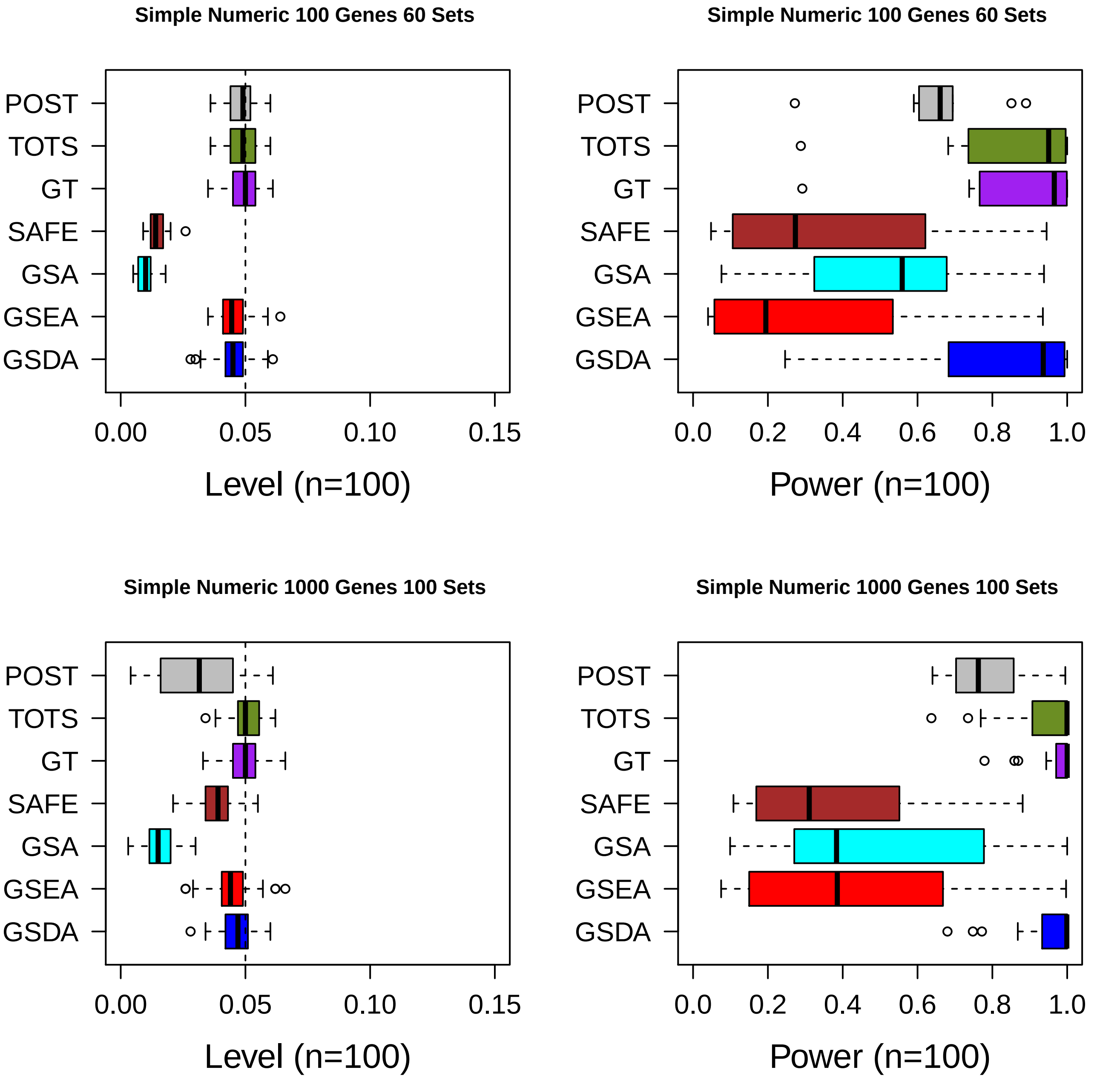
Simple Numeric 100 Genes 60 Sets
POST
TOTS
GT
SAFE
GSA
GSEA
GSDA
0.00
0.05
0.10
0.15
Level (n=100)
Simple Numeric 100 Genes 60 Sets
0.0
0.2
0.4
0.6
0.8
1.0
Power (n=100)
Simple Numeric 1000 Genes 100 Sets
Level (n=100)
Simple Numeric 1000 Genes 100 Sets
Power (n=100)

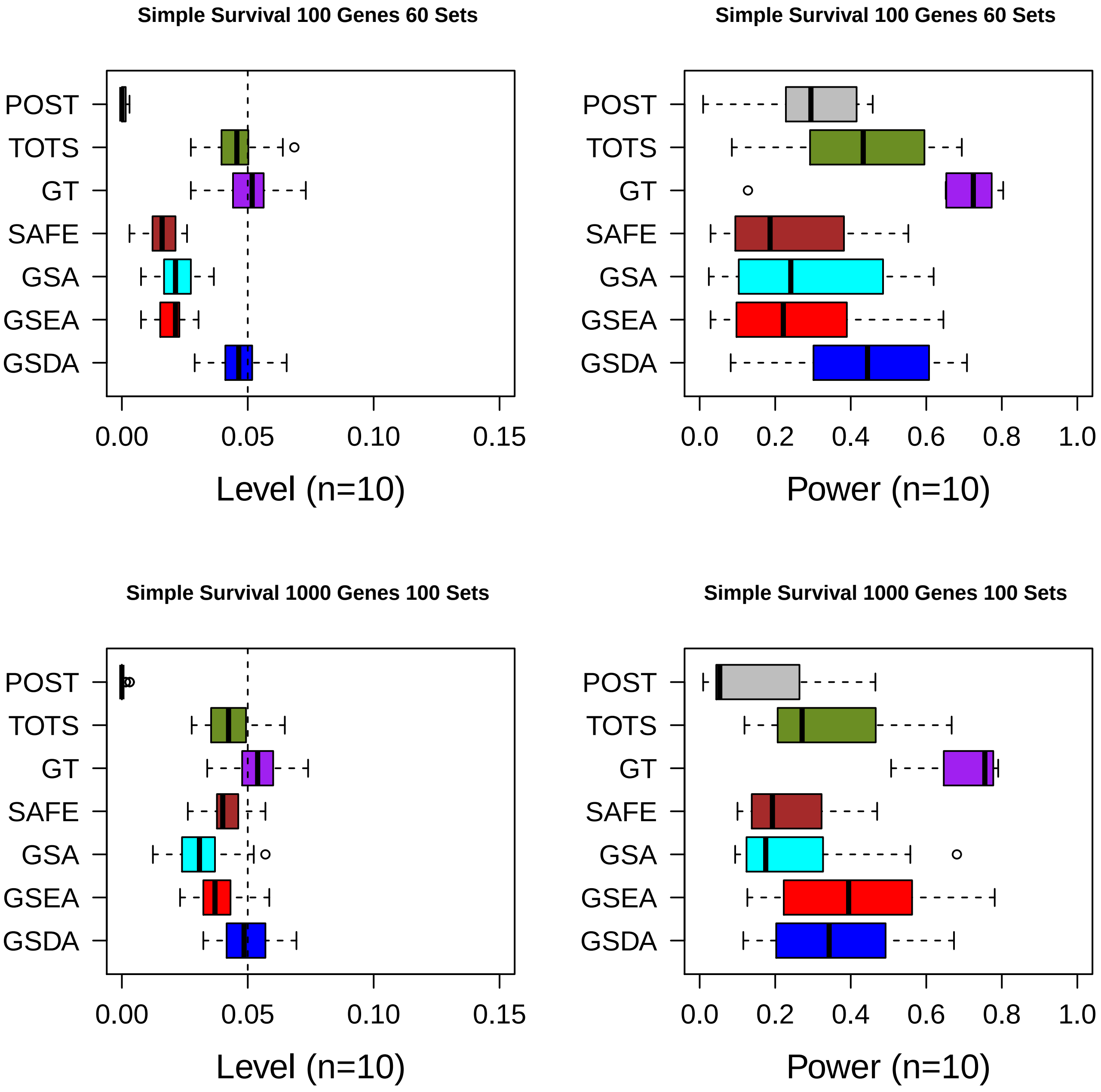
Simple Survival 100 Genes 60 Sets
POST
TOTS
GT
SAFE
GSA
GSEA
GSDA
0.00
0.05
0.10
0.15
Level (n=10)
Simple Survival 100 Genes 60 Sets
0.0
0.2
0.4
0.6
0.8
1.0
Power (n=10)
Simple Survival 1000 Genes 100 Sets
Level (n=10)
Simple Survival 1000 Genes 100 Sets
Power (n=10)

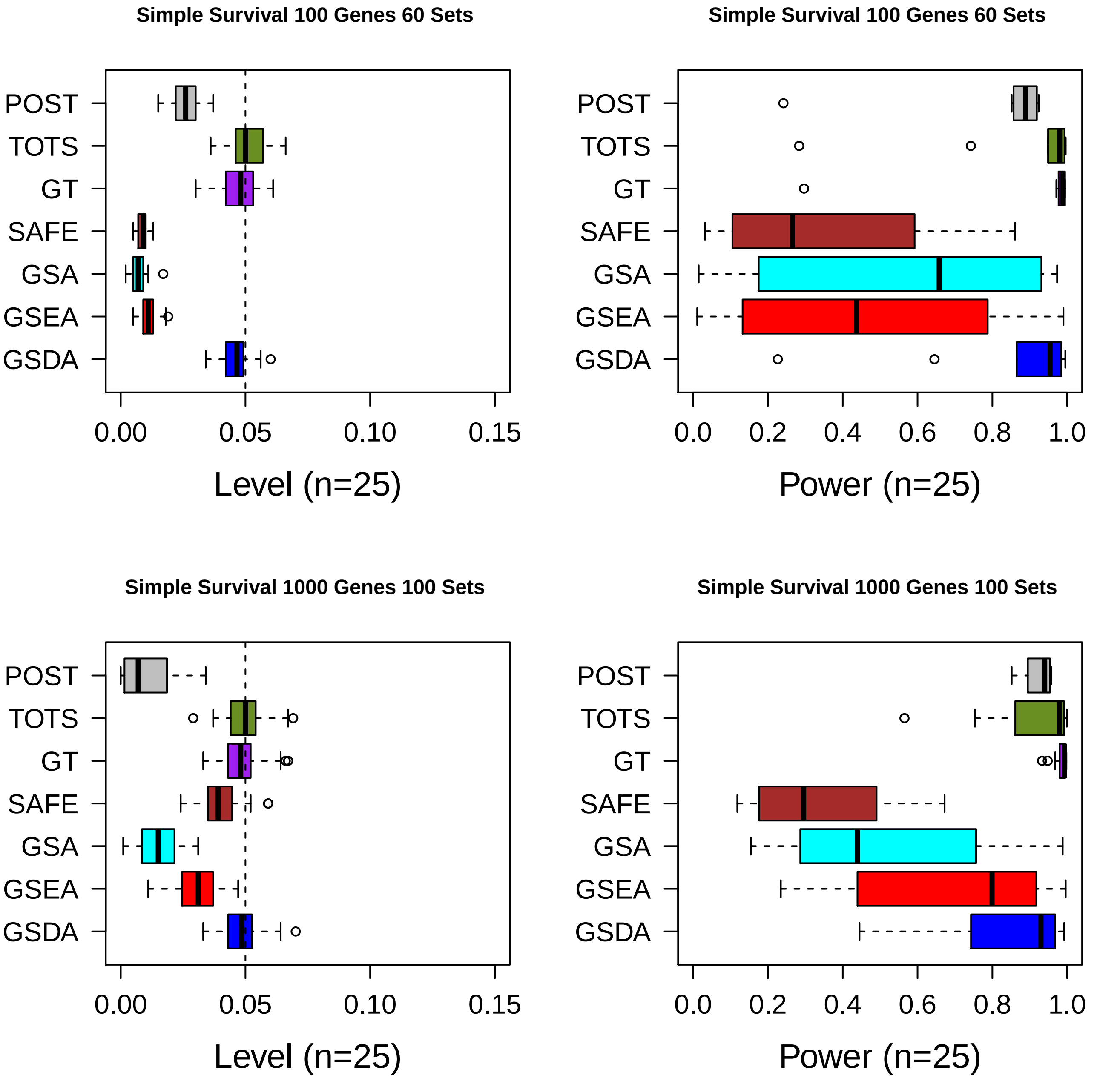

Simple Survival 100 Genes 60 Sets
POST
TOTS
GT
SAFE
GSA
GSEA
GSDA
0.00
0.05
0.10
0.15
Level (n=25)
Simple Survival 100 Genes 60 Sets
0.0
0.2
0.4
0.6
0.8
1.0
Power (n=25)
Simple Survival 1000 Genes 100 Sets
Level (n=25)
Simple Survival 1000 Genes 100 Sets
Power (n=25)

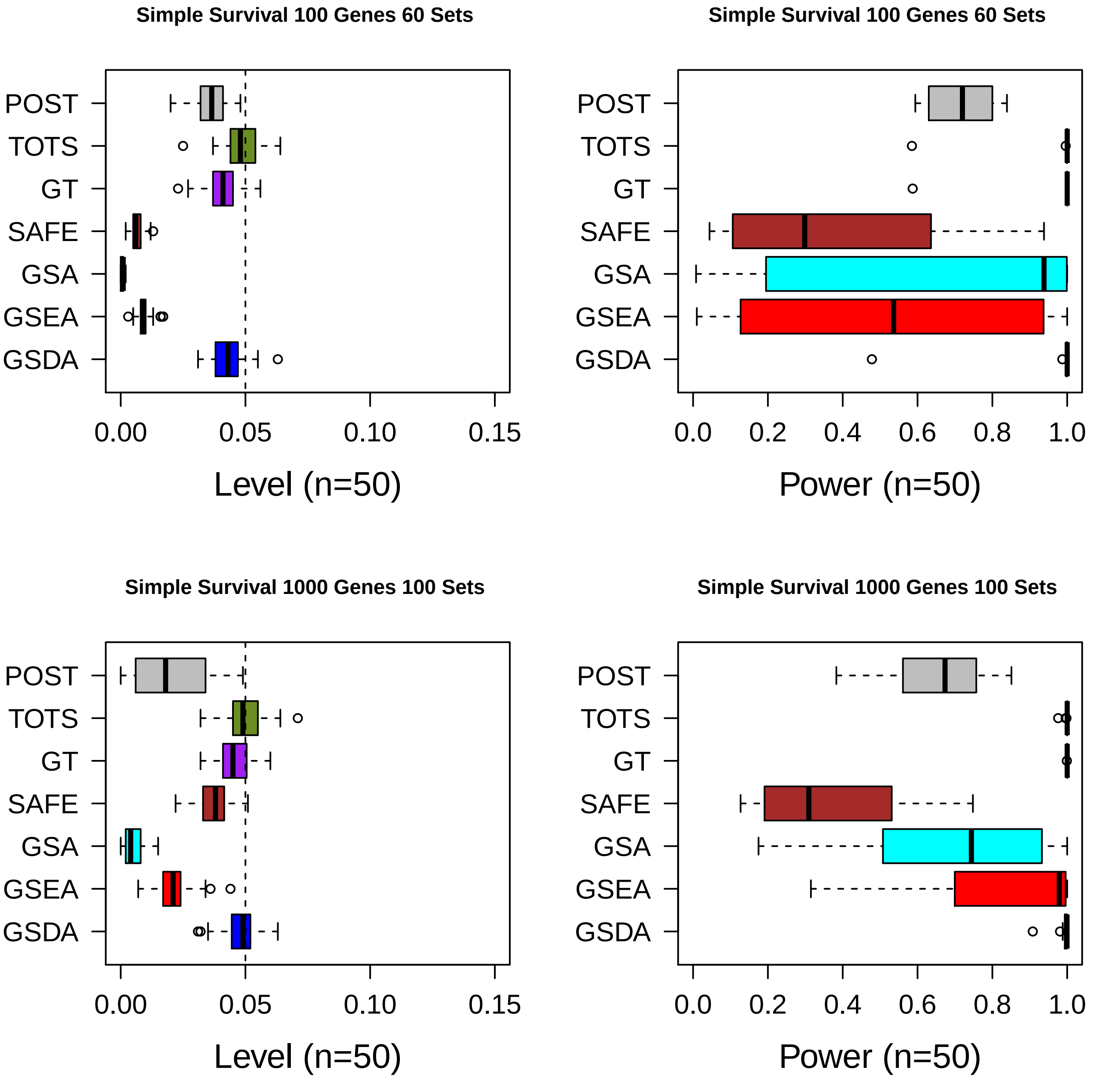
Simple Survival 100 Genes 60 Sets
POST
TOTS
GT
SAFE
GSA
GSEA
GSDA
0.00
0.05
0.10
0.15
Level (n=50)
Simple Survival 100 Genes 60 Sets
0.0
0.2
0.4
0.6
0.8
1.0
Power (n=50)
Simple Survival 1000 Genes 100 Sets
Level (n=50)
Simple Survival 1000 Genes 100 Sets
Power (n=50)

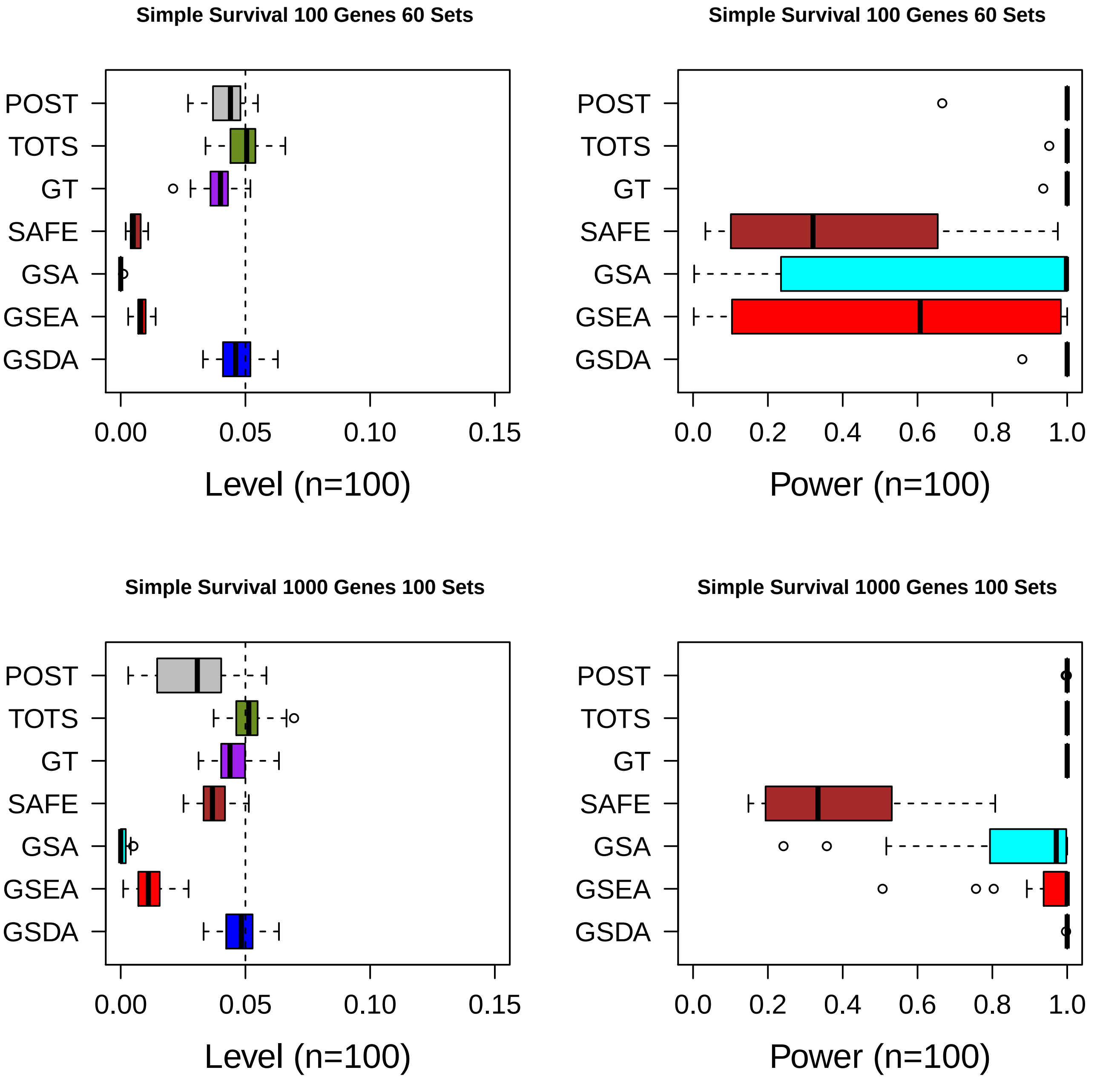
Simple Survival 100 Genes 60 Sets
POST
TOTS
GT
SAFE
GSA
GSEA
GSDA
0.00
0.05
0.10
0.15
Level (n=100)
Simple Survival 100 Genes 60 Sets
0.0
0.2
0.4
0.6
0.8
1.0
Power (n=100)
Simple Survival 1000 Genes 100 Sets
Level (n=100)
Simple Survival 1000 Genes 100 Sets
Power (n=100)

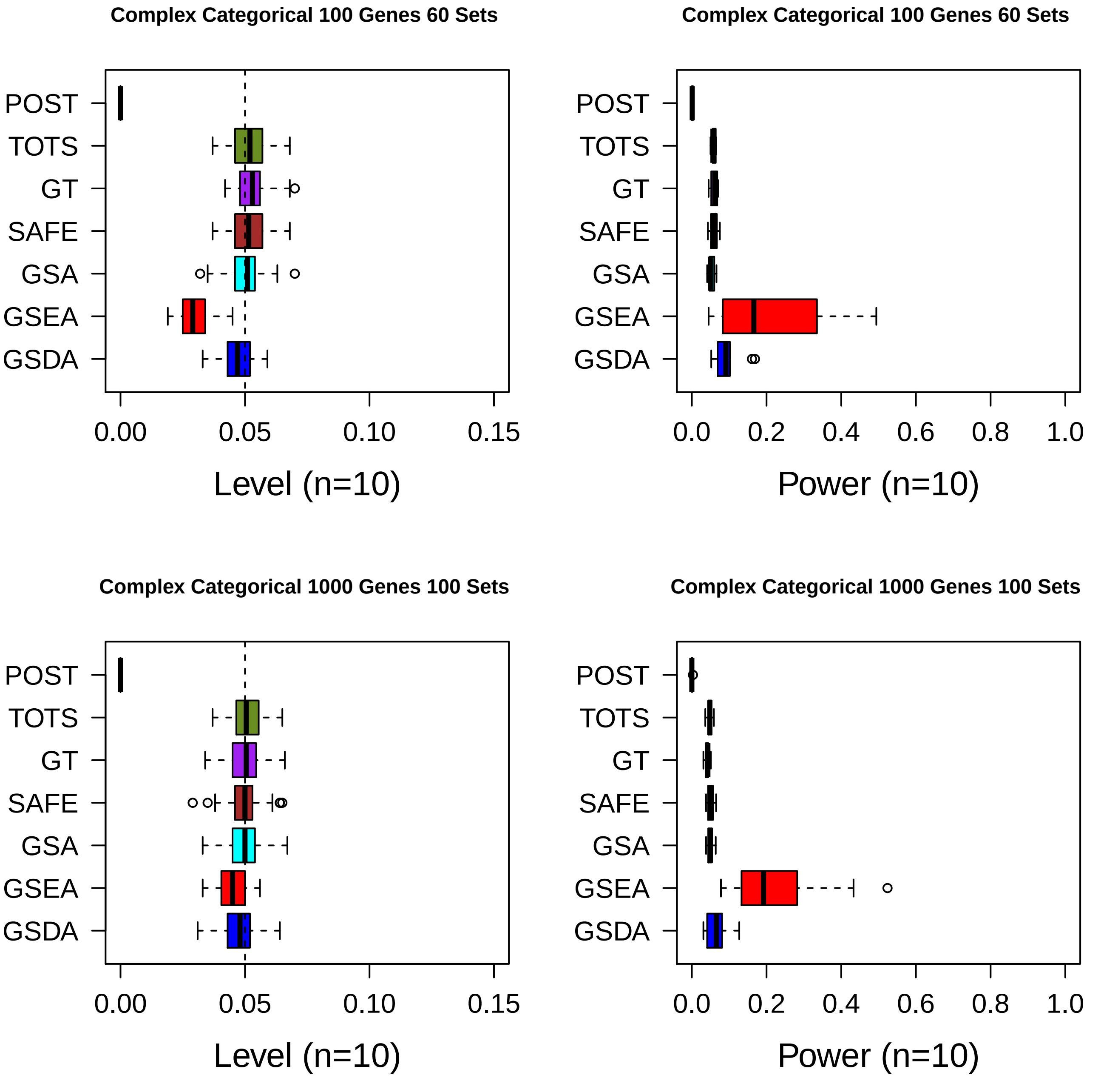

Complex Categorical 100 Genes 60 Sets
POST
TOTS
GT
SAFE
GSA
GSEA
GSDA
0.00
0.05
0.10
0.15
Level (n=10)
Complex Categorical 100 Genes 60 Sets
0.0
0.2
0.4
0.6
0.8
1.0
Power (n=10)
Complex Categorical 1000 Genes 100 Sets
Level (n=10)
Complex Categorical 1000 Genes 100 Sets
Power (n=10)

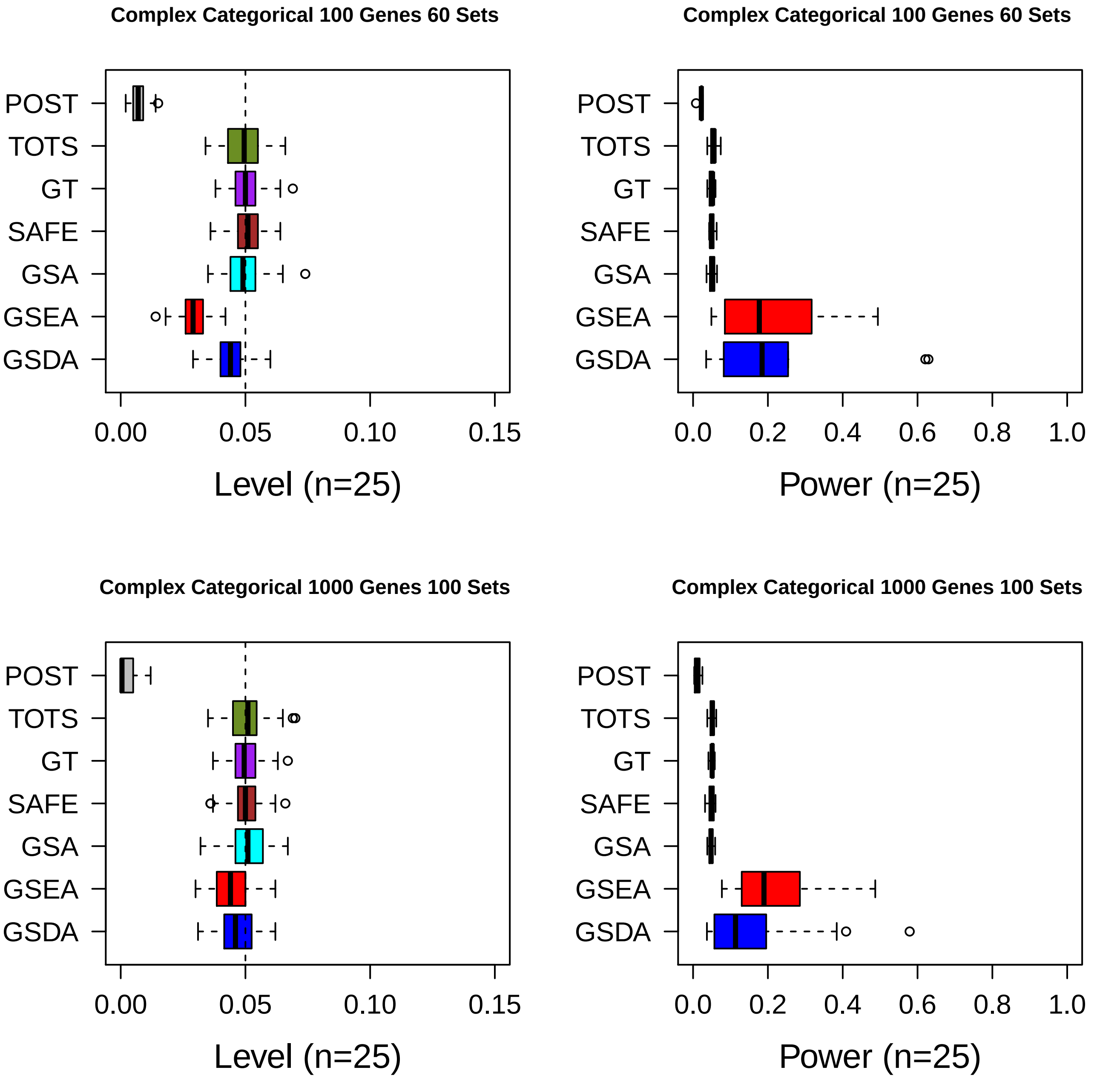
Complex Categorical 100 Genes 60 Sets
POST
TOTS
GT
SAFE
GSA
GSEA
GSDA
0.00
0.05
0.10
0.15
Level (n=25)
Complex Categorical 100 Genes 60 Sets
0.0
0.2
0.4
0.6
0.8
1.0
Power (n=25)
Complex Categorical 1000 Genes 100 Sets
Level (n=25)
Complex Categorical 1000 Genes 100 Sets
Power (n=25)

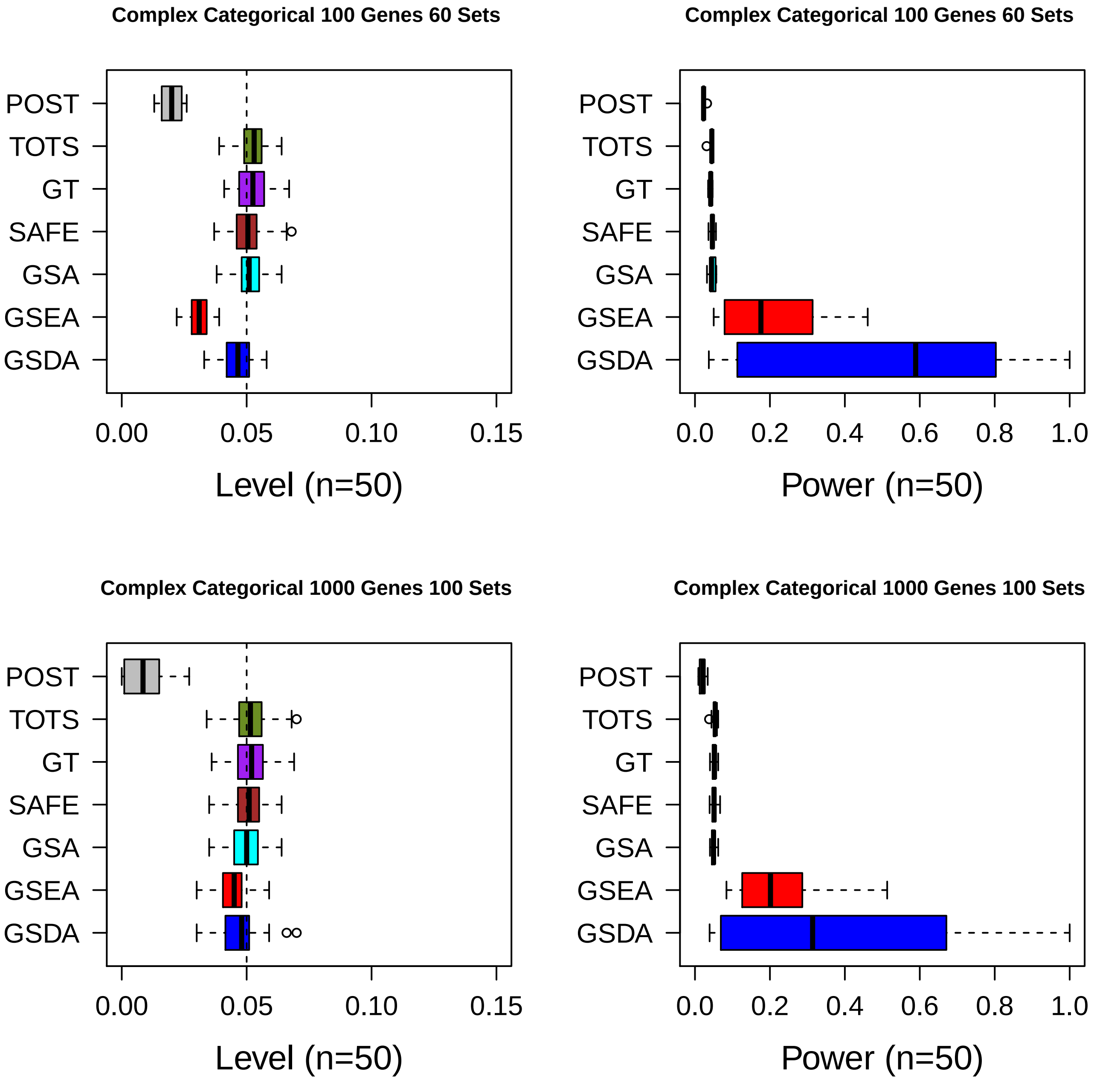
Complex Categorical 100 Genes 60 Sets
POST
TOTS
GT
SAFE
GSA
GSEA
GSDA
0.00
0.05
0.10
0.15
Level (n=50)
Complex Categorical 100 Genes 60 Sets
POST
TOTS
GT
SAFE
GSA
GSEA
GSDA
0.0
0.2
0.4
0.6
0.8
1.0
Power (n=50)
Complex Categorical 1000 Genes 100 Sets
POST
TOTS
GT
SAFE
GSA
GSEA
GSDA
0.00
0.05
0.10
0.15
Level (n=50)
Complex Categorical 1000 Genes 100 Sets
POST
TOTS
GT
SAFE
GSA
GSEA
GSDA
0.0
0.2
0.4
0.6
0.8
1.0
Power (n=50)

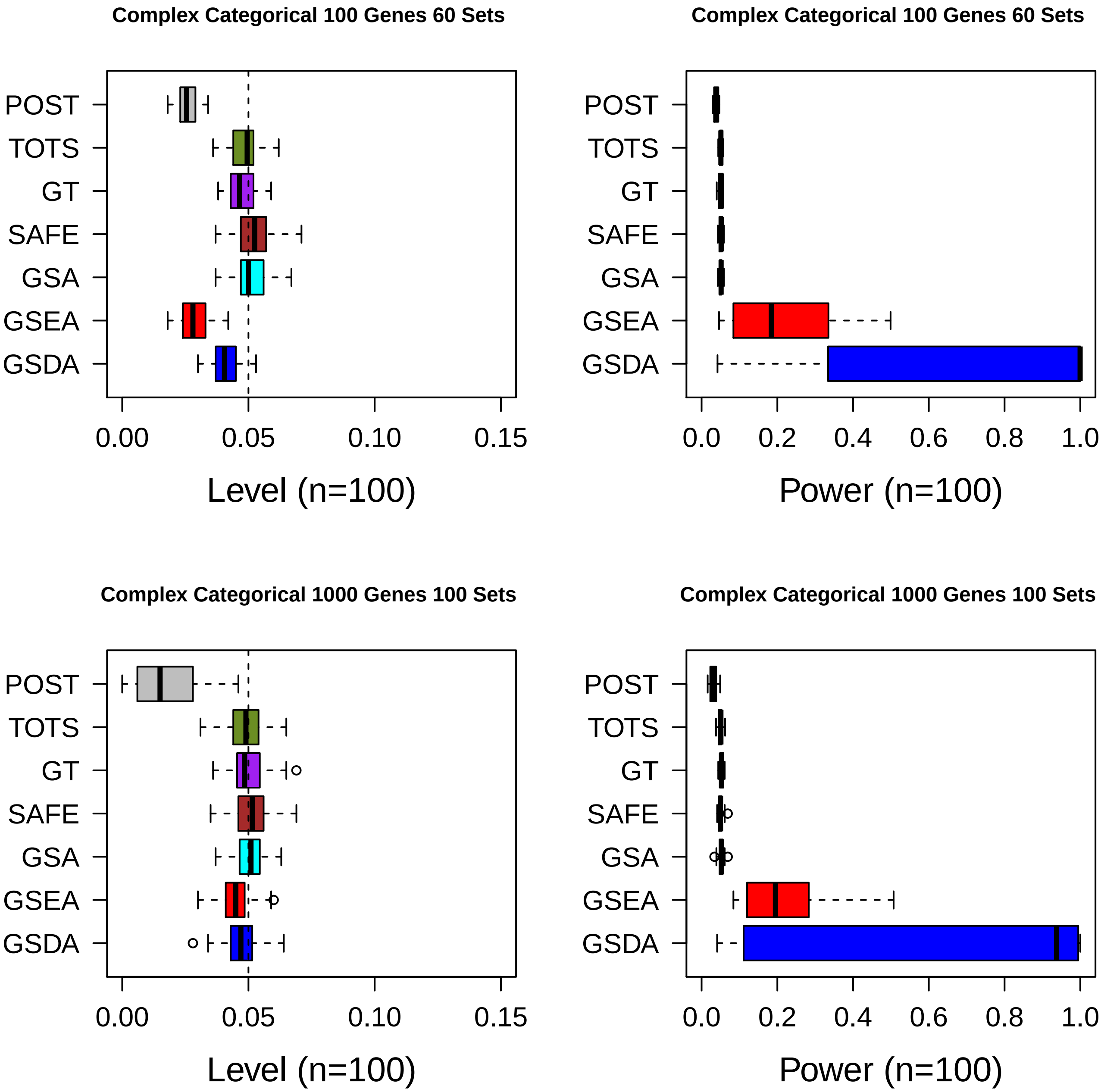
Complex Categorical 100 Genes 60 Sets
POST
TOTS
GT
SAFE
GSA
GSEA
GSDA
0.00
0.05
0.10
0.15
Level (n=100)
Complex Categorical 100 Genes 60 Sets
0.0
0.2
0.4
0.6
0.8
1.0
Power (n=100)
Complex Categorical 1000 Genes 100 Sets
Level (n=100)
Complex Categorical 1000 Genes 100 Sets
Power (n=100)

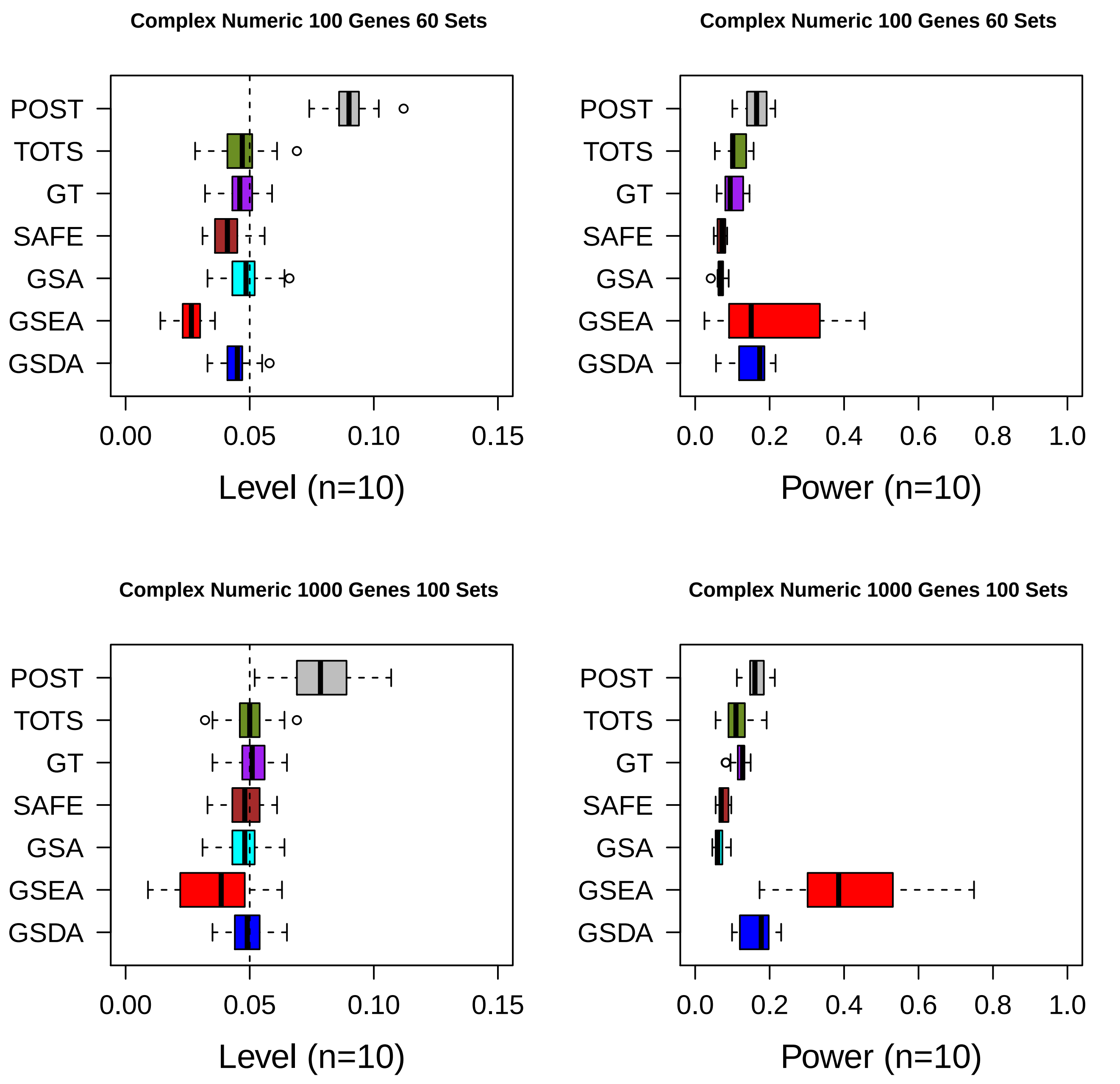

Complex Numeric 100 Genes 60 Sets
POST
TOTS
GT
SAFE
GSA
GSEA
GSDA
0.00
0.05
0.10
0.15
Level (n=10)
Complex Numeric 100 Genes 60 Sets
0.0
0.2
0.4
0.6
0.8
1.0
Power (n=10)
Complex Numeric 1000 Genes 100 Sets
Level (n=10)
Complex Numeric 1000 Genes 100 Sets
Power (n=10)

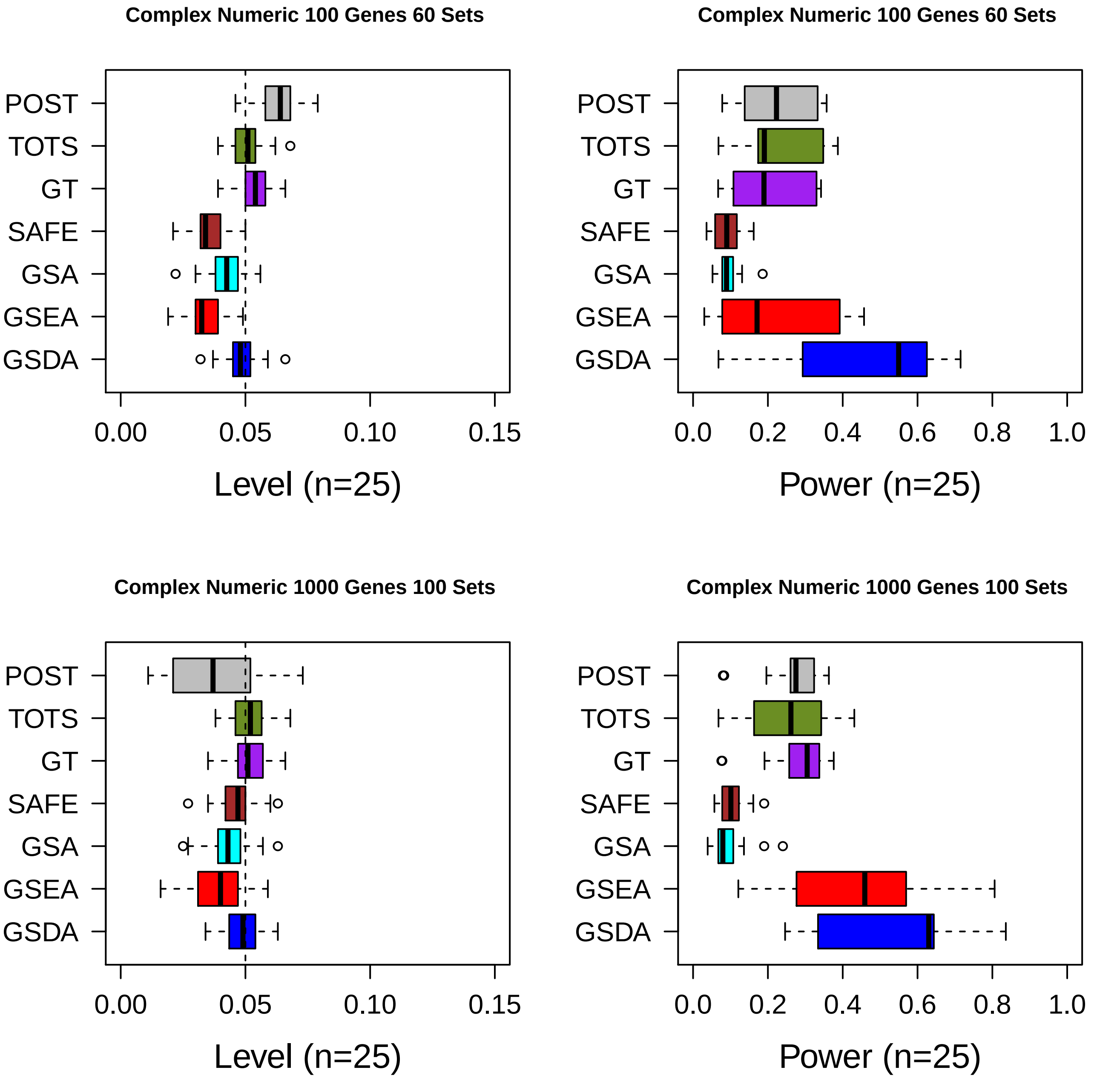
Complex Numeric 100 Genes 60 Sets
POST
TOTS
GT
SAFE
GSA
GSEA
GSDA
0.00
0.05
0.10
0.15
Level (n=25)
Complex Numeric 100 Genes 60 Sets
0.0
0.2
0.4
0.6
0.8
1.0
Power (n=25)
Complex Numeric 1000 Genes 100 Sets
Level (n=25)
Complex Numeric 1000 Genes 100 Sets
Power (n=25)

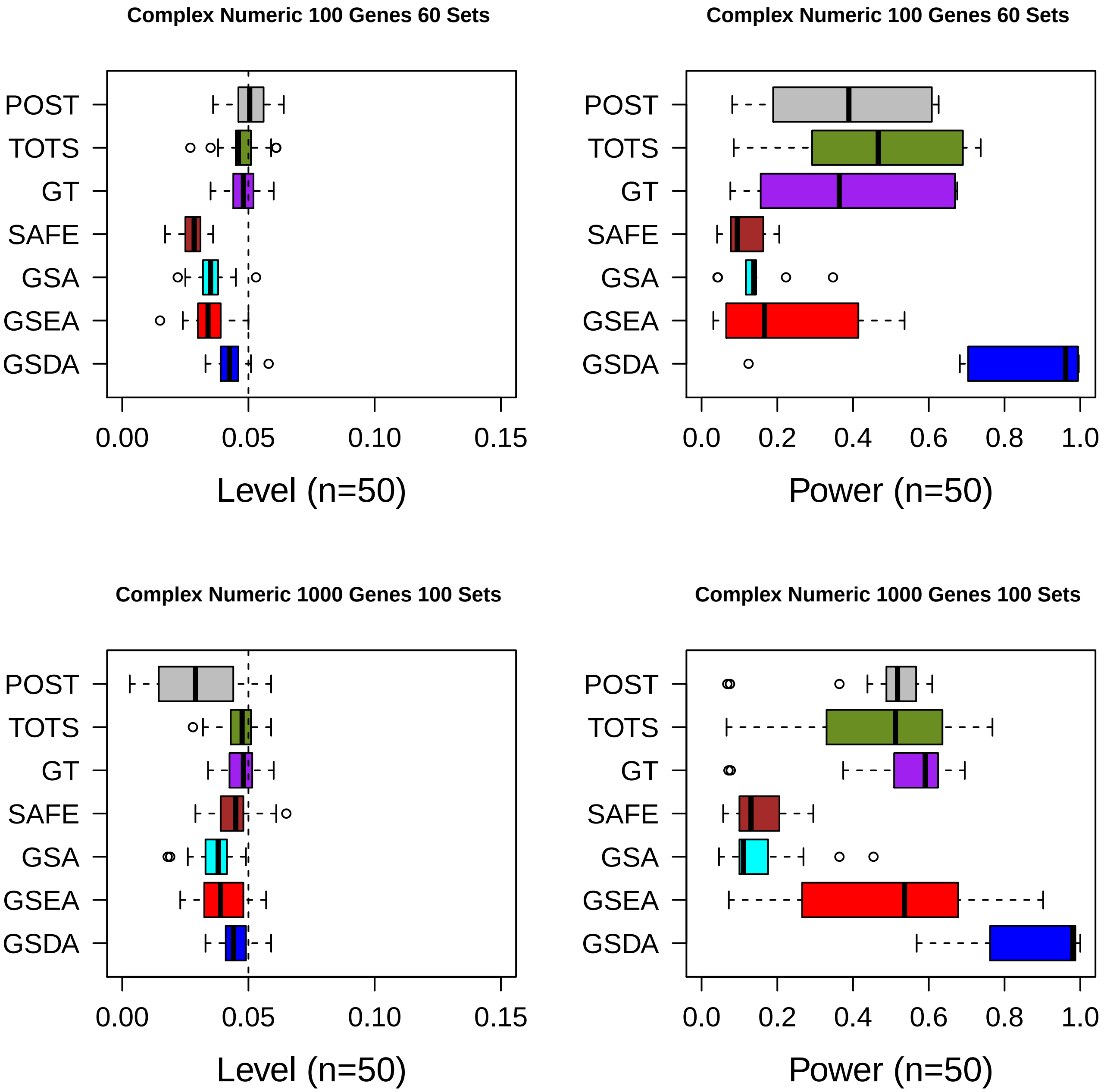
Complex Numeric 100 Genes 60 Sets
POST
TOTS
GT
SAFE
GSA
GSEA
GSDA
0.00
0.05
0.10
0.15
Level (n=50)
Complex Numeric 100 Genes 60 Sets
0.0
0.2
0.4
0.6
0.8
1.0
Power (n=50)
Complex Numeric 1000 Genes 100 Sets
Level (n=50)
Complex Numeric 1000 Genes 100 Sets
Power (n=50)

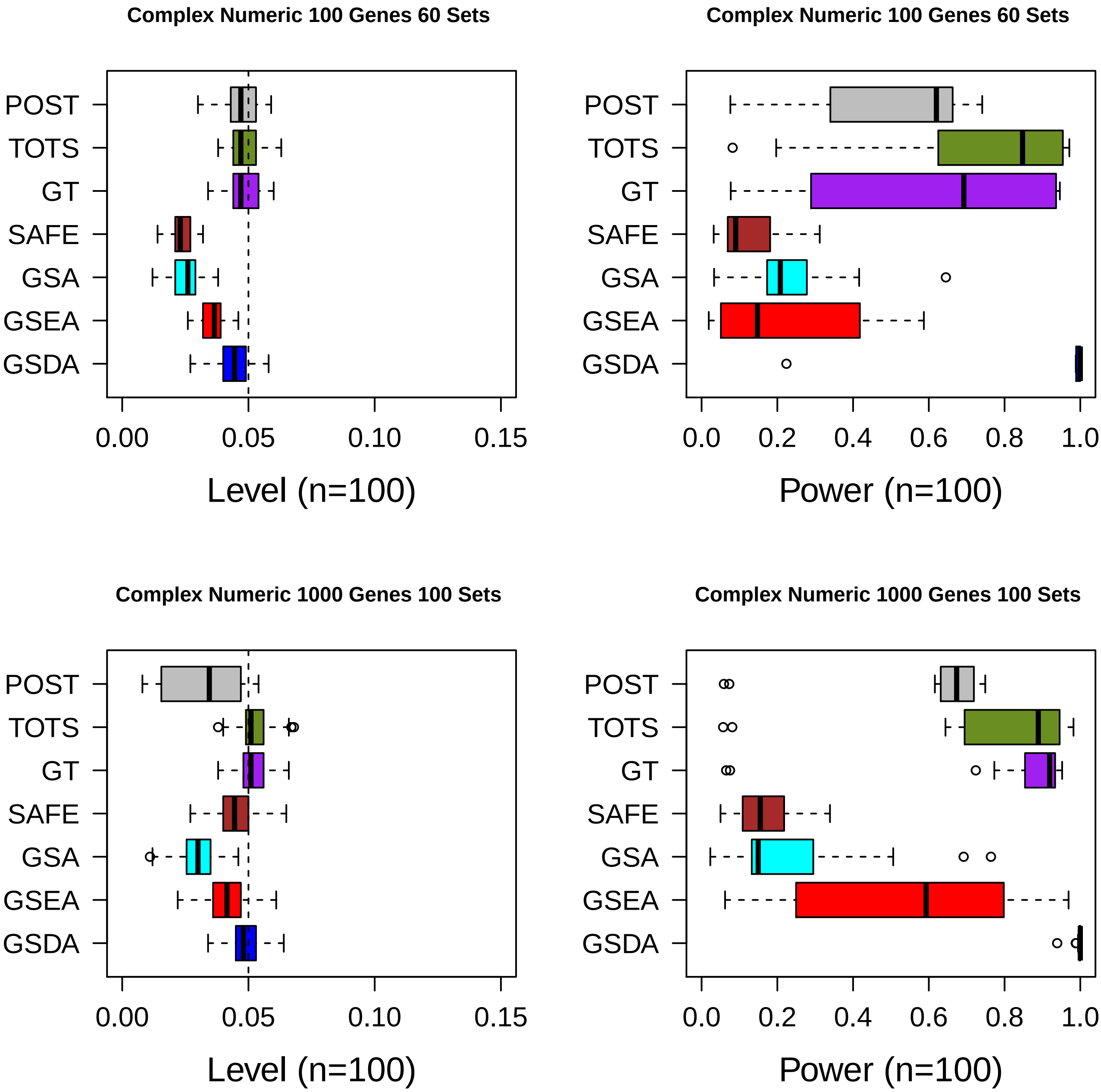
Complex Numeric 100 Genes 60 Sets
POST
TOTS
GT
SAFE
GSA
GSEA
GSDA
0.00
0.05
0.10
0.15
Level (n=100)
Complex Numeric 100 Genes 60 Sets
POST
TOTS
GT
SAFE
GSA
GSEA
GSDA
0.0
0.2
0.4
0.6
0.8
1.0
Power (n=100)
Complex Numeric 1000 Genes 100 Sets
POST
TOTS
GT
SAFE
GSA
GSEA
GSDA
0.00
0.05
0.10
0.15
Level (n=100)
Complex Numeric 1000 Genes 100 Sets
POST
TOTS
GT
SAFE
GSA
GSEA
GSDA
0.0
0.2
0.4
0.6
0.8
1.0
Power (n=100)

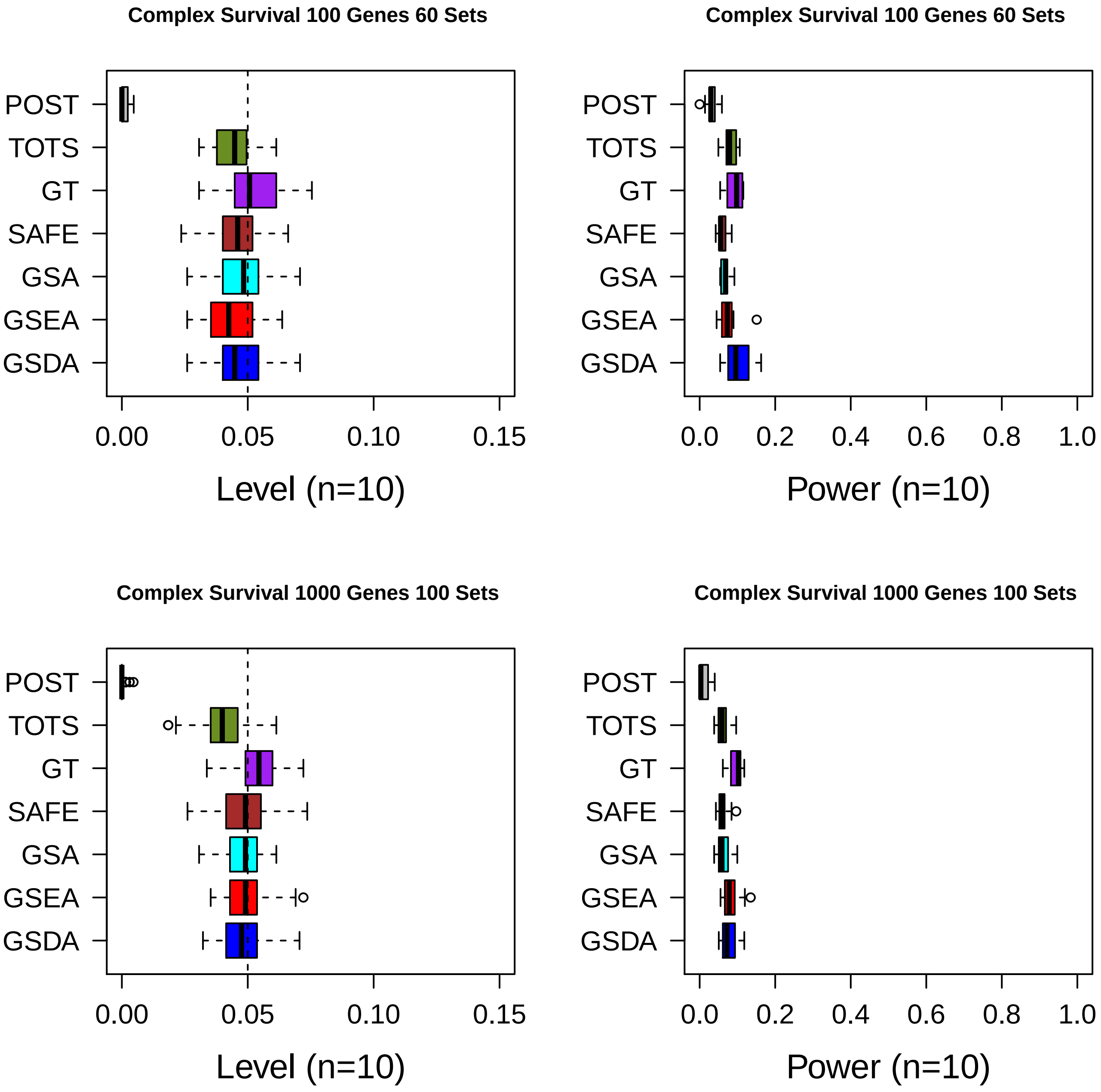
Complex Survival 100 Genes 60 Sets
POST
TOTS
GT
SAFE
GSA
GSEA
GSDA
0.00
0.05
0.10
0.15
Level (n=10)
Complex Survival 100 Genes 60 Sets
0.0
0.2
0.4
0.6
0.8
1.0
Power (n=10)
Complex Survival 1000 Genes 100 Sets
Level (n=10)
Complex Survival 1000 Genes 100 Sets
Power (n=10)

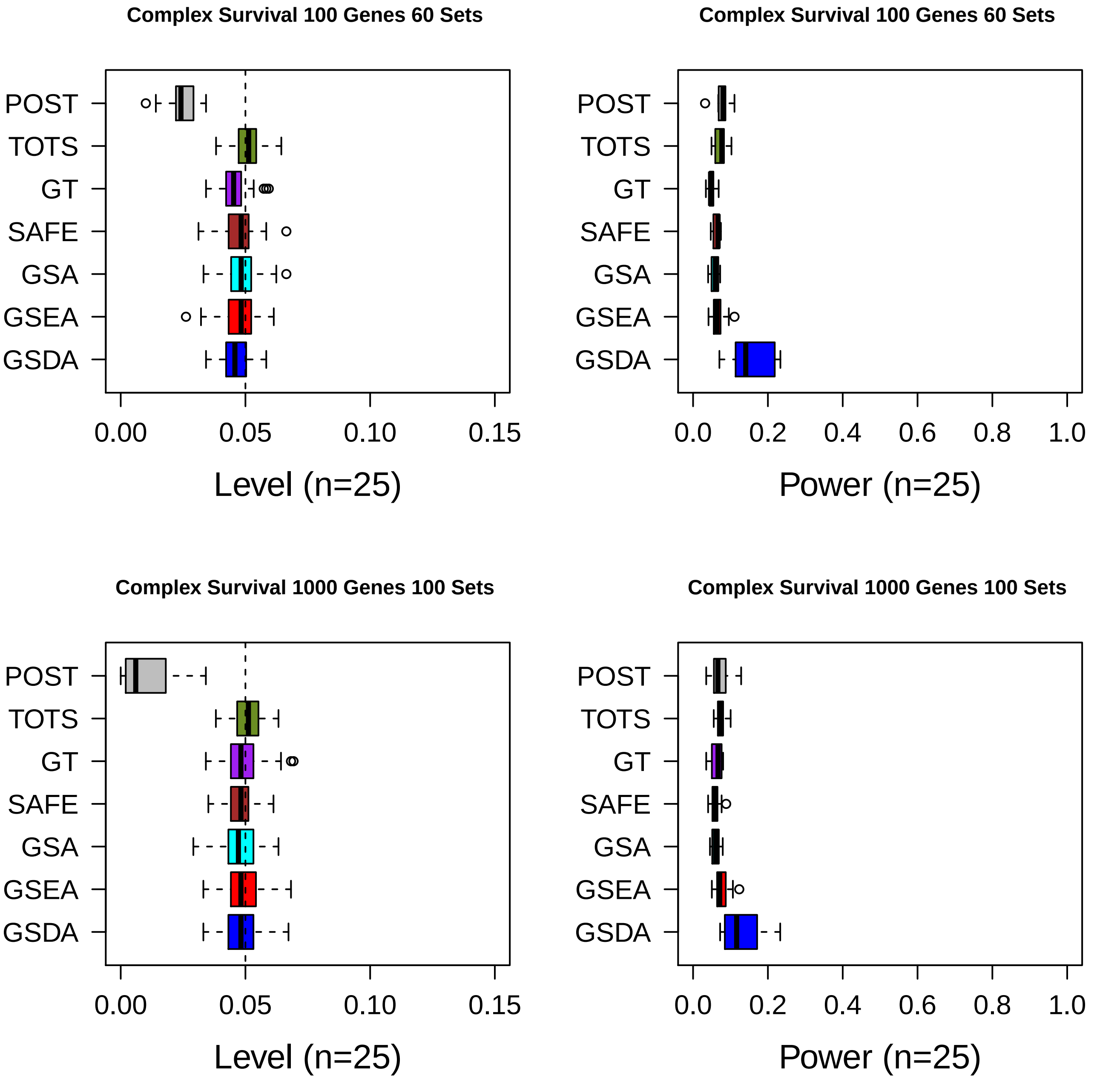
Complex Survival 100 Genes 60 Sets
POST
TOTS
GT
SAFE
GSA
GSEA
GSDA
0.00
0.05
0.10
0.15
Level (n=25)
Complex Survival 100 Genes 60 Sets
0.0
0.2
0.4
0.6
0.8
1.0
Power (n=25)
Complex Survival 1000 Genes 100 Sets
Level (n=25)
Complex Survival 1000 Genes 100 Sets
Power (n=25)

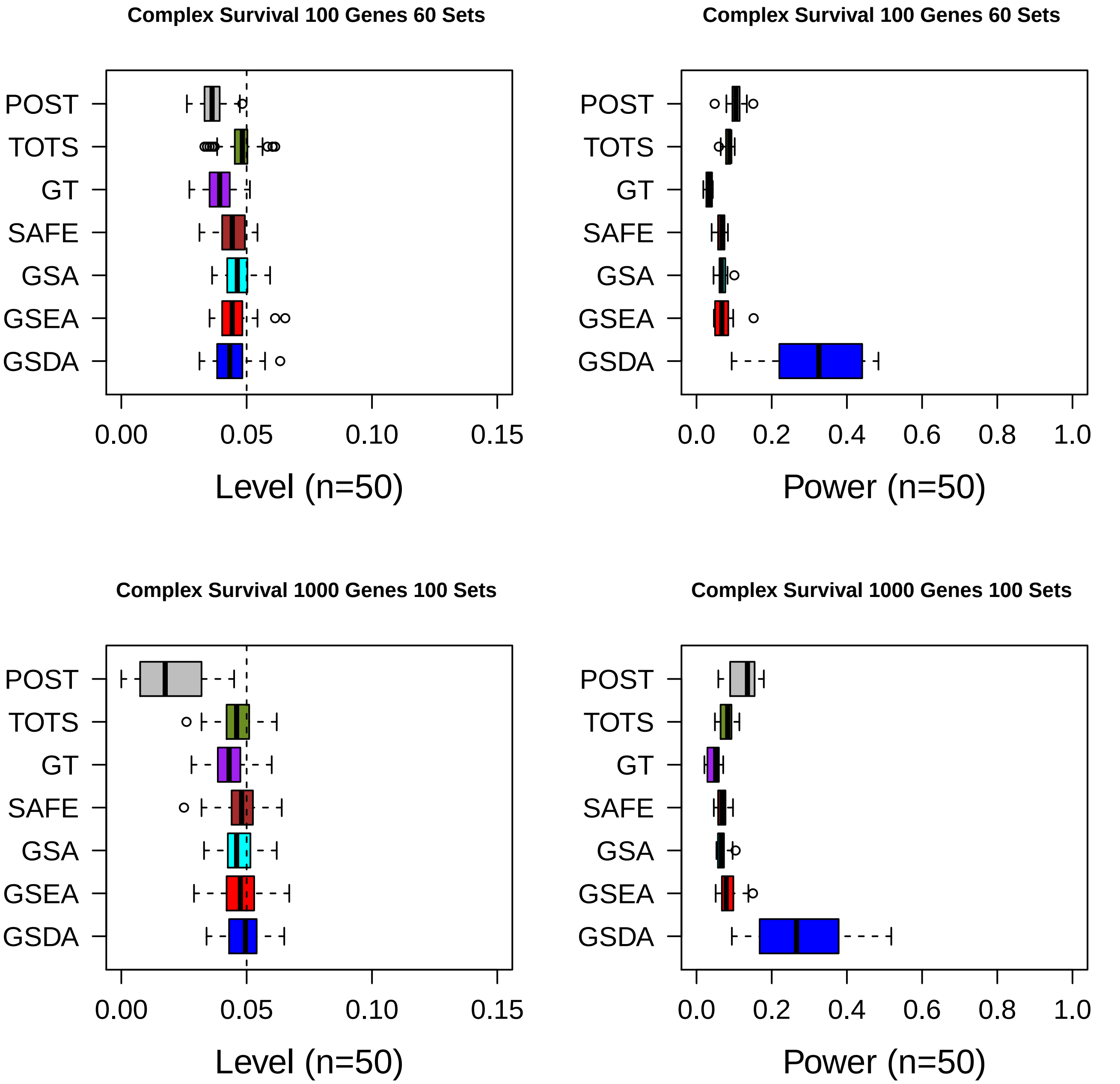
Complex Survival 100 Genes 60 Sets
POST
TOTS
GT
SAFE
GSA
GSEA
GSDA
0.00
0.05
0.10
0.15
Level (n=50)
Complex Survival 100 Genes 60 Sets
0.0
0.2
0.4
0.6
0.8
1.0
Power (n=50)
Complex Survival 1000 Genes 100 Sets
Level (n=50)
Complex Survival 1000 Genes 100 Sets
Power (n=50)

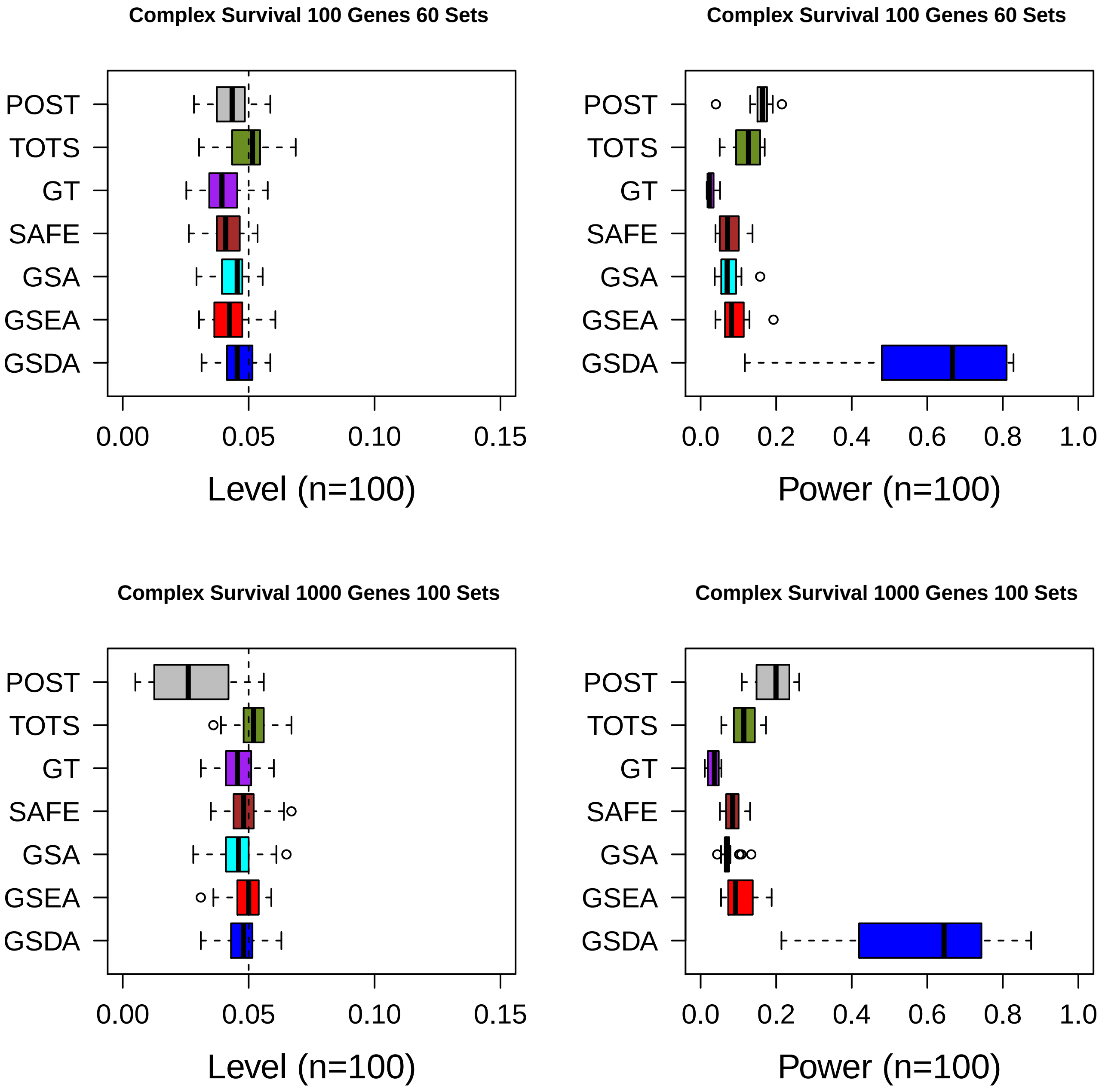
Complex Survival 100 Genes 60 Sets
POST
TOTS
GT
SAFE
GSA
GSEA
GSDA
0.00
0.05
0.10
0.15
Level (n=100)
Complex Survival 100 Genes 60 Sets
POST
TOTS
GT
SAFE
GSA
GSEA
GSDA
0.0
0.2
0.4
0.6
0.8
1.0
Power (n=100)
Complex Survival 1000 Genes 100 Sets
POST
TOTS
GT
SAFE
GSA
GSEA
GSDA
0.00
0.05
0.10
0.15
Level (n=100)
Complex Survival 1000 Genes 100 Sets
POST
TOTS
GT
SAFE
GSA
GSEA
GSDA
0.0
0.2
0.4
0.6
0.8
1.0
Power (n=100)
