# Supplementary figures and images for: Gene-set distance analysis (GSDA): a powerful tool for gene-set association analysis
Source: BMC Bioinformatics. 2021 Apr 21;22:207. doi: 10.1186/s12859-021-04110-x (PMC8059024; doi:10.1186/s12859-021-04110-x)

# Completed Analyses Per Minute

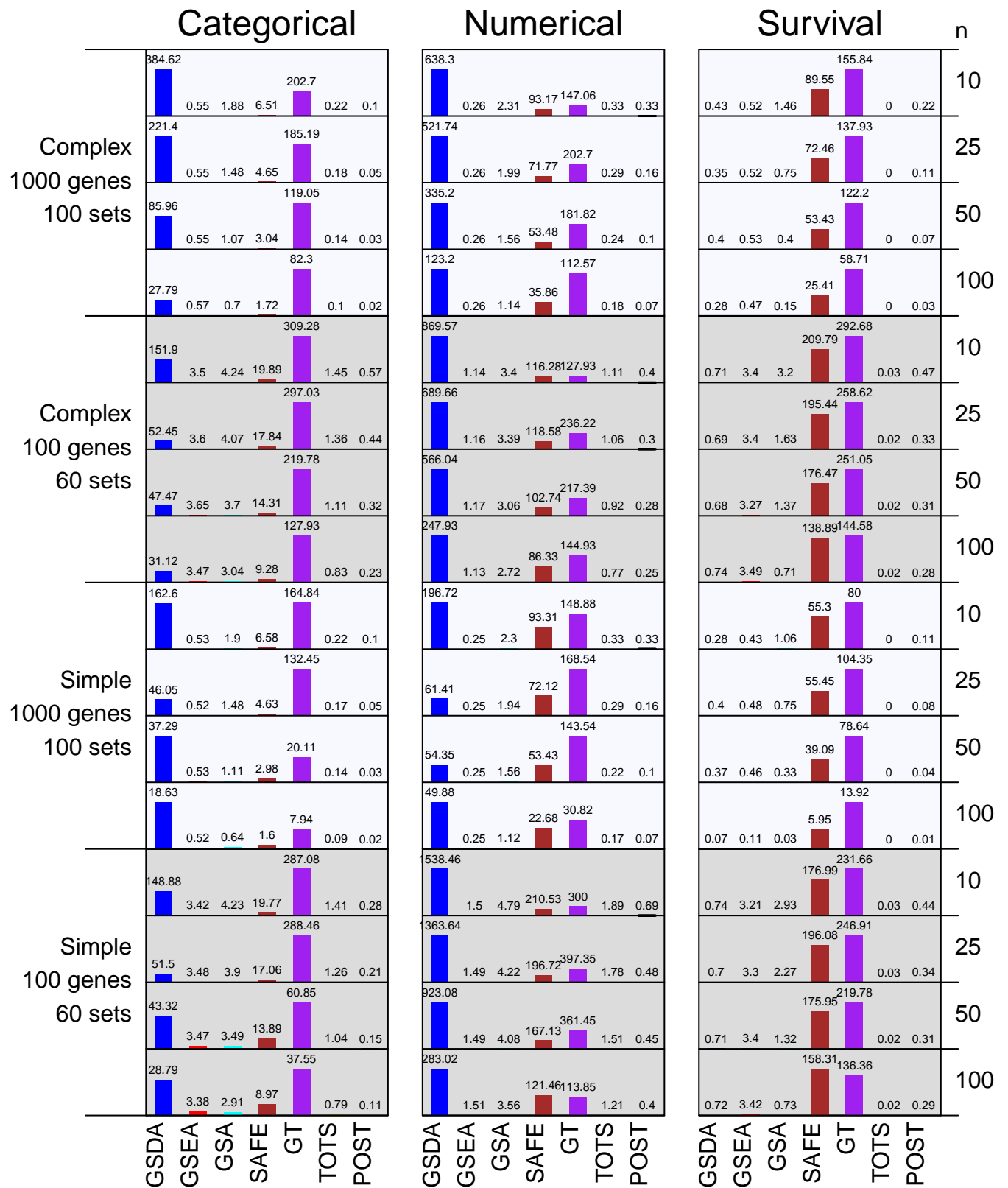

Supplement: Supplementary file 5 — Additional file 5. This supplementary file contains bar plots of the compute speeds (number ofcompleted analyses per minute) for each method in each of the 48 scenarios. [file 12859_2021_4110_MOESM5_ESM.pdf]
